# Supplementary material for: Mapping the function of neuronal ion channels in model and experiment
Source: eLife. 2017 Mar 6;6:e22152. doi: 10.7554/eLife.22152 (PMC5340531; doi:10.7554/eLife.22152)
Supplement: Supplementary file 1. — This file contains a brief tutorial which introduces all of the major aspects of the accompanying web browser for the database. DOI: http://dx.doi.org/10.7554/eLife.22152.020 [file elife-22152-supp1.pdf]

Rich media supplement:
http://icg.neurotheory.ox.ac.uk/viewer

Note to eLife editors: We wish to submit the accompanying website http://icg.neurotheory.ox.ac.uk/viewer as a "rich media" supplement to our manuscript submission.

This is a simple instruction manual for the IonChannelGenealogy Browser found on the website http://icg.neurotheory.ox.ac.uk/viewer. Please refer to the corresponding publication for more background information. This manual was last updated on Nov. 28, 2016. Please submit a ticket http://icg.neurotheory.ox.ac.uk/contribute if you notice any errors or would like to suggest any additions.

Contents

1 Overall layout **2**

2 Selecting a model **4**

3 Type Selection **7**

3.1 Hierarchy view 7

3.2 XY view 10

3.3 Similarity view 10

3.4 Cluster view 10

4 Family Selection **11**

5 Color Selection **11**

6 Filter Selection **12**

7 Compare Models **15**

8 Examples **16**
8.1 Example 1: Search for a specific ModelDB entry or author 16
8.2 Example 2: Compare all models of a particular subtype 17
8.3 Example 3: Search for a specific brain area and neuron type . . . 20

1 Overall layout

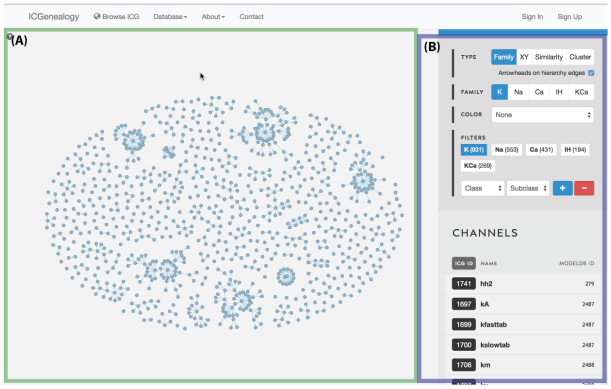

Figure 1: Overall layout of the ICG Browser. A: main visualization space, with data displayed as selected in B. B: toolbar for manipulating the displayed data, including filtering and search.

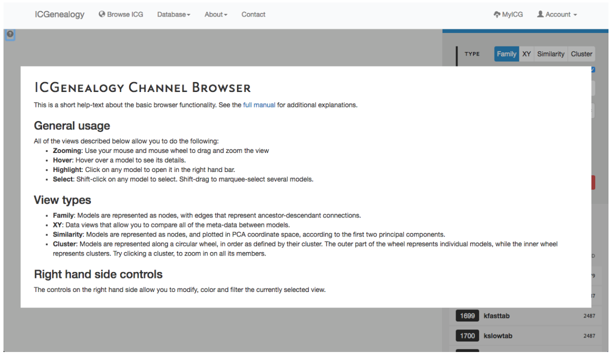

Figure 2: Help screen of the ICG Browser. Appears when the question mark button at the top left is clicked.

The ICG Browser is composed of a main visualization space (Figure 1, A) and a toolbar on the right (Figure 1, B). The visualization space can be manipulated by zooming in and out using a standard mouse scroll or trackpad scroll, as well

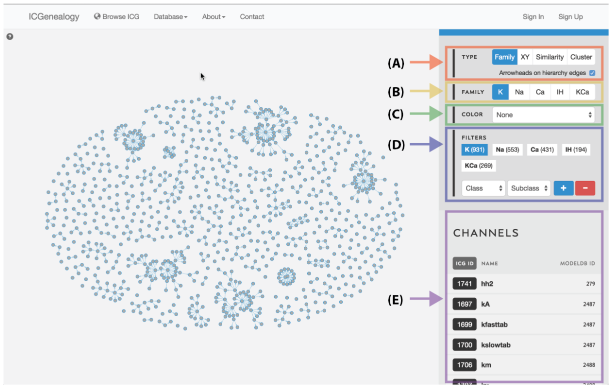

Figure 3: Outline of the ICG toolbar: type selection (A), family selection (B), color selection (C), filter selection (D), and channel list (E).

as `drag+click`. A help button in the top left corner of the visualization space provides some basic information about the browser (Figure 2). Note that the right toolbar scrolls downwards to display more information. The items found in the right toolbar are highlighted in Figure 3, which will each be described in turn:

A. **Type Selection**: select between four different data viewers: hierarchy view, XY view, similarity view, and cluster view. Additional options for the current view, found below the view option box, can be turned on and off with the check box. See section 3 for more details.

B. **Family Selection**: select among five ion type families: voltage-dependent potassium (K), voltage-dependent sodium (Na), voltage-dependent calcium (Ca), calcium-dependent potassium (KCa), and voltage-dependent afterhyperpolarization-activated cation (Ih). All four data views in type selection can be used for each family. Note that only the K family is shown in this instruction manual. See section 4 for more details.

C. **Color Selection**: select coloring of nodes by a metadata item. See section 5 for more details.

D. **Filter Selection**: filter the currently visible nodes by a particular metadata item. See section 6.

E. **Channel list**: list of all channel models currently being displayed, with id, mod name, and modelDB id. Scroll down to view. Click on a model to highlight node in visualization space and to go to channel details. See section 2 for more details.

2 Selecting a model

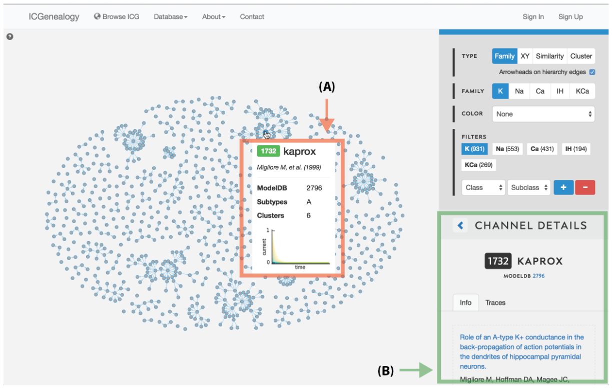

Figure 4: Selecting a model. A: a tooltip appears when the mouse hovers over a model in the visualization space, containing the following items: model ID, model name, reference, modelDB ID, subtypes, clusters and a small image of the traces.

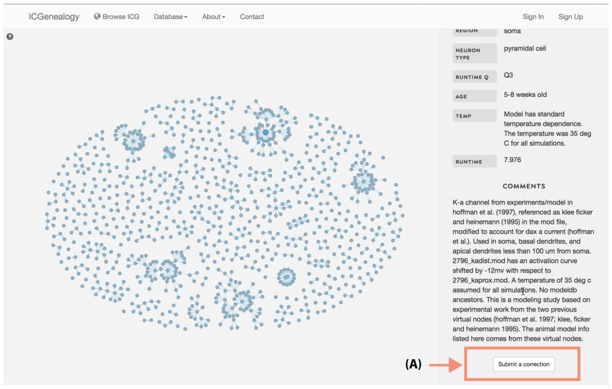

Figure 5: Submit a correction. At the bottom of channel details for each model.

Models can be selected by clicking on individual items in the visualization space in any of the three views. Hovering over a model brings up a tooltip box (Figure 4, **A**) which contains a small summary of the model, including *model ID*, *model name*, *publication reference*, *modelDB ID*, *subtype*, *cluster membership* and a small image of the *current response traces*.

Clicking on a model brings up the *Channel Details* on the right (Figure 4, **B**) in place of the channel list. The channel details lists all metadata information and is described below:

- **ID**: unique identification number for each channel model on ICGenealogy

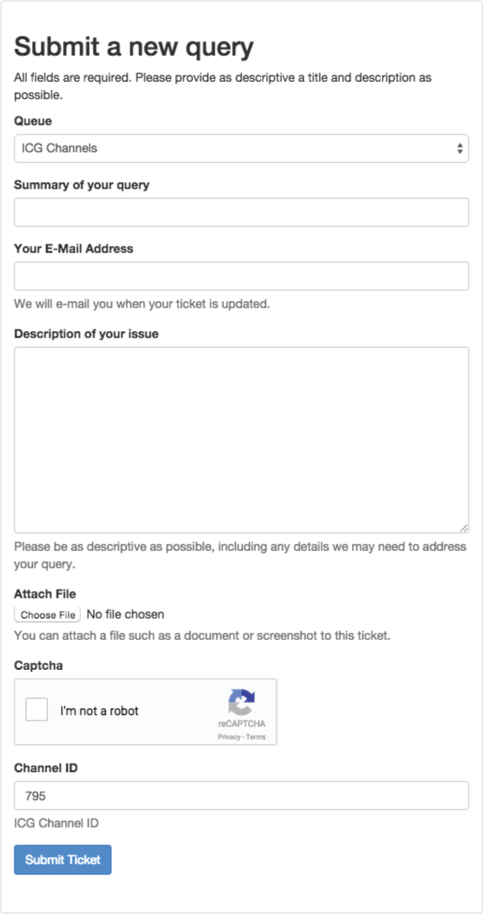

Figure 6: Submit a new query. This webpage comes up when the Submit a correction button is pressed. Queries contain a summary, corresponding email address, description of the issue and the option of attaching a file. If the issue concerns a particular channel model, this ID may be entered below. By clicking on the Submit a correction button at the bottom of Channel Details for a particular model, this modelâĂŹs ID will be autofilled into the Channel ID field.

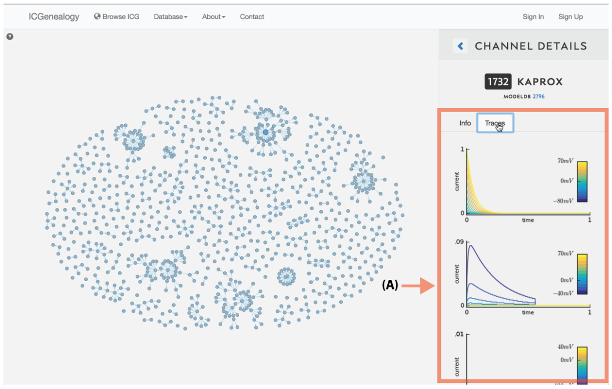

Figure 7: Simulation traces. Each modelâĂŹs current response traces can be viewed by clicking on the Traces tab in Channel Details.

- **Name**: file name of the mod file, as taken from ModelDB entry
- **ModelDB ID**: Identification number associated with each entry on ModelDB. All channel models from the same entry have the same ID number.
- **Reference Information**: lists the corresponding journal article for the model, with link to PubMed page (if exists), as well as the number of citations (from google scholar)
- **Ion Type**: ion permeability of the channel model; must be one of the following: potassium (K), sodium (Na), calcium (Ca), nonspecific, depending on the Family Selection.
- **Subtype**: subtype of the ion channel model, as listed in the journal article and/or mod file itself.
- **Animal Model**: The emulated animal model of the simulation, as either stated explicitly, or inferred from the journal article.
- **Brain Area**: the emulated brain area and layer of the simulation, as stated explicitly, or inferred from the journal article.
- **Neuron Area**: The neuron area that the ion channel is found in, divided into dendrites, soma, axon, axon hillock, or specific areas of dendrites and axon.
- **Neuron Type**: The emulated neuron type of the simulation. May be several types, or general if no neuron type was specified.
- **Runtime Q**: Elapsed CPU time for running 10 repetitions of a single voltage-clamp protocol (action-potential), divided into four quartiles of the distribution of runtimes of all models in each class.
- **Age**: animal model age, if specified
- **Temp**: Details about the model's temperature dependence, and also the temperature at which simulations and/or experiments were performed as described in the journal article.
- **Runtime**: Elapsed CPU time for running 10 repetitions of a single voltage-clamp protocol (action-potential)

- **Comments**: Comments from the mod files themselves and any other information about the channel and model from the journal article, such as previous models or experimental data that were used to build the model.

Submit a correction: At the bottom of Channel details, there is a button which can be used to submit a correction (Figure 5). This can be used to report and missing or incorrect information about this channel model. Furthermore, if any information is incorrect that is not related to a specific channel, it can also be listed here. Clicking on the button brings up a new page which allows the user to submit a ticket (Figure 6).

Simulation traces: Just under the channel ID and name, is a clickable tab that shows the simulation traces for the chosen model (Figure 7). Traces are shown for the five simulation protocols: *activation*, *inactivation*, *deactivation*, *action potential*, and *ramp*. The first three protocols have multiple voltage step values, as indicated by the colorbars on the right. Note that the entire protocol is not shown - only the relevant part of each protocol is plotted, as described in the corresponding publication.

3 Type Selection

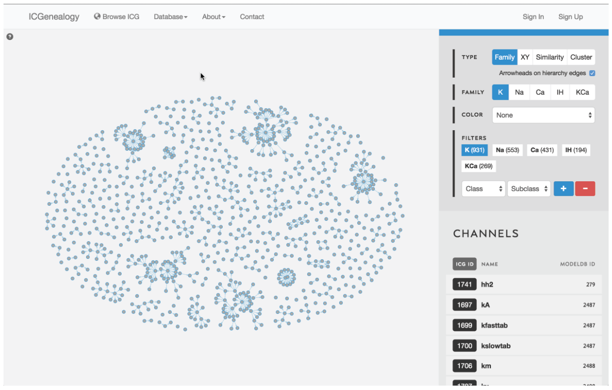

Figure 8: Type selection: hierarchy view.

There are four ways of viewing the data: hierarchy view (Figure 8), XY view (Figure 9), similarity view (Figure 10) and cluster view (Figure 11).

3.1 Hierarchy view

Models are represented as nodes, with edges that represent ancestor-descendant connections (see publication). Groups of models are displayed in dynamically

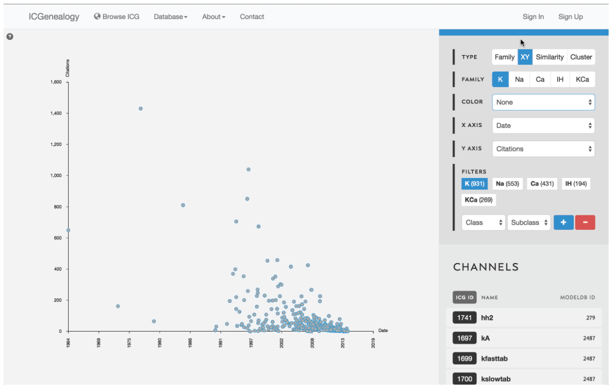

Figure 9: Type selection: XY view.

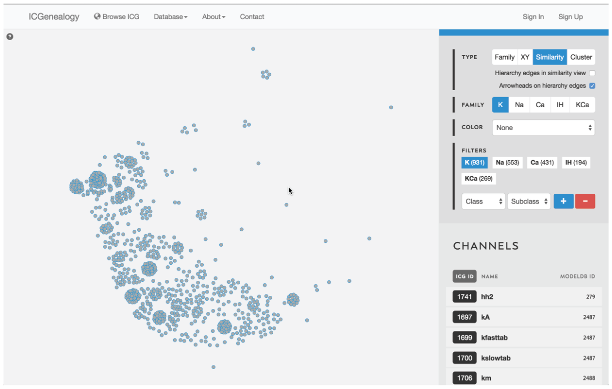

Figure 10: Type selection: similarity view.

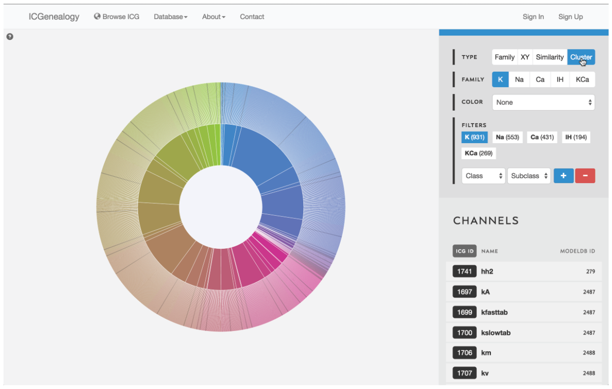

Figure 11: Type selection: cluster view.

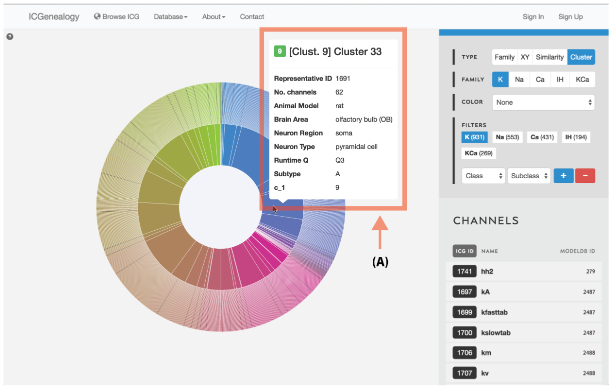

Figure 12: Type selection: cluster view tooltip for cluster information.

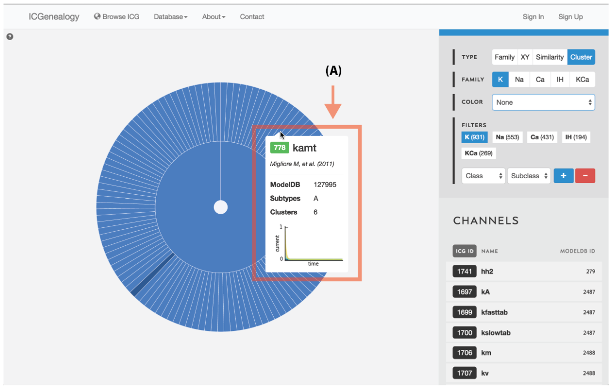

Figure 13: Type selection: cluster collapse and tooltip.

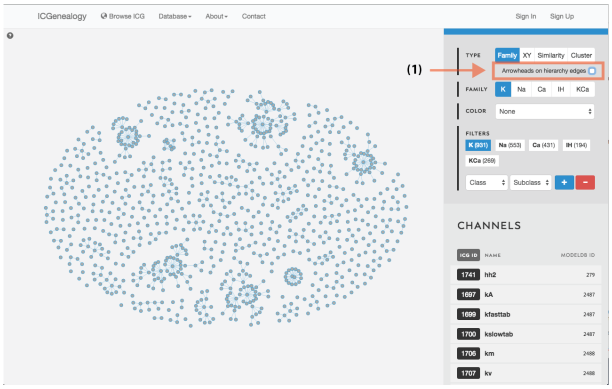

Figure 14: Hierarchy view: selection of directional edges.

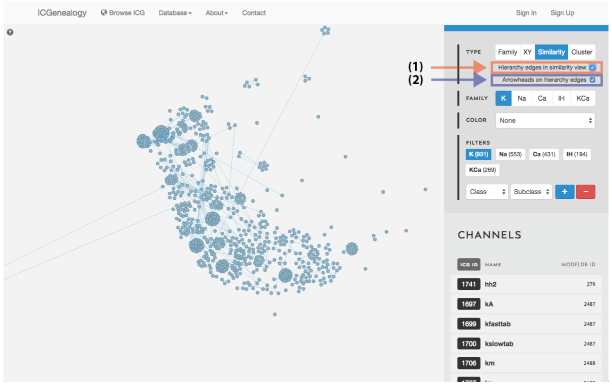

Figure 15: Similarity view: selection of hierarchy edges and direction.

placed arbitrary locations. Hierarchy view has the additional option of adding directional arrowheads to the edges, pointing from ancestor to descendant (Figure 14).

3.2 XY view

Models are represented as nodes and plotted along two metadata dimensions. Note the two extra fields in the available in the toolbar for selection of data.

3.3 Similarity view

Models are represented as nodes, and plotted in PCA coordinate space, according to the first two principal components. Similarity view has the option of adding ancestor-descendant edges, as well as directional arrowheads (Figure 15).

3.4 Cluster view

Models are represented along a circular wheel, in order as defined by their cluster. The outer part of the wheel represents individual models, while the inner wheel represents clusters. Collapsible clustering: In cluster view, clusters have a tooltip (Figure 12), and can also be selected, which collapses the cluster wheel and shows only models in the selected cluster (Figure 13). To return to the full view, click the circle in the middle.

4 Family Selection

Select among the five ion type families: voltage-dependent potassium (K), voltage-dependent sodium (Na), voltage-dependent calcium (Ca), calcium-dependent potassium (KCa), and voltage-dependent hyperpolarization-activated cation (Ih).

5 Color Selection

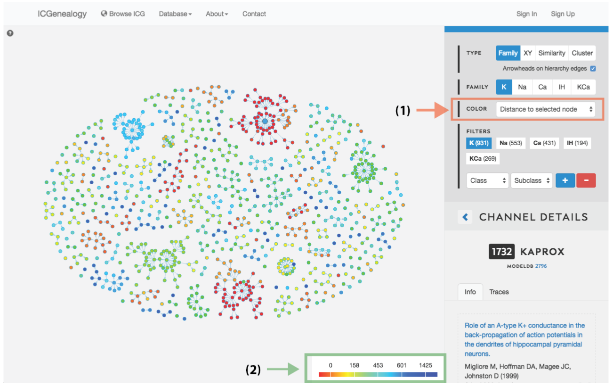

Figure 16: Color selection in hierarchy view: color nodes by a given metadata item as selected in (A). Legend appears in bottom right of visualization space (B). In this example, nodes are colored by distance to a selected model.

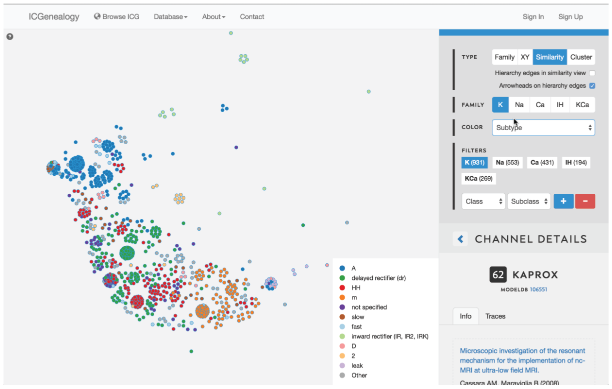

Figure 17: Color selection in similarity view: color nodes by a given metadata item as selected in (A). Legend appears in bottom right of visualization space (B). In this example, nodes are colored by subtype label.

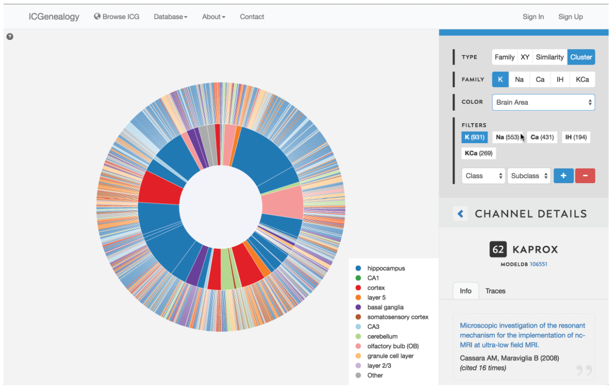

Figure 18: Color selection in cluster view: color nodes by a given metadata item as selected in (A). Legend appears in bottom right of visualization space (B). Note that clusters also receive colors based upon the most prevalent entry for the selected metadata item. In this example, nodes are colored by brain area.

This allows coloring nodes by any given metadata field, which can be done in all of the views (Figure 16, Figure 17, Figure 18). Legend appears in the bottom right. Note that some colors flicker when there is more than one entry for a given model. Furthermore, for items with greater than 12 possibilities, only the 11 most prevalent entries are shown in color, with all other entries grouped together in the group *Other*, colored in grey. In cluster view, the clusters themselves are also colored by the most prominent item for the selected metadata category (Figure 18). Color selection options are the same as metadata items listed in *Channel Details*, but a few extra items are described below:

- **Date**: Colorbar of publication year, from 1964 to 2014.
- **Distance**: Color by distance to a selected node. Red indicates small distance and blue indicates large distance.
- **Runtime Q**: Runtime quantile
- **Cluster Representative**: reference model for each cluster, defined as the model closest to the center of each cluster in score space.

6 Filter Selection

Filter the models that are currently being shown. We show some examples here. Filters can be added as a positive (Figure 19) or negative (Figure 20) condition. Positive conditions mean that only models with that metadata parameter are shown. A negative filter means that all models without that filter are shown. Any number of positive and negative filters can be added together (Figure 21). Filtering in cluster view does not reduce the number of models being shown,

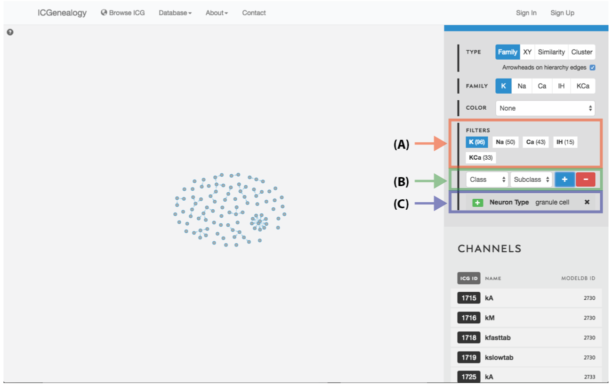

Figure 19: Filter selection: models have been filtered by the neuron type granule cell. Only models with this entry are shown in the visualization space. A: the number of models in each of the five families is listed. B: Option to add more filters. C: currently selected filter(s), with the option to remove.

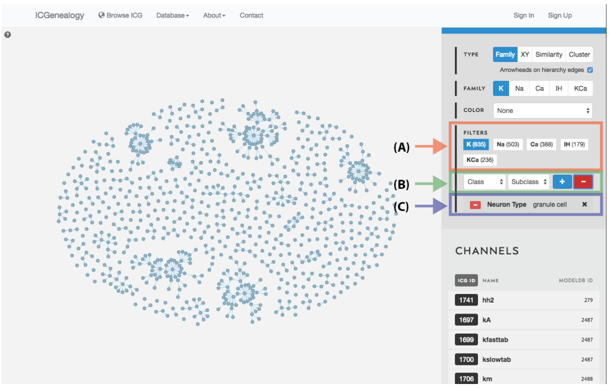

Figure 20: Filter selection: as in but with a negative filter.

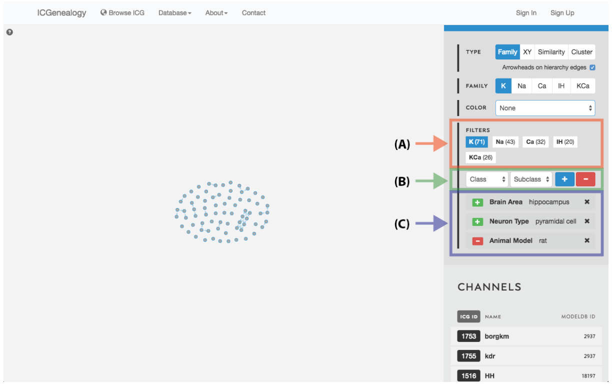

Figure 21: Filter selection: as in but with multiple filters.

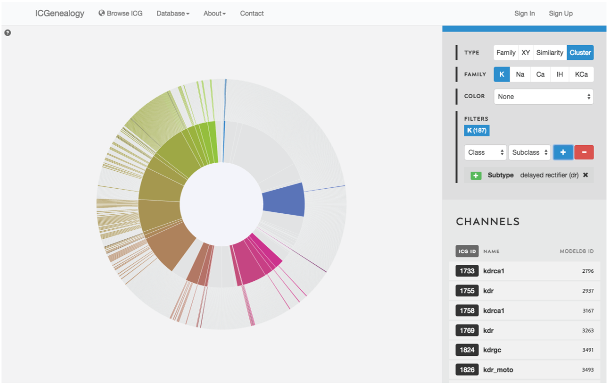

Figure 22: Filter selection in cluster view. Models that have been filtered out are faded.

but fades out the models that do not have that metadata attribute (Figure 22). Note also in cluster view that clusters remain visible if they have at least one model with that particular filter attribute.

7 Compare Models

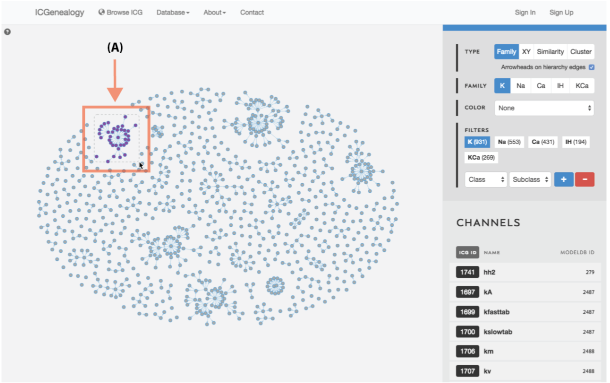

Figure 23: Compare models: shift+drag allows the selection of multiple models within a square window (A).

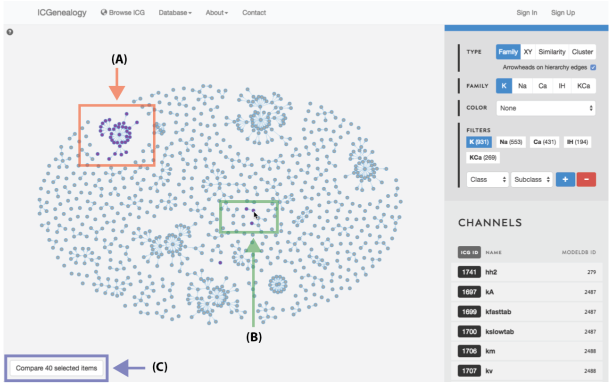

Figure 24: Compare models: shift+click allows the selection of individual models (A, B). C: button to compare currently selected models.

Select models by `shift+click` or `shift+drag` as shown in Figure 23 and Figure 24. Click the link at the bottom left: `compare X models`. This brings you to the compare page (Figure 25). To clear your current selection of models, `shift+click` on any whitespace area without a model.

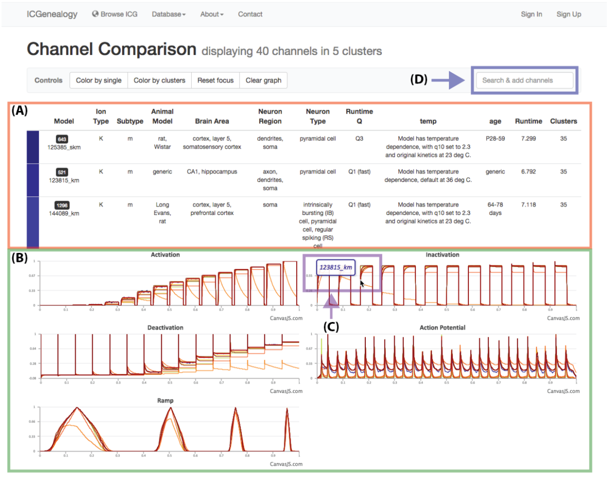

Figure 25: Compare models: channel comparison page. A,B: Shows summary comparison of all selected models, including metadata and current traces. C: Tooltip shows model name of trace. D: Option to add more channel models to the comparison.

8 Examples

8.1 Example 1: Search for a specific ModelDB entry or author

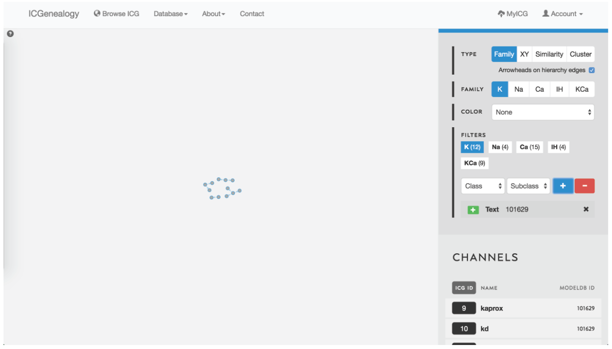

Figure 26: Example 1: search for a ModelDB ID using text search. Models in other ion type families can be selected from the filter panel.

Searching for ion channel models from a particular modelDB entry can be done in two ways. First of all, models are ordered in the *Channels List* at the bottom of the right toolbar when no model is selected (Figure 1 **B**). Alternatively, one

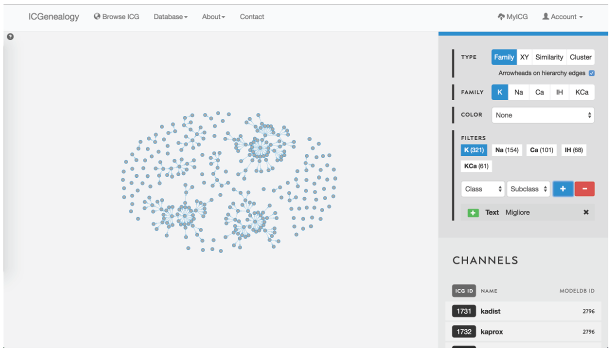

Figure 27: Example 2: search for author name using text search. This search finds matches in author name, reference and comments.

could take advantage of the text search filter to enter a modelDB entry, as illustrated in Figure 26 for ModelDB ID 101629. Note that the number of models in other ion type families is displayed in the *Filters* section as well.

Similarly, when searching for a particular author, the text search filter comes in handy. This is illustrated in Figure 27 for the last name *Migliore*.

8.2 Example 2: Compare all models of a particular subtype

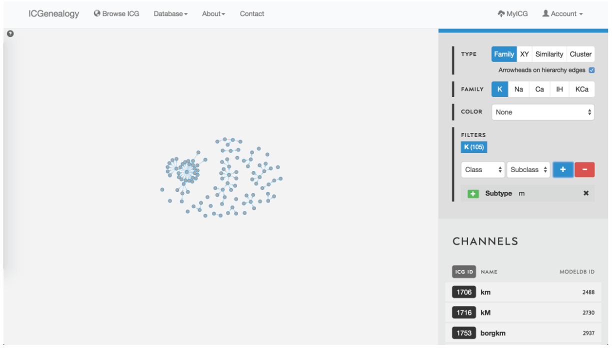

Figure 28: Example 2: filtering potassium channel models by the subtype label m-type.

In this example, let's say that you wanted to find a *m-type* potassium model. You would start by filtering the potassium models to only show models with subtype *m-type* (Figure 28). You would discover that there are 105 m-type models in the database. You then might choose to color the models by cluster,

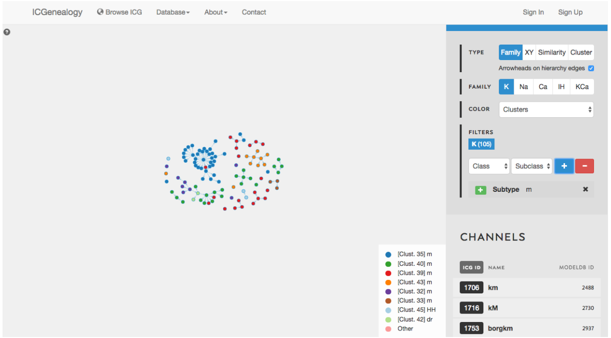

Figure 29: Example 2: coloring models by clusters.

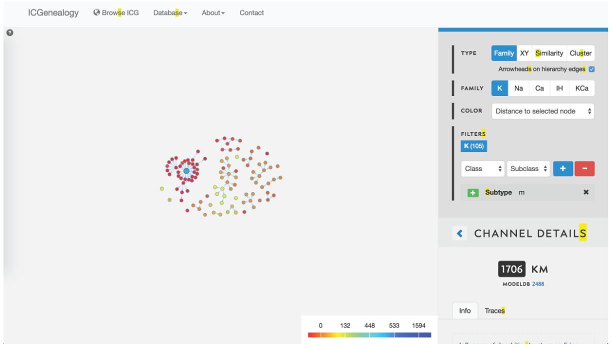

Figure 30: Example 2: coloring models by distance to selected model (in blue).

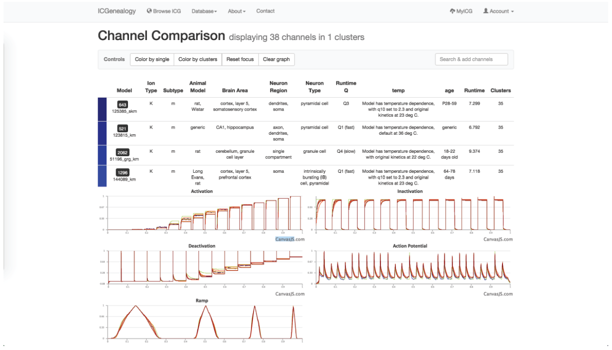

Figure 31: Example 2: models in dark blue cluster () have been selected to compare.

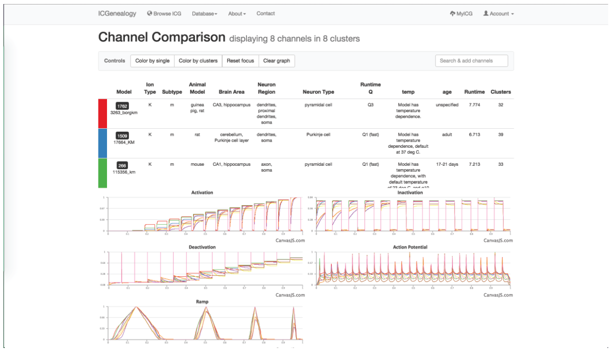

Figure 32: Example 2: one model from each of the eight clusters () has been selected to compare with the others.

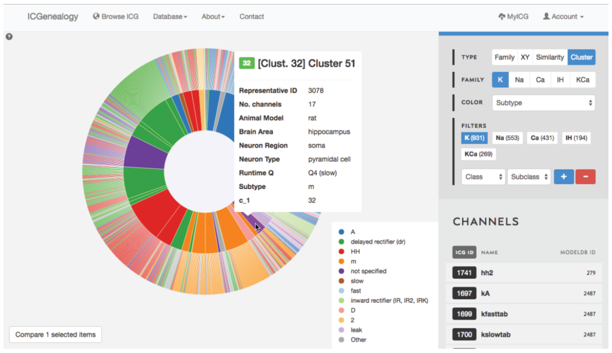

Figure 33: Example 2: cluster view is used to select m-type cluster.

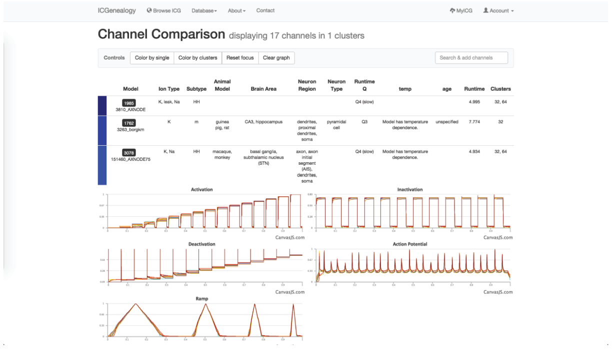

Figure 34: Example 2: All models in m-type cluster are compared together.

to see what is the diversity of kinetics (Figure 29). You see that there are eight different clusters.

You might be curious how similar different models in the dark blue cluster are to one another, so you instead choose to color by distance. You see that all m-type models in the blue cluster are very similar, nearly identical, and that all m-type models are quite similar (Figure 30). You filter out all clusters except for the dark blue one, to isolate these and then compare them together (Figure 29, Figure 31). Finally, you see the small differences in kinetics between the models, and the differences in metadata between the different models.

You are then interested to compare different m-type models together, so you go back to the browser and select one model from each of the eight different clusters to compare them (Figure 32). You see that there are several unique m-type behaviors, but also that some models do not resemble m-type as much.

Lastly, you might wonder if you are missing some models that might be m-type but not labelled as m-type. In order to find these, you turn to *cluster view* to find the clusters that are mostly m-type (Figure 33). You select one of these, and can then see several models with subtypes such as *HH* and *LVA* that also appear to have m-type behavior. You can then compare these models as well (Figure 34).

8.3 Example 3: Search for a specific brain area and neuron type

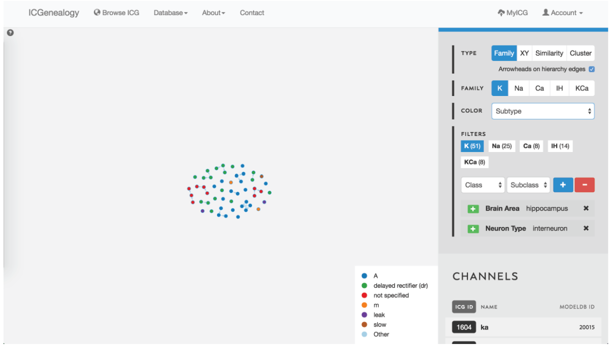

Figure 35: Example 3: filtering of models by brain area hippocampus and neuron type interneuron, and coloring by subtype.

In this example, let's say that you were making a model of a hippocampal interneuron, and you were interested in what ion channel models are available. You would start by filtering the available models down based on brain area and neuron type. You can then color the models by subtype to see that there are a few different subtypes available (Figure 35). You are curious how many behaviors

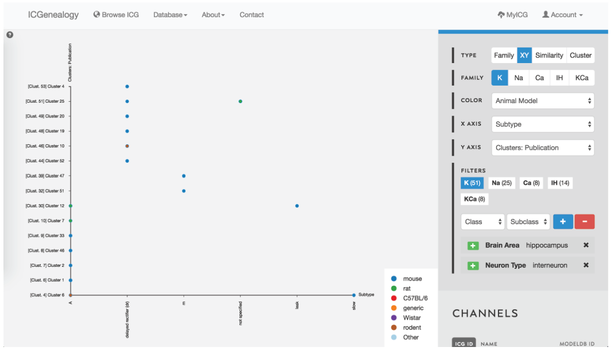

Figure 36: Example 3: XY view of models selected from , colored by animal model.

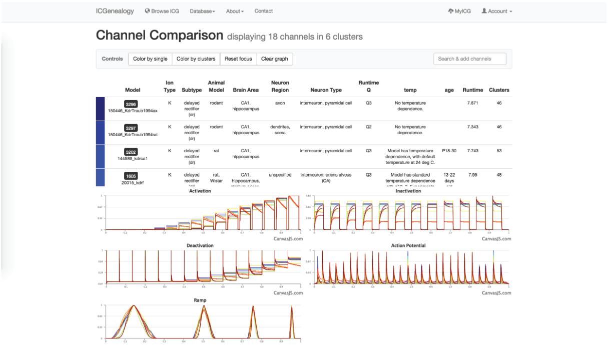

Figure 37: Example 3: Comparison of models from , with all dr-labeled models and not specified-labeled models selected.

there are for each one, so you go to the *XY view* and make the axes subtype and cluster (Figure 36). You see that there are seven behaviors for A-type, six behaviors for dr, two for m-type and so on. Let's say you are interested in looking at the delayed rectifier models, so you select all of these to compare (Figure 37). Just in case some *not specified* channels are also delayed-rectifier-like, you select these as well, and go to the compare screen. You can now see all of the models available for your particular study and compare them side-by-side according to both metadata and kinetics.
