# Supplementary material for: Mapping the function of neuronal ion channels in model and experiment
Source: eLife. 2017 Mar 6;6:e22152. doi: 10.7554/eLife.22152 (PMC5340531; doi:10.7554/eLife.22152)
Supplement: Supplementary file 2. — This file lists all model files contained in the database and used for analysis here, as well as the accompanying reference publication for each one. DOI: http://dx.doi.org/10.7554/eLife.22152.021 [file elife-22152-supp2.pdf]

Supplementary References - List of models and reference details of all database entries

[Hemond et al., 2008] 101629_KahpM95.mod (1), 101629_cacumm.mod (2), 101629_cagk.mod (3), 101629_cal2.mod (4), 101629_can2.mod (5), 101629_cat.mod (6), 101629_distr.mod (7), 101629_h.mod (8), 101629_kaprox.mod (9), 101629_kd.mod (10), 101629_kdrca1.mod (11), 101629_km.mod (12), 101629_na3n.mod (13), 101629_naxn.mod (14)

[Lawrence et al., 2006] 102288_ICaL.mod (15), 102288_ICaT.mod (16), 102288_IKCa.mod (17), 102288_IKa.mod (18), 102288_IMminret.mod (19), 102288_IMmintau.mod (20), 102288_Ih.mod (21), 102288_Ikdrf.mod (22), 102288_Ikdrfaxon.mod (23), 102288_Ikdrs.mod (24), 102288_Ikdrsaxon.m (25), 102288_Ikleakaxon.mod (26), 102288_Ikleaksd.mod (27), 102288_Ipassaxon.mod (28), 102288_Ipasssd.mod (29), 102288_Naaxon.mod (30), 102288_Nadend.mod (31), 102288_Nasoma.mod (32), 102288_SIN.mod (33), 102288_cad.mod (34)

[Mercer et al., 2007] 105385_bk_gp.mod (35), 105385_ca_gp.mod (36), 105385_cal_gp.mod (37), 105385_cap_gp.mod (38), 105385_hcn12_gp.mod (39), 105385_hcn2_gp.mod (40), 105385_iramp.mod (41), 105385_kcnq_gp.mod (42), 105385_kir2_gp.mod (43), 105385_kleak_gp.mod (44), 105385_kv1_gp.mod (45), 105385_kv2_gp.mod (46), 105385_kv3_gp.mod (47), 105385_kv4_gp.mod (48), 105385_leak_gp.mod (49), 105385_na1_gp.mod (50), 105385_na6_gp.mod (51), 105385_pas_gp.mod (52), 105385_sk_gp.mod (53)

[Lytton and Omurtag, 2007] 105507_intf_.mod (54), 105507_misc.mod (55), 105507_nstim.mod (56), 105507_stats.mod (57), 105507_vecst.mod (58)

[Cassara and Maraviglia, 2008] 106551_distr.mod (59), 106551_h.mod (60), 106551_kadist.mod (61), 106551_kaprox.mod (62), 106551_kdrca1.mod (63), 106551_na3n.mod (64), 106551_naxn.mod (65), 106551_netstims.mod (66)

[Cassara et al., 2008]

[Letzkus et al., 2006] 108459_NMDA_Mg.mod (79), 108459_ca.mod (80), 108459_cad.mod (81), 108459_epsp.mod (82), 108459_h.mod (83), 108459_it2.mod (84), 108459_kaprox.mod (85), 108459_kca.mod (86), 108459_km.mod (87), 108459_kv.mod (88), 108459_na.mod (89), 108459_release_BMK.mod (90)

[Bar-Yehuda and Korngreen, 2008] 110560_calcium.mod (91), 110560_potassium.mod (92)

[Migliore et al., 2008a] 112359_IhDA.mod (94), 112359_ampasyn.mod (95), 112359_cabalan.mod (96), 112359_cachan.mod (97), 112359_capump.m (98), 112359_dop.mod (99), 112359_hh3.mod (100), 112359_kca.mod (101), 112359_leak.mod (102), 112359_nabalan.mod (103), 112359_nmdasyn.mod (104), 112359_pump.mod (105), 112359_hh3_icgK2.mod (3475)

[Shah et al., 2008] 112546_KahpM95.mod (106), 112546_cacumm.mod (107), 112546_cagk.mod (108), 112546_cal2.mod (109), 112546_can2.mod (110), 112546_cat.mod (111), 112546_h.mod (112), 112546_kadist.mod (113), 112546_kaprox.mod (114), 112546_kdrca1.mod (115), 112546_km.mod (116), 112546_na3n.mod (117), 112546_naxn.mod (118)

[Solinas et al., 2007b]

[Solinas et al., 2007a] 112685_Golgi_BK.mod (119), 112685_Golgi_CALC.mod (120), 112685_Golgi_CALC_ca2.mod (121), 112685_Golgi_Ca_HVA.m (122), 112685_Golgi_Ca_LVA.mod (123), 112685_Golgi_KA.mod (124), 112685_Golgi_KM.mod (125), 112685_Golgi_KV.mod (126), 112685_Golgi_Na.mo (127), 112685_Golgi_NaP.mod (128), 112685_Golgi_NaR.mod (129), 112685_Golgi_SK2.mod (130), 112685_Golgi_hcn1.mod (131), 112685_Golgi_hcn2.mo (132), 112685_Golgi_lkg.mod (133), 112685_Pregen.mod (134), 112685_Synapse.mod (135)

[Wolf et al., 2005] 112834_AMPA.mod (136), 112834_GABA.mod (137), 112834_NMDA.mod (138), 112834_bkkca.mod (139), 112834_caL.mod (140), 112834_caL13.mod (141), 112834_cadyn.mod (142), 112834_caldyn.mod (143), 112834_can.mod (144), 112834_caq.mod (145), 112834_car.mod (146), 112834_cat.mod (147), 112834_kaf.mod (148), 112834_kas.mod (149), 112834_kir.mod (150), 112834_krp.mod (151), 112834_naf.mod (152), 112834_nap.mod (153), 112834_skkca.mod (154), 112834_stim.mod (155)

[Somjen et al., 2008] 113446_accum.mod (156), 113446_cal2.mod (157), 113446_capump.mod (158), 113446_getconc.mod (159), 113446_hzclamp.mo (160), 113446_hzclampna.mod (161), 113446_it.mod (162), 113446_itot.mod (163), 113446_ka.mod (164), 113446_kdr.mod (165), 113446_kdrglia.mod (166), 113446_kir.mod (167), 113446_leak.mod (168), 113446_na.mod (169), 113446_nakpump.mod (170), 113446_nap.mod (171), 113446_nastim.mod (172), 113446_nax.mod (173), 113446_nmda.mod (174), 113446_sk.mod (175), 113446_xiong.mod (176)

[Jones et al., 2007] 113732_ar.mod (177), 113732_ca.mod (178), 113732_cad.mod (179), 113732_cat.mod (180), 113732_catp.mod (181), 113732_dipole.mod (182), 113732_dpresyn.mod (183), 113732_gnetstim.mod (184), 113732_h.mod (185), 113732_kca.mod (186), 113732_kdr.mod (187), 113732_km.mod (188), 113732_na.mod (189), 113732_pp_dipole.mod (190), 113732_precall.mod (191), 113732_seed.mod (192), 113732_tchannel.m (193)

[Winograd et al., 2008] 113997_APCounter2.mod (194), 113997_Cadynamics.mod (195), 113997_HH2.mod (196), 113997_ICaL.mod (197), 113997_IKM.mod (198), 113997_Ih.mod (199), 113997_ipulse3.mod (200), 113997_pasi.mod (201), 144376_APCounter2.mod (1301), 144376_Cadynamics.mod (1302), 144376_HH2.mod (1303), 144376_ICaL.mod (1304), 144376_IKM.mod (1305), 144376_Ih.mod (1306), 144376_ipulse3.mod (1307), 144376_pasi.mod (1308)

[Saraga et al., 2006] 114047_gap.mod (202), 114047_hh.mod (203)

[Rabinowitch and Segev, 2006b]

[Rabinowitch and Segev, 2006a]

[Rabinowitch and Segev, 2008] 114355_G_h.mod (204), 114355_G_kadist.mod (205), 114355_G_kaprox.mod (206), 114355_G_kdr.mod (207), 114355_G_na.mod (208), 114355_G_nax.mod (209), 114355_M_h.mod (210), 114355_M_kadist.mod (211), 114355_M_kaprox.mod (212), 114355_M_kdr.mod (213), 114355_M_na.mod (214), 114355_M_nax.mod (215), 114355_coordinates.mod (216), 114355_exp.mod (217), 114355_peakrec.mod (218), 114355_randomizer.mod (219), 114355_scexp2syn.mod (220), 114355_scinjectsyn.mod (221), 114355_spike.mod (222)

[van Elburg and van Ooyen, 2010] 114359_SynAlphaPoissson.mod (223), 114359_ca.mod (224), 114359_cad.mod (225), 114359_kca.mod (226), 114359_km.mod (227), 114359_kv.mod (228), 114359_na.mod (229)

[Kole et al., 2008] 114394_Km.mod (230), 114394_Kv1.mod (231), 114394_h.mod (232), 114394_kv.mod (233), 114394_na.mod (234), 114394_nax.mod (235)

[Migliore et al., 2007] 114665_kamt.mod (236), 114665_kdrmt.mod (237), 114665_naxn.mod (238), 114665_nmdanetOB.mod (239)

[Johnson and McIntyre, 2008] 114685_AXNODE75.mod (240), 114685_HCN.mod (241), 114685_HVA.mod (242), 114685_KDRf.mod (243), 114685_KDRs.mod (244), 114685_Na.mod (245), 114685_NaL.mod (246), 114685_PARAK75.mod (247), 114685_SK.mod (248), 114685_cacum.mod (249), 114685_gpi.mod (250), 114685_myions.mod (251), 114685_AXNODE75_icgNA2.mod (3455)

[Tseng et al., 2008] 114735_gap.mod (252)

[Royeck et al., 2008] 115356_CAR.mod (253), 115356_CAT.mod (254), 115356_CAl.mod (255), 115356_CAnpq.mod (256), 115356_Ca2Dyn.mod (257), 115356_CaDyn.mod (258), 115356_KAHP.mod (259), 115356_KCT.mod (260), 115356_NaIn.mod (261), 115356_Nap.mod (262), 115356_h.mod (263), 115356_ka.mod (264), 115356_kdr.mod (265), 115356_km.mod (266), 115356_na3.mod (267), 115356_naxmig.mod (268)

[van Elburg and van Ooyen, 2009] 115357_intfire4r.mod (269)

[David et al., 2008] 116094_GradGABAa.mod (270), 116094_cadecay.mod (271), 116094_currentGauss.mod (272), 116094_flushf.mod (273), 116094_ipscGauss.mod (274), 116094_kA.mod (275), 116094_kM.mod (276), 116094_kca.mod (277), 116094_kfasttab.mod (278), 116094_kslowtab.mod (279), 116094_lcafixed.mod (280), 116094_nafast.mod (281), 116094_nagran.mod (282), 116094_nmdanet.mod (283), 116094_shuntInhib.mod (284), 116094_stim2.mod (285)

[Badoual et al., 2006] 116096_ampacin.mod (286), 116096_ca.mod (287), 116096_cad.mod (288), 116096_cadspine.mod (289), 116096_kca.mod (290), 116096_km.mod (291), 116096_kv.mod (292), 116096_ltpltd.mod (293), 116096_na.mod (294), 116096_nmdacin.mod (295)

[Aradi and Holmes, 1999] 116740_Ca.mod (296), 116740_CadepK.mod (297)

[Grunditz et al., 2008] 116769_ampa.mod (298), 116769_cadiffus.mod (299), 116769_canmda.mod (300), 116769_car.mod (301), 116769_nmda.mod (302)

[Lytton et al., 2008b] 116830_intf.mod (303), 116830_misc.mod (304), 116830_nstim.mod (305), 116830_stats.mod (306), 116830_vecst.mod (307)

[Diwakar et al., 2009] 116835_AmpaCOD.mod (308), 116835_GRC_CA.mod (309), 116835_GRC_CALC.mod (310), 116835_GRC_GABA.mod (311), 116835_GRC_KA.mod (312), 116835_GRC_KCA.mod (313), 116835_GRC_KIR.mod (314), 116835_GRC_KM.mod (315), 116835_GRC_KV.mod (316), 116835_GRC_LKG1.mod (317), 116835_GRC_LKG2.mod (318), 116835_GRC_NA.mod (319), 116835_NmdaS.mod (320), 116835_Pregen.mod (321)

[Lytton et al., 2008a] 116838_intf_.mod (322), 116838_misc.mod (323), 116838_nstim.mod (324), 116838_stats.mod (325), 116838_vecst.mod (326)

[Zhu et al., 1999b] 116862_AMPA.mod (327), 116862_GABAA.mod (328), 116862_GABAB.mod (329), 116862_HH2.mod (330), 116862_IL.mod (331), 116862_IL3.mod (332), 116862_IT.mod (333), 116862_IT2.mod (334), 116862_Iahp.mod (335), 116862_Ican.mod (336), 116862_Ih.mod (337), 116862_NMDA.mod (338), 116862_cadecay.mod (339), 116862_clampex.mod (340), 116862_cp.mod (341), 116862_cp2.mod (342), 116862_kdr2.mod (343), 116862_kleak.mod (344), 116862_kmbg.mod (345), 116862_naf2.mod (346), 116862_nap.mod (347), 116862_ntIh.mod (348), 116862_nthh.mod (349), 116862_ntleak.mod (350), 116862_ntt.mod (351), 116862_pregencv.mod (352), 116862_vecst.mod (353)

[Zhu et al., 1999a] 116862_AMPA.mod (327), 116862_GABAA.mod (328), 116862_GABAB.mod (329), 116862_HH2.mod (330), 116862_IL.mod (331), 116862_IL3.mod (332), 116862_IT.mod (333), 116862_IT2.mod (334), 116862_Iahp.mod (335), 116862_Ican.mod (336), 116862_Ih.mod (337), 116862_NMDA.mod (338), 116862_cadecay.mod (339), 116862_clampex.mod (340), 116862_cp.mod (341), 116862_cp2.mod (342), 116862_kdr2.mod (343), 116862_kleak.mod (344), 116862_kmbg.mod (345), 116862_naf2.mod (346), 116862_nap.mod (347), 116862_ntIh.mod (348), 116862_nthh.mod (349), 116862_ntleak.mod (350), 116862_ntt.mod (351), 116862_pregencv.mod (352), 116862_vecst.mod (353)

[Borst and Haag, 1996] 116956_emdk.mod (354), 116956_emdkna.mod (355), 116956_emdna.mod (356)

[Haag et al., 1997] 116956_emdk.mod (354), 116956_emdkna.mod (355), 116956_emdna.mod (356)

[Haag et al., 1999] 116956_emdk.mod (354), 116956_emdkna.mod (355), 116956_emdna.mod (356)

[Marcelin et al., 2009] 116983_h.mod (358)

[Acker and Antic, 2009] 117207_CaT.mod (359), 117207_Cad.mod (360), 117207_IL.mod (361), 117207_PlateauConductance.mod (362), 117207_ca.mod (363), 117207_kadist.mod (364), 117207_kaprox.mod (365), 117207_kv.mod (366), 117207_na.mod (367), 117207_vmax.mod (368)

[Cruz et al., 2007] 117459_hh2.mod (369), 117459_kleak.mod (370)

[Hemond et al., 2009] 118098_distr.mod (371), 118098_hca3.mod (372)

[Kondgen et al., 2008] 118631_Isinunoisy.mod (373), 118631_Isinunoisy2.mod (374), 118631_wb.mod (375)

[Gouwens and Wilson, 2009] 118662_LeakConductance.mod (376), 118662_v_atten.mod (377)

[Miceli et al., 2009] 118986_KahpM95.mod (378), 118986_cacumm.mod (379), 118986_cat.mod (380), 118986_h.mod (381), 118986_kadist.mod (382), 118986_kaprox.mod (383), 118986_kdrca1.mod (384), 118986_kmquad.mod (385), 118986_kmtwt.mod (386), 118986_na3n.mod (387), 118986_naxn.mod (388)

[Ferrante et al., 2008] 119283_distr.mod (407), 119283_h.mod (408), 119283_kadist.mod (409), 119283_kaprox.mod (410), 119283_kdrca1.mod (411), 119283_na3n.mod (412), 119283_naxn.mod (413), 119283_netstimm.mod (414)

[Chapman et al., 1983] 120692_ionleak.mod (415), 120692_nakpump.mod (416), 120692_nakpump1.mod (417)

[Palmer and Stuart, 2009] 120798_syn_g.mod (418)

[Vazquez et al., 2009] 120910_cachdend.mod (419), 120910_gkcadend.mod (420), 120910_ikhhdend.mod (421), 120910_kadend.mod (422), 120910_leakdend.mod (423), 120910_nadend.mod (424)

[Steephen and Manchanda, 2009] 121060_AMPA.mod (425), 121060_GABA.mod (426), 121060_NMDA.mod (427), 121060_chan_BKKCa.mod (428), 121060_chan_CaKdyn.mod (429), 121060_chan_CaL12.mod (430), 121060_chan_CaL13.mod (431), 121060_chan_CaN.mod (432), 121060_chan_CaQ.mod (433), 121060_chan_CaR.mod (434), 121060_chan_CaT.mod (435), 121060_chan_Cadyn.mod (436), 121060_chan_KAs.mod (437), 121060_chan_KIR.mod (438), 121060_chan_KRP.mod (439), 121060_chan_NaF.mod (440), 121060_chan_NaP.mod (441), 121060_chan_SKKCa.mo (442), 121060_chan_inKIR.mod (443)

[Bellinger et al., 2008] 121253_Nakpump.mod (444), 121253_axnodenew2.mod (445), 121253_fastK.mod (446), 121253_kdifl.mod (447), 121253_kdifrl.mod (448), 121253_train.mod (449), 121253_axnodenew2_icgNA2.mod (3456)

[Bogaard et al., 2009] 121259_WMPas.mod (450), 121259_WMPas2.mod (451), 121259_WMPasDend.mod (452), 121259_WMPasDend2.mod (453), 121259_h.mod (454), 121259_h2.mod (455), 121259_kaprox.mod (456), 121259_kaprox2.mod (457), 121259_kdrca1.mod (458), 121259_kdrca12.mo (459), 121259_nax.mod (460), 121259_nax2.mod (461)

[Deister et al., 2009] 122329_ca_gp.mod (462), 122329_cap_gp.mod (463), 122329_hcn12_gp.mod (464), 122329_hcn2_gp.mod (465), 122329_kcnq_gp.mod (466), 122329_kv1_gp.mod (467), 122329_kv2_gp.mod (468), 122329_kv3_gp.mod (469), 122329_kv4_gp.mod (470), 122329_leak_gp.mod (471), 122329_na1_gp.mod (472), 122329_na6_gp.mod (473), 122329_sk_gp.mod (474), 122329_syn.mod (475)

[Akemann et al., 2009] 123453_Caint.mod (476), 123453_Cav2.mod (477), 123453_HCN.mod (478), 123453_KCa1.mod (479), 123453_Kv1.mod (480), 123453_Kv3.mod (481), 123453_Kv4.mod (482), 123453_Nav11.mod (483), 123453_VSFP23M3.mod (484), 123453_VSFP31M3.mod (485), 123453_leak.mod (486)

[Pospischil et al., 2008] 123623_HH_traub.mod (487), 123623_IL_gutnick.mod (488), 123623_IM_cortex.mod (489), 123623_IT_huguenard.mod (490), 123623_cadecay_destexhe.mod (491)

[Cutsuridis et al., 2010] 123815_ANsyn.mod (492), 123815_IA.mod (493), 123815_Ih.mod (494), 123815_Kaxon.mod (495), 123815_Kdend.mod (496), 123815_Ksoma.mod (497), 123815_LcaMig.mod (498), 123815_Naaxon.mod (499), 123815_Nadend.mod (500), 123815_Nasoma.mod (501), 123815_STDPE2Syn.mod (502), 123815_bgka.mod (503), 123815_burststim2.mod (504), 123815_cad.mod (505), 123815_cagk.mod (506), 123815_cal.mod (507), 123815_calH.mod (508), 123815_car.mod (509), 123815_cat.mod (510), 123815_ccanl.mod (511), 123815_gskch.mod (512), 123815_h.mod (513), 123815_hNa.mod (514), 123815_hha2.mod (515), 123815_hha_old.mod (516), 123815_ichan2.mod (517), 123815_kad.mod (518), 123815_kap.mod (519), 123815_kca.mod (520), 123815_km.mod (521), 123815_my_exp2syn.mod (522), 123815_nca.mod (523), 123815_nmda.mod (524), 123815_regn_stim.mod (525), 123815_somacar.mod (526), 123815_ichan2_icgK2.mod (3476)

[Hu et al., 2009] 123897_ca.mod (527), 123897_cad.mod (528), 123897_gabaa.mod (529), 123897_kca.mod (530), 123897_km.mod (531), 123897_kv.mod (532), 123897_na.mod (533), 123897_na12.mod (534), 123897_na16.mod (535)

[Wimmer et al., 2010] 123927_CAR.mod (536), 123927_CAT.mod (537), 123927_CAl.mod (538), 123927_CAnpq.mod (539), 123927_Ca2Dyn.mod (540), 123927_CaDyn.mod (541), 123927_KAHP.mod (542), 123927_KCT.mod (543), 123927_KM.mod (544), 123927_Nap.mod (545), 123927_NapIn.mod (546), 123927_h.mod (547), 123927_ka.mod (548), 123927_kdr.mod (549), 123927_kleck.mod (550), 123927_kslow.mod (551), 123927_na3.mod (552), 123927_naxmig.mod (553)

[Larkum et al., 2009] 124043_Kdr.mod (554), 124043_SlowCa.mod (555), 124043_ampa.mod (556), 124043_cad2.mod (557), 124043_glutamate.mod (558), 124043_h.mod (559), 124043_h2.mod (560), 124043_hh3.mod (561), 124043_ih.mod (562), 124043_it2.mod (563), 124043_kap.mod (564), 124043_kca.mod (565), 124043_kdf.mod (566), 124043_kdr2.mod (567), 124043_km.mod (568), 124043_hh3_icgK2.mod (3477)

[Stehle et al., 1990] 124063_A2hh_k.mod (569), 124063_A2hh_na.mod (570), 124063_Bip_Ca.mod (571), 124063_Bip_Cad.mod (572), 124063_Bip_Ka.mod (573), 124063_Bip_Kv.mod (574), 124063_Bip_ih.mod (575), 124063_Cone_CPR.mod (576), 124063_Cone_Kv.mod (577), 124063_Cone_ih.mod (578), 124063_Ganglion_hh.mod (579), 124063_Rod_Ca.mod (580), 124063_Rod_Cad.mod (581), 124063_Rod_Clca.mod (582), 124063_Rod_Kca.mod (583), 124063_Rod_Kv.mod (584), 124063_Rod_Kx.mod (585), 124063_Rod_ih.mod (586), 124063_Rod_leak.mod (587), 124063_gap.mod (588), 124063_syn_bip_gan.mod (589), 124063_syn_rod_bip.mod (590), 124063_Ganglion_hh_icgK2.mod (3478)

[Ferrante et al., 2009] 124291_fna.mod (591), 124291_ichan2.mod (592), 124291_kaprox.mod (593), 124291_kdr.mod (594), 124291_kdrca1.mod (595), 124291_na3n.mod (596), 124291_nmdanetGR.mod (597), 124291_ichan2_icgK2.mod (3449)

[Nevian et al., 2007] 124394_ampa_syn.mod (598), 124394_double_synapse.mod (599), 124394_hh3.mod (600), 124394_nmda_syn.mod (601)

[Santhakumar et al., 2005] 51781_CaBK.mod (2068), 51781_Gfluct2.mod (2069), 51781_LcaMig.mod (2070), 51781_bgka.mod (2071), 51781_ccanl.mod (2072), 51781_gskch.mod (2073), 51781_hyperde3.mod (2074), 51781_ichan2.mod (2075), 51781_nca.mod (2076), 51781_tca.mod (2077), 97917_CaBK.mod (2596), 97917_Gfluct2.mod (2597), 97917_LcaMig.mod (2598), 97917_alphasyndiffeq.mod (2599), 97917_alphasynkin.mod (2600), 97917_alphasynkint.mod (2601), 97917_ampa.mod (2602), 97917_ar.mod (2603), 97917_bgka.mod (2604), 97917_cad.mod (2605), 97917_cal.mod (2606), 97917_cat.mod (2607), 97917_cat_a.mod (2608), 97917_ccanl.mod (2609), 97917_gabaa.mod (2610), 97917_gskch.mod (2611), 97917_hyperde3.mod (2612), 97917_ichan2.mod (2613), 97917_iclamp_const.mod (2614), 97917_k2.mod (2615), 97917_kahp.mod (2618), 97917_kahp_deeppyr.mod (2619), 97917_kahp_slower.mod (2620), 97917_kc.mod (2621), 97917_kc_fast.mod (2622), 97917_kdr.mod (2623), 97917_kdr_fs.mod (2624), 97917_km.mod (2625), 97917_naf.mod (2626), 97917_naf2.mod (2627), 97917_naf_tcr.mod (2628), 97917_nap.mod (2629), 97917_napf.mod (2630), 97917_napf_spinstell.mod (2631), 97917_napf_tcr.mod (2632), 97917_nca.mod (2633), 97917_par_ggap.mod (2634), 97917_pulsesyn.mod (2635), 97917_rampsyn.mod (2636), 97917_rand.mod (2637), 97917_ri.mod (2638), 97917_tca.mod (2639), 97917_traub_nmda.mod (2640), 97917_ichan2_icgK2.mod (3454), 51781_hyperde3_icgIH2.mod (3471), 97917_hyperde3_icgIH2.mod (3472), 51781_ichan2_icgK2.mod (3488)

[Dyhrfjeld-Johnsen et al., 2007]

[Morgan and Soltesz, 2008]

[Morgan et al., 2007] 124513_CaBK.mod (602), 124513_Gfluct2.mod (603), 124513_LcaMig.mod (604), 124513_bgka.mod (605), 124513_ccanl.mod (606), 124513_gskch.mod (607), 124513_hyperde3.mod (608), 124513_ichan2.mod (609), 124513_inhsyn.mod (610), 124513_nca.mod (611), 124513_ppsyn.mod (612), 124513_tca.mod (613), 124513_ichan2_icgK2.mod (3450), 124513_hyperde3_icgIH2.mod (3463)

[Uebachs et al., 2010] 125152_CAR.mod (614), 125152_CAT.mod (615), 125152_CAl.mod (616), 125152_CAnpq.mod (617), 125152_Ca2Dyn.mod (618), 125152_CaDyn.mod (619), 125152_KAHP.mod (620), 125152_KCT.mod (621), 125152_KM.mod (622), 125152_Nap.mod (623), 125152_NapIn.mod (624), 125152_h.mod (625), 125152_ka.mod (626), 125152_kdr.mod (627), 125152_kleck.mod (628), 125152_kslow.mod (629), 125152_na3.mod (630), 125152_naxmig.mod (631)

[Lee et al., 2003] 125378_ca12dZUy.mod (632)

[Diba et al., 2006] 125385_RNG.mod (633), 125385_cad2.mod (634), 125385_child.mod (635), 125385_childa.mod (636), 125385_epsp.mod (637), 125385_myvmax.mod (638), 125385_sca.mod (639), 125385_sit2.mod (640), 125385_skaprox.mod (641), 125385_skca.mod (642), 125385_skm.mod (643), 125385_skv.mod (644), 125385_sna.mod (645), 125385_snap.mod (646)

[Carvalho and Buonomano, 2009] 125689_EPlasSom.mod (647), 125689_EPlasSyn.mod (648), 125689_EPlasSyntoInh.mod (649), 125689_EtoISom (650), 125689_ExIAF.mod (651), 125689_INPUT.mod (652), 125689_IPlasSom.mod (653), 125689_IPlasSyn.mod (654), 125689_InhIAF.mod (655), 125689_noise.mod (656)

[Fink et al., 2000] 125745_bkr.mod (657), 125745_cdp.mod (658), 125745_ip3cum.mod (659)

[Lytton, 1997] 12631_AMPA.mod (669), 12631_GABAA.mod (670), 12631_GABAB1.mod (671), 12631_GABALOW.mod (672), 12631_HH_traub.mo (673), 12631_IAHP_destexhe.mod (674), 12631_ICAN_destexhe.mod (675), 12631_ICAN_voltdep.mod (676), 12631_IT2_huguenard.mod (677), 12631_IT_wang.mod (678), 12631_Ih_old.mod (679), 12631_NMDA.mod (680), 12631_calciumpump_destexhe.mod (681)

[Negroni and Lascano, 1996] 126467_Cont.mod (687)

[Brown et al., 2011] 126637_CaE.mod (688), 126637_CaEcvode.mod (689), 126637_CaP2.mod (690), 126637_CaP2cvode.mod (691), 126637_CaT.mod (692), 126637_CaTcvode.mod (693), 126637_CalciumP.mod (694), 126637_K23.mod (695), 126637_K23cvode.mod (696), 126637_KA.mod (697), 126637_KAcvode.mod (698), 126637_KC3.mod (699), 126637_KC3cvode.mod (700), 126637_KD.mod (701), 126637_KDcvode.mo (702), 126637_KM.mod (703), 126637_KMcvode.mod (704), 126637_Kh.mod (705), 126637_Khcvode.mod (706), 126637_Khh.mod (707), 126637_Khhcvode.mod (708), 126637_Leak.mod (709), 126637_NaF.mod (710), 126637_NaFcvode.mod (711), 126637_NaP.mod (712), 126637_NaPcvode (713)

[Spiga et al., 2010] 126640_kaf.mod (714), 126640_kas.mod (715), 126640_krp.mod (716), 126640_naf.mod (717), 126640_netstimd.mod (718)

[Ascoli et al., 2010] 126776_h.mod (719), 126776_kadist.mod (720), 126776_kaprox.mod (721), 126776_kdrca1.mod (722), 126776_na3n.mod (723), 126776_naxn.mod (724)

[Safiulina et al., 2010] 126814_KahpM95.mod (725), 126814_cacumm.mod (726), 126814_cagk.mod (727), 126814_cal2.mod (728), 126814_can2.mod (729), 126814_cat.mod (730), 126814_h.mod (731), 126814_kaprox.mod (732), 126814_kdrca1.mod (733), 126814_km.mod (734), 126814_na3n.mod (735)

[Botta et al., 2010] 127021_Golgi_BK.mod (736), 127021_Golgi_CALC.mod (737), 127021_Golgi_CALC_ca2.mod (738), 127021_Golgi_Ca_HVA.mod (739), 127021_Golgi_Ca_LVA.mod (740), 127021_Golgi_KA.mod (741), 127021_Golgi_KM.mod (742), 127021_Golgi_KV.mod (743), 127021_Golgi_Na.mo (744), 127021_Golgi_NaP.mod (745), 127021_Golgi_NaR.mod (746), 127021_Golgi_SK2.mod (747), 127021_Golgi_hcn1.mod (748), 127021_Golgi_hcn2.mo (749), 127021_Golgi_lkg.mod (750), 127021_K_conc.mod (751), 127021_Na_conc.mod (752), 127021_Pregen.mod (753), 127021_Synapse.mod (754), 127021_pump.mod (755)

[Hahn and McIntyre, 2010] 127388_ampap.mod (756), 127388_dbsStim.mod (757), 127388_gabaap.mod (758), 127388_myions.mod (759), 127388_pGPeA.mod (760), 127388_pSTN.mod (761), 127388_scopRandom.mod (762), 127388_twoStateGen.mod (763), 127388_pSTN_icgK2.mod (3479), 127388_pSTN_icgCA2.mod (3494)

[Kuznetsova et al., 2010] 127507_cabal.mod (764), 127507_nabalan.mod (765), 127507_newcachan.mod (766), 127507_newhh3.mod (767), 127507_newkca.mod (768), 127507_newleak.mod (769), 127507_pump.mod (770), 127507_stim.mod (771), 127507_newhh3_icgK2.mod (3480)

[Linaro et al., 2011] 127992_HHcn.mod (772), 127992_HHcnf.mod (773), 127992_HHmicro.mod (774)

[Migliore et al., 2010] 127995_ThreshDetect.mod (775), 127995_ampanmda.mod (776), 127995_fi.mod (777), 127995_kamt.mod (778), 127995_kdrmt.mod (779), 127995_naxn.mod (780)

[Schmidt-Hieber and Bischofberger, 2010] 128079_HHrates.mod (781), 128079_KIn.mod (782), 128079_hhmfb.mod (783), 128079_na8st.mod (784), 128079_nakole.mod (785), 128079_naxkole.mod (786), 128079_spines.mod (787)

[Nieus et al., 2006] 128446_Ampa.mod (788), 128446_Nmda.mod (789), 128446_pregen2.mod (790)

[Aguiar et al., 2010] 128559_AMPA_DynSyn.mod (791), 128559_CaIntraCellDyn.mod (792), 128559_GABAa_DynSyn.mod (793), 128559_GABAb_ (794), 128559_HH2.mod (795), 128559_NK1_DynSyn.mod (796), 128559_NMDA_DynSyn.mod (797), 128559_iCaAN.mod (798), 128559_iCaL.mod (799), 128559_iKCa.mod (800), 128559_iNaP.mod (801), 128559_mGluR_DynSyn.mod (802)

[Welday et al., 2011] 129067_Nap.mod (803), 129067_ampa.mod (804), 129067_vecevent.mod (805)

[Shu et al., 2007a]

[Shu et al., 2006] 135787_Gfluct.mod (806), 135787_NMDA_Mg.mod (807), 135787_ampa5.mod (808), 135787_ca.mod (809), 135787_caL3d.mod (810), 135787_cad.mod (811), 135787_capump.mod (812), 135787_gabaa5.mod (813), 135787_ia.mod (814), 135787_iahp.mod (815), 135787_iahp2.mod (816), 135787_ih.mod (817), 135787_im.mod (818), 135787_kca.mod (819), 135787_km.mod (820), 135787_kv.mod (821), 135787_na.mod (822), 135787_nmda5.mod (823), 135787_release.mod (824)

[Alle et al., 2009] 135838_GCa.mod (825), 135838_GK.mod (826), 135838_GNa.mod (827), 135838_peak.mod (828)

[McCormick et al., 2007]

[Yu et al., 2008] 135839_Gfluct.mod (829), 135839_NMDA_Mg.mod (830), 135839_ca.mod (831), 135839_caL3d.mod (832), 135839_cad.mod (833), 135839_capump.mod (834), 135839_gabaa5.mod (835), 135839_ia.mod (836), 135839_iahp.mod (837), 135839_iahp2.mod (838), 135839_ih.mod (839), 135839_im.mod (840), 135839_kca.mod (841), 135839_km.mod (842), 135839_kv.mod (843), 135839_na.mod (844), 135839_nmda5.mod (845), 135839_release.mod (846)

[Shu et al., 2007b] 135898_Gfluct.mod (847), 135898_h.mod (848), 135898_kd.mod (849), 135898_kv.mod (850), 135898_na.mod (851)

[Stacey et al., 2009] 135902_CA1ih.mod (852), 135902_CA1ika.mod (853), 135902_CA1ikdr.mod (854), 135902_CA1ina.mod (855), 135902_Edrive.mod (856), 135902_Exp2SynAMPA.mod (857), 135902_Exp2SynAMPApre.mod (858), 135902_Exp2SynNMDA.mod (859), 135902_Exp2SynNMDAperm.mod (860), 135902_Exp2SynNMDApre.mod (861), 135902_caolmw.mod (862), 135902_capr.mod (863), 135902_fvpre.mod (864), 135902_gap.mod (865), 135902_halfgap.mod (866), 135902_icaolmw.mod (867), 135902_icapr.mod (868), 135902_iholmkop.mod (869), 135902_iholmw.mod (870), 135902_ihpyrkop.mod (871), 135902_kahppr.mod (872), 135902_kaolmkop.mod (873), 135902_kapyrkop.mod (874), 135902_kcaolmw.mod (875), 135902_kcpr.mod (876), 135902_kdrbwb.mod (877), 135902_kdrca1.mod (878), 135902_kdrolmkop.mod (879), 135902_kdrpr.mod (880), 135902_kdrpyrkop.mod (881), 135902_na3n.mod (882), 135902_nafbwb.mod (883), 135902_nafolmkop.mod (884), 135902_nafpr.mod (885), 135902_nafpyrkop.mod (886), 135902_par_ggap.mod (887), 135902_CA1ika_icgK2.mod (3459)

[Stacey et al., 2011] 135903_CA1ih.mod (888), 135903_CA1ika.mod (889), 135903_CA1ikdr.mod (890), 135903_CA1ina.mod (891), 135903_Edrive.mod (892), 135903_Exp2SynAMPA.mod (893), 135903_Exp2SynAMPApre.mod (894), 135903_Exp2SynNMDA.mod (895), 135903_Exp2SynNMDAperm.mod (896), 135903_Exp2SynNMDApre.mod (897), 135903_caolmw.mod (898), 135903_capr.mod (899), 135903_fvpre.mod (900), 135903_gap.mod (901), 135903_halfgap.mod (902), 135903_icaolmw.mod (903), 135903_icapr.mod (904), 135903_iholmkop.mod (905), 135903_iholmw.mod (906), 135903_ihpyrkop.mod (907), 135903_kahppr.mod (908), 135903_kaolmkop.mod (909), 135903_kapyrkop.mod (910), 135903_kcaolmw.mod (911), 135903_kcpr.mod (912), 135903_kdrbwb.mod (913), 135903_kdrca1.mod (914), 135903_kdrolmkop.mod (915), 135903_kdrpr.mod (916), 135903_kdrpyrkop.mod (917), 135903_na3n.mod (918), 135903_nafbwb.mod (919), 135903_nafolmkop.mod (920), 135903_nafpr.mod (921), 135903_nafpyrkop.mod (922), 135903_par_ggap.mod (923), 135903_CA1ika_icgK2.mod (3460)

[Djurisic et al., 2008] 136026_kd.mod (924), 136026_monx.mod (925), 136026_na.mod (926)

[Neymotin et al., 2011a] 136095_A.mod (927), 136095_AMPA.mod (928), 136095_AMPAr.mod (929), 136095_GABAa.mod (930), 136095_GABAar (931), 136095_GABAb.mod (932), 136095_GABAbr.mod (933), 136095_H.mod (934), 136095_IL.mod (935), 136095_IL3.mod (936), 136095_Iahp.mod (937), 136095_Ican.mod (938), 136095_NMDA.mod (939), 136095_NMDAr.mod (940), 136095_cp.mod (941), 136095_cp2.mod (942), 136095_kdr2.mod (943), 136095_kmbg.mod (944), 136095_naf2.mod (945), 136095_nap.mod (946), 136095_ntIh.mod (947), 136095_nthh.mod (948), 136095_ntt.mod (949)

[Katona et al., 2011] 136176_ampa_syn.mod (950), 136176_double_synapse.mod (951), 136176_hh3.mod (952), 136176_nmda_syn.mod (953)

[Munro and Kopell, 2012] 136309_extrema.mod (954), 136309_gap.mod (955), 136309_k2_Traub.mod (956), 136309_ka_Traub.mod (957), 136309_kdr_Jonas.mod (958), 136309_kdr_Traub.mod (959), 136309_kdr_Yu.mod (960), 136309_naf_Jonas.mod (961), 136309_naf_Traub.mod (962), 136309_naf_Yu.mod (963), 136309_pas_basket.mod (964), 136309_pas_chand.mod (965)

[Bedard et al., 2004] 136310_ImpedanceFM.mod (966)

[Fleidervish et al., 2010] 136715_Kv1.mod (967), 136715_hh_Ca_scaled.mod (968), 136715_hh_Cp_scaled.mod (969), 136715_hh_Cs_scaled.mod (970), 136715_kv.mod (971), 136715_nadifl.mod (972)

[Jones et al., 2009] 136803_ar.mod (973), 136803_ca.mod (974), 136803_cad.mod (975), 136803_cat.mod (976), 136803_dipole.mod (977), 136803_kca.mod (978), 136803_km.mod (979), 136803_pp_dipole.mod (980)

[Baker et al., 2011] 137259_KahpM95.mod (981), 137259_cacumm.mod (982), 137259_cagk.mod (983), 137259_cal2.mod (984), 137259_can2.mod (985), 137259_cat.mod (986), 137259_exp2nmdar.mod (987), 137259_h.mod (988), 137259_kadist.mod (989), 137259_kaprox.mod (990), 137259_kd.mod (991), 137259_kdrca1.mod (992), 137259_km.mod (993), 137259_na3n.mod (994)

[Hines et al., 2011] 137845_invlfire.mod (995)

[Gurkiewicz et al., 2011] 138082_FVNaG3e.mod (996), 138082_WTNaG3e.mod (997)

[Migliore et al., 2011] 138205_Gfluct.mod (998), 138205_distr.mod (999), 138205_h.mod (1000), 138205_kadist.mod (1001), 138205_kaprox.mod (1002), 138205_kdrca1.mod (1003), 138205_na3n.mod (1004), 138205_naxn.mod (1005), 138205_netstims.mod (1006)

[Ostroumov, 2007] 138273_IKaSM.mod (1007), 138273_IKdrSM.mod (1008), 138273_INaSM.mod (1009)

[Pashut et al., 2011] 138321_SlowCa.mod (1010), 138321_cad2.mod (1011), 138321_child.mod (1012), 138321_childa.mod (1013), 138321_epsp.mod (1014), 138321_it2.mod (1015), 138321_kaprox.mod (1016), 138321_kca.mod (1017), 138321_km.mod (1018), 138321_kv.mod (1019), 138321_na.mod (1020), 138321_xtra.mod (1021), 138321_xtra_v2.mod (1022)

[Neymotin et al., 2011c] 138379_intf6_.mod (1023), 138379_misc.mod (1024), 138379_nstim.mod (1025), 138379_stats.mod (1026), 138379_vecst.mod (1027)

[Anwar et al., 2012] 138382_CALC.mod (1028), 138382_CALC_DP.mod (1029), 138382_CALC_ca2_DP.mod (1030), 138382_CaT3_1.mod (1031), 138382_CaT3_1_DP.mod (1032), 138382_SK2.mod (1033), 138382_SK2_DP.mod (1034), 138382_cdp3.mod (1035), 138382_cdp5.mod (1036), 138382_leak.mod (1037), 138382_mslo.mod (1038), 138382_mslo_DP.mod (1039), 138382_newCaP.mod (1040), 138382_newCaP_DP.mod (1041)

[Poleg-Polsky and Diamond, 2011] 139150_glutamate.mod (1042), 139150_hh3.mod (1043), 139150_inhib.mod (1044), 139150_linsyn.mod (1045), 139150_hh3_icgK2.mod (3481)

[Fietkiewicz et al., 2011] 139418_ISK.mod (1046), 139418_capump.mod (1047), 139418_cax.mod (1048), 139418_nkpump.mod (1049), 139418_potassiumcontrol.mod (1050)

[Neymotin et al., 2011b] 139421_CA1ih.mod (1051), 139421_CA1ika.mod (1052), 139421_CA1ikdr.mod (1053), 139421_CA1ina.mod (1054), 139421_MyExp2Syn.mod (1055), 139421_MyExp2SynAlpha.mod (1056), 139421_MyExp2SynBB.mod (1057), 139421_MyExp2SynNMDA.mod (1058), 139421_MyExp2SynNMDABB.mod (1059), 139421_caolmw.mod (1060), 139421_capr.mod (1061), 139421_icaolmw.mod (1062), 139421_icapr.mod (1063), 139421_iholmkop.mod (1064), 139421_iholmw.mod (1065), 139421_ihpyrkop.mod (1066), 139421_kahppr.mod (1067), 139421_kaolmkop.mod (1068), 139421_kapyrkop.mod (1069), 139421_kcaolmw.mod (1070), 139421_kcpr.mod (1071), 139421_kdrbwb.mod (1072), 139421_kdrolmkop.mod (1073), 139421_kdrpr.mod (1074), 139421_kdrpyrkop.mod (1075), 139421_misc.mod (1076), 139421_nafbwb.mod (1077), 139421_nafolmkop.mod (1078), 139421_nafpr.mod (1079), 139421_nafpyrkop.mod (1080), 139421_stats.mod (1081), 139421_vecst.mod (1082), 139421_wrap.mod (1083), 139421_CA1ika_icgK2.mod (3461)

[Hay et al., 2011] 139653_CaDynamics_E2.mod (1084), 139653_Ca_HVA.mod (1085), 139653_Ca_LVAst.mod (1086), 139653_Ih.mod (1087), 139653_Im.mod (1088), 139653_K_Pst.mod (1089), 139653_K_Tst.mod (1090), 139653_NaTa_t.mod (1091), 139653_NaTs2_t.mod (1092), 139653_Nap_Et2.mod (1093), 139653_SK_E2.mod (1094), 139653_SKv3_1.mod (1095), 139653_epsp.mod (1096)

[Rossert et al., 2011] 139654_fh.mod (1097), 139654_kca.mod (1098), 139654_klt.mod (1099), 139654_kml.mod (1100), 139654_leak.mod (1101)

[Simoes de Souza and De Schutter, 2011] 139656_GRC_CA.mod (1102), 139656_GRC_CALC.mod (1103), 139656_GRC_KA.mod (1104), 139656_GRC_KCA.mod (1105), 139656_GRC_KIR.mod (1106), 139656_GRC_KM.mod (1107), 139656_GRC_KV.mod (1108), 139656_GRC_LKG[illegible] (1109), 139656_GRC_LKG2.mod (1110), 139656_GRC_NA.mod (1111), 139656_Golgi_BK.mod (1112), 139656_Golgi_CALC.mod (1113), 139656_Golgi_CALC_ca2.mod (1114), 139656_Golgi_Ca_HVA.mod (1115), 139656_Golgi_Ca_LVA.mod (1116), 139656_Golgi_KA.mod (1117), 139656_Golgi_KM.mod (1118), 139656_Golgi_KV.mod (1119), 139656_Golgi_Na.mod (1120), 139656_Golgi_NaP.mod (1121), 139656_Golgi_NaR.mod (1122), 139656_Golgi_SK2.mod (1123), 139656_Golgi_hcn1.mod (1124), 139656_Golgi_hcn2.mod (1125), 139656_Golgi_lkg.mod (1126), 139656_K_conc.m[illegible] (1127), 139656_Na_conc.mod (1128), 139656_gap.mod (1129)

[Kopp-Scheinpflug et al., 2011] 139657_ht.mod (1130), 139657_lt.mod (1131), 139657_lva.mod (1132), 139657_mhh_Gfluct.mod (1133), 139657_netstims.mod (1134), 139657_sjg_ih.mod (1135), 139657_sjg_na.mod (1136), 139657_trigstim.mod (1137)

[Diwakar et al., 2011] 139883_AmpaCOD.mod (1138), 139883_GRC_CA.mod (1139), 139883_GRC_CALC.mod (1140), 139883_GRC_GABA.mod (1141), 139883_GRC_KA.mod (1142), 139883_GRC_KCA.mod (1143), 139883_GRC_KIR.mod (1144), 139883_GRC_KM.mod (1145), 139883_GRC_KV.m (1146), 139883_GRC_LKG1.mod (1147), 139883_GRC_LKG2.mod (1148), 139883_GRC_NA.mod (1149), 139883_NmdaS.mod (1150), 139883_Pregen.mo (1151)

[Kovalsky et al., 2009] 140038_inter.mod (1152), 140038_kdr7.mod (1153), 140038_late2.mod (1154), 140038_shift_hh_6.mod (1155)

[Halnes et al., 2011] 140249_Cad.mod (1156), 140249_HH_traub.mod (1157), 140249_Ican.mod (1158), 140249_iahp.mod (1159), 140249_iar.mod (1160), 140249_ical.mod (1161), 140249_it2.mod (1162)

[Masurkar and Chen, 2011a] 140462_Ikt2m2h.mod (1167)

[Masurkar and Chen, 2011b] 140462_ICat1m3h.mod (1163), 140462_ICat2.mod (1164), 140462_IKt1m4h.mod (1165), 140462_IKt3.mod (1166), 140462_ipulse2.mod (1168), 140462_nmitral.mod (1169)

[Jaffe et al., 2011] 140471_Ca.mod (1170), 140471_aabBK.mod (1171)

[Branco et al., 2010]

[Branco and Hausser, 2011] 140828_CaT.mod (1172), 140828_HH2.mod (1173), 140828_NMDA_Mg_T.mod (1174), 140828_ar.mod (1175), 140828_ca.mod (1176), 140828_caL3d.mod (1177), 140828_cad.mod (1178), 140828_h.mod (1179), 140828_inwardrect.mod (1180), 140828_kca.mod (1181), 140828_kir.mod (1182), 140828_km.mod (1183), 140828_kv.mod (1184), 140828_na.mod (1185), 140828_release_BMK.mod (1186)

[Orio and Soudry, 2012] 141272_hh2CW.mod (1192), 141272_hh2F.mod (1193), 141272_hh58CW.mod (1194), 141272_hh58F1.mod (1195), 141272_hh58F1ss.mod (1196), 141272_hhdet.mod (1197)

[Vierling-Claassen et al., 2010] 141273_Gfluct.mod (1198), 141273_ar.mod (1199), 141273_ca.mod (1200), 141273_cad.mod (1201), 141273_cat.mod (1202), 141273_fdsexp2syn.mod (1203), 141273_gnetstim.mod (1204), 141273_kca.mod (1205), 141273_km.mod (1206), 141273_kv.mod (1207), 141273_na_2.mod (1208)

[Kerr et al., 2012] 141505_infot.mod (1209), 141505_intf6_.mod (1210), 141505_intfsw.mod (1211), 141505_misc.mod (1212), 141505_nstim.mod (1213), 141505_staley.mod (1214), 141505_stats.mod (1215), 141505_vecst.mod (1216)

[Fujita et al., 2012] 143100_CaH.mod (1217), 143100_HCN.mod (1218), 143100_KCNQ.mod (1219), 143100_Kv2.mod (1220), 143100_Kv3.mod (1221), 143100_Kv4f.mod (1222), 143100_Kv4s.mod (1223), 143100_NaF.mod (1224), 143100_NaP.mod (1225), 143100_SK.mod (1226), 143100_calcium.mod (1227), 143100_leak.mod (1228)

[Zhou and Colburn, 2010] 143114_Alpha.mod (1229), 143114_currentGauss.mod (1230), 143114_refrac_abs.mod (1231), 143114_refrac_rel.mod (1232), 143114_vecevent.mod (1233)

[Foust et al., 2011] 143442_kd.mod (1234), 143442_kv.mod (1235), 143442_na.mod (1236)

[Casale and McCormick, 2011] 143635_kht.mod (1237), 143635_mhw.mod (1238), 143635_na.mod (1239)

[Powers et al., 2012] 143671_Gfluctdv.mod (1240), 143671_L_Ca.mod (1241), 143671_ghchan.mod (1242), 143671_kca2.mod (1243), 143671_kdrRL.mod (1244), 143671_mAHP.mod (1245), 143671_na3rp.mod (1246), 143671_naps.mod (1247), 143671_synss.mod (1248)

[Bianchi et al., 2012] 143719_cad.mod (1249), 143719_cagk.mod (1250), 143719_cal.mod (1251), 143719_calH.mod (1252), 143719_car.mod (1253), 143719_cat.mod (1254), 143719_d3.mod (1255), 143719_h.mod (1256), 143719_kadist.mod (1257), 143719_kaprox.mod (1258), 143719_kca.mod (1259), 143719_kdr.mod (1260), 143719_km.mod (1261), 143719_na3.mod (1262), 143719_na3dend.mod (1263), 143719_na3notrunk.mod (1264), 143719_nap.mod (1265), 143719_nax.mod (1266), 143719_somacar.mod (1267)

[Jones and Gabbiani, 2012a] 144007_LGMD_Ca.mod (1268), 144007_LGMD_CaL.mod (1269), 144007_LGMD_HH_Kdr_sf35.mod (1270), 144007_LGMD_HH_Na_sf35.mod (1271), 144007_LGMD_KCa.mod (1272)

[Jones and Gabbiani, 2012b] 144007_LGMD_Ca.mod (1268), 144007_LGMD_CaL.mod (1269), 144007_LGMD_HH_Kdr_sf35.mod (1270), 144007_LGMD_HH_Na_sf35.mod (1271), 144007_LGMD_KCa.mod (1272)

[McTavish et al., 2012] 144054_ThreshDetect.mod (1273), 144054_ampanmda.mod (1274), 144054_fi.mod (1275), 144054_kamt.mod (1276), 144054_kdrmt.mod (1277), 144054_naxn.mod (1278)

[Sidiropoulou and Poirazi, 2012] 144089_NMDA.mod (1279), 144089_ampa.mod (1280), 144089_cad.mod (1281), 144089_cal.mod (1282), 144089_can.mod (1283), 144089_car.mod (1284), 144089_cat.mod (1285), 144089_d3.mod (1286), 144089_gabaa.mod (1287), 144089_gabab.mod (1288), 144089_h.mod (1289), 144089_ican.mod (1290), 144089_kadist.mod (1291), 144089_kca.mod (1292), 144089_kct.mod (1293), 144089_kd.mod (1294), 144089_kdrD.mod (1295), 144089_km.mod (1296), 144089_naf.mod (1297), 144089_nap.mod (1298), 144089_netstimmm.mod (1299), 144089_sinclamp.mod (1300)

[Shepherd and Brayton, 1979] 144385_ampa.mod (1309), 144385_ca.mod (1310), 144385_cad.mod (1311), 144385_cadiffus.mod (1312), 144385_canmda.mod (1313), 144385_car.mod (1314), 144385_excite.mod (1315), 144385_gabaa.mod (1316), 144385_inhib.mod (1317), 144385_kca.mod (1318), 144385_km.mod (1319), 144385_kv.mod (1320), 144385_na.mod (1321), 144385_nmda.mod (1322)

[Shah et al., 2011] 144392_KahpM95.mod (1323), 144392_cacumm.mod (1324), 144392_cat.mod (1325), 144392_h.mod (1326), 144392_kadist.mod (1327), 144392_kaprox.mod (1328), 144392_kdrca1.mod (1329), 144392_km.mod (1330), 144392_na3n.mod (1331), 144392_naxn.mod (1332)

[Vladimirov et al., 2012] 144401_bias.mod (1333), 144401_cad.mod (1334), 144401_cal.mod (1335), 144401_gap.mod (1336), 144401_ipulse1.mod (1337), 144401_ka.mod (1338), 144401_kahp.mod (1339), 144401_kc.mod (1340), 144401_kdr.mod (1341), 144401_km.mod (1342), 144401_naf.mod (1343)

[Gomez Gonzalez et al., 2011] 144450_KdBG.mod (1344), 144450_NMDAb.mod (1345), 144450_ampa.mod (1346), 144450_cad.mod (1347), 144450_cal.mod (1348), 144450_calH.mod (1349), 144450_can.mod (1350), 144450_car.mod (1351), 144450_cat.mod (1352), 144450_d3.mod (1353), 144450_gabaa.mod (1354), 144450_gabab.mod (1355), 144450_h.mod (1356), 144450_hha2.mod (1357), 144450_hha_old.mod (1358), 144450_ican.mod (1359), 144450_ipulse1.mod (1360), 144450_ipulse2.mod (1361), 144450_kadist.mod (1362), 144450_kaprox.mod (1363), 144450_kca.mod (1364), 144450_kct.mod (1365), 144450_km.mod (1366), 144450_nap.mod (1367), 144450_netstim.mod (1368), 144450_netstimmm.mod (1369), 144450_nmda.mod (1370), 144450_somacar.mod (1371)

[Delgado et al., 2010] 144482_Gfluct.mod (1372), 144482_ampacin.mod (1373), 144482_ar.mod (1374), 144482_ca.mod (1375), 144482_cad.mod (1376), 144482_casoma.mod (1377), 144482_cat.mod (1378), 144482_k2.mod (1379), 144482_k2soma.mod (1380), 144482_ka.mod (1381), 144482_kahp.mod (1382), 144482_kasoma.mod (1383), 144482_kc.mod (1384), 144482_kca.mod (1385), 144482_kdr.mod (1386), 144482_kdrsoma.mod (1387), 144482_km.mod (1388), 144482_kv.mod (1389), 144482_ltpltd.mod (1390), 144482_na.mod (1391), 144482_na1.mod (1392), 144482_naf.mod (1393), 144482_nafsoma.mod (1394), 144482_nap.mod (1395), 144482_nmdacin.mod (1396), 144482_trans.mod (1397)

[Sterratt et al., 2012] 144490_ampa_forti.mod (1398), 144490_cacum.mod (1399), 144490_cad.mod (1400), 144490_cagk.mod (1401), 144490_cagk_v2.mod (1402), 144490_cal.mod (1403), 144490_calH.mod (1404), 144490_calH_v2.mod (1405), 144490_cal_v2.mod (1406), 144490_car.mod (1407), 144490_car_mag.mod (1408), 144490_car_v2.mod (1409), 144490_cat.mod (1410), 144490_cat_v2.mod (1411), 144490_d3.mod (1412), 144490_d3_v2.mod (1413), 144490_gabaa.mod (1414), 144490_gabab.mod (1415), 144490_glutamate.mod (1416), 144490_h.mod (1417), 144490_h_v2.mod (1418), 144490_hha2.mod (1419), 144490_hha2_v2.mod (1420), 144490_hha_old.mod (1421), 144490_hha_old_v2.mod (1422), 144490_kadist.mod (1423), 144490_kadist_v2.mod (1424), 144490_kaprox.mod (1425), 144490_kaprox_v2.mod (1426), 144490_kca.mod (1427), 144490_kca_v2.mod (1428), 144490_km.mod (1429), 144490_km_v2.mod (1430), 144490_nap.mod (1431), 144490_nap_v2.mod (1432), 144490_nmda_andr.mod (1433), 144490_nmda_v2.mod (1434), 144490_somacar.mod (1435), 144490_somacar_v2.mod (1436)

[Aubie et al., 2012] 144511_HH2.mod (1437), 144511_ampa.mod (1438), 144511_gabaa.mod (1439), 144511_nmda.mod (1440)

[DiFrancesco and Noble, 1985] 144520_cadynam.mod (1441), 144520_ibg.mod (1442), 144520_if.mod (1443), 144520_ik1.mod (1444), 144520_ikd.mod (1445), 144520_inaca.mod (1446), 144520_inaf.mod (1447), 144520_inak.mod (1448), 144520_isi.mod (1449), 144520_ito.mod (1450), 144520_kcum.mod (1451), 144520_nacum.mod (1452), 3802_kxph.mod (1953)

[Luthman et al., 2011] 144523_CaConc.mod (1453), 144523_CaHVA.mod (1454), 144523_CaLVA.mod (1455), 144523_CalConc.mod (1456), 144523_DCNsyn.mod (1457), 144523_DCNsynGABA.mod (1458), 144523_DCNsynNMDA.mod (1459), 144523_GammaStim.mod (1460), 144523_NaF.mod (1461), 144523_NaP.mod (1462), 144523_SK.mod (1463), 144523_TNC.mod (1464), 144523_fKdr.mod (1465), 144523_h.mod (1466), 144523_pasDCN.mod (1467), 144523_sKdr.mod (1468)

[Hallermann et al., 2012] 144526_CaH.mod (1469), 144526_CaT.mod (1470), 144526_Cad.mod (1471), 144526_Kca.mod (1472), 144526_Kv.mod (1473), 144526_Kv1_axonal.mod (1474), 144526_Kv7.mod (1475), 144526_charge.mod (1476), 144526_h.mod (1477), 144526_na8st.mod (1478), 144526_nax8st.mod (1479)

[Migliore and Migliore, 2012] 144541_distr.mod (1480), 144541_h.mod (1481), 144541_kadist.mod (1482), 144541_kaprox.mod (1483), 144541_kdrca1.mod (1484), 144541_km.mod (1485), 144541_na3n.mod (1486), 144541_naxn.mod (1487)

[Hopfield and Brody, 2001] 144549_invlfire.mod (1488), 144549_misc.mod (1489), 144549_stats.mod (1490), 144549_vecst.mod (1491)

[Hopfield and Brody, 2000] 144549_invlfire.mod (1488), 144549_misc.mod (1489), 144549_stats.mod (1490), 144549_vecst.mod (1491), 2798_kaprox.mod (1736), 2798_nans.mod (1737)

[Destexhe et al., 1996b] 17663_HH2.mod (1492), 17663_IT2.mod (1493), 17663_VClamp.mod (1494)

[Miyasho et al., 2001] 17664_CaE.mod (1496), 17664_CaP.mod (1497), 17664_CaP2.mod (1498), 17664_CaT.mod (1499), 17664_CalciumP.mod (1500), 17664_K2.mod (1501), 17664_K22.mod (1502), 17664_K23.mod (1503), 17664_KA.mod (1504), 17664_KC.mod (1505), 17664_KC2.mod (1506), 17664_KC3.mod (1507), 17664_KD.mod (1508), 17664_KM.mod (1509), 17664_Kdr.mod (1510), 17664_Kh.mod (1511), 17664_Khh.mod (1512), 17664_Leak.mod (1513), 17664_NaF.mod (1514), 17664_NaP.mod (1515)

[Destexhe et al., 1994c] 18198_HH2.mod (1519), 18198_ampa.mod (1520), 18198_ampa5.mod (1521), 18198_caL3d.mod (1522), 18198_gabaa.mod (1523), 18500_HH.mod (1530), 18500_ampa.mod (1531), 18500_gabaa.mod (1532), 18500_gabab.mod (1533), 18500_nmda.mod (1534)

[Sheasby and Fohlmeister, 1999] 18501_capump.mod (1535), 18501_spike.mod (1536), 18501_spike_icgK2.mod (3483)

[Rusakov et al., 1997] 18502_cabpump.mod (1537), 18502_cachan.mod (1538), 18502_cadifusl.mod (1539), 18502_cagk.mod (1540), 18502_nmdasyn.mod (1541)

[Rusakov et al., 1996] 18502_cabpump.mod (1537), 18502_cachan.mod (1538), 18502_cadifusl.mod (1539), 18502_cagk.mod (1540), 18502_nmdasyn.mod (1541)

[Tsutsui and Oka, 2002] 18742_Na_cglc.mod (1542)

[Geiger et al., 1997] 19022_basket_syn.mod (1543)

[Wang et al., 2002] 19176_HH2.mod (1544), 19176_cAMPclamp.mod (1545), 19176_hcn2.mod (1546), 19176_kleak.mod (1547)

[Korogod and Kulagina, 1998] 19214_PasD.mod (1548), 19214_PasS.mod (1549), 19214_PasSA.mod (1550), 19214_hh1.mod (1551)

[Carlin et al., 2000] 19491_L_Ca.mod (1554), 19491_L_HVA_Ca.mod (1555), 19491_N_Ca.mod (1556), 19491_R_VClamp.mod (1557)

[Gabbiani et al., 1994] 19591_CaHVA_chan.mod (1558), 19591_Icinst.mod (1559), 19591_Ka_chan.mod (1560), 19591_Kdr_chan.mod (1561), 19591_Na_chan.mod (1562), 19591_cagrcdifus.mod (1563), 19591_caleak.mod (1564), 19591_h_chan.mod (1565), 19591_nacaexch.mod (1566), 19591_naleak.mod (1567)

[Migliore, 2003] 19696_h.mod (1568), 19696_kadist.mod (1569), 19696_kaprox.mod (1570), 19696_kdrca1.mod (1571), 19696_na3.mod (1572), 19696_nax.mod (1573)

[Graham, 2001] 19746_ANsyn.mod (1574), 19746_BKDNaDR.mod (1575), 19746_CA1CaG.mod (1576), 19746_CA1NaP.mod (1577), 19746_DEsyn.mod (1578), 19746_L98NCh.mod (1579), 19746_M99Ka.mod (1580)

[McRory et al., 2001] 19920_ca_a1g.mod (1584), 19920_ca_a1h.mod (1585), 19920_ca_a1i.mod (1586)

[Lazarewicz et al., 2002] 20007_CAlM95.mod (1587), 20007_CAnM95.mod (1588), 20007_CAtM95.mod (1589), 20007_KaDM99SL.mod (1590), 20007_KaPM99SL.mod (1591), 20007_KahpM95.mod (1592), 20007_KctBG99.mod (1593), 20007_KdBG.mod (1594), 20007_KdrM99SL.mod (1595), 20007_KhdM01.mod (1596), 20007_KmM95.mod (1597), 20007_NaM99SL.mod (1598), 20007_caM95.mod (1599)

[Lien et al., 2002] 20015_ka.mod (1604), 20015_kdrf.mod (1605), 20015_kdrs.mod (1606)

[Poirazi et al., 2003a]

[Poirazi et al., 2003b]

[Polsky et al., 2004] 20212_ITGHK.mod (1607), 20212_My_cal.mod (1608), 20212_My_can.mod (1609), 20212_My_cat.mod (1610), 20212_Myca.mod (1611), 20212_Mykca.mod (1612), 20212_abbott.mod (1613), 20212_abbott_nmda.mod (1614), 20212_borgkm.mod (1615), 20212_ca.mod (1616), 20212_cachan.mod (1617), 20212_cacum.mod (1618), 20212_cad.mod (1619), 20212_cad3.mod (1620), 20212_cad_trunk.mod (1621), 20212_cadecay.mod (1622), 20212_cadifusl.mod (1623), 20212_cagk.mod (1624), 20212_cagk_v2.mod (1625), 20212_cal.mod (1626), 20212_calH.mod (1627), 20212_calH_v2.mod (1628), 20212_cal_v2.mod (1629), 20212_can2.mod (1630), 20212_canKev.mod (1631), 20212_capump.mod (1632), 20212_car.mod (1633), 20212_cat.mod (1634), 20212_cat_v2.mod (1635), 20212_d3.mod (1636), 20212_gabaa.mod (1637), 20212_gabab.mod (1638), 20212_glutamate.mod (1639), 20212_h.mod (1640), 20212_h_v2.mod (1641), 20212_hh3.mod (1642), 20212_hh3_flei.mod (1643), 20212_hha2.mod (1644), 20212_hha_old.mod (1645), 20212_hha.mod (1646), 20212_ht.mod (1647), 20212_ican.mod (1648), 20212_iq.mod (1649), 20212_it.mod (1650), 20212_it1.mod (1651), 20212_kad.mod (1652), 20212_kadist.mod (1653), 20212_kap.mod (1654), 20212_kaprox.mod (1655), 20212_kc.mod (1656), 20212_kca.mod (1657), 20212_kca_v2.mod (1658), 20212_kdr.mod (1659), 20212_kdr_inac.mod (1660), 20212_kdrca1.mod (1661), 20212_km.mod (1662), 20212_kv.mod (1663), 20212_my_kca.mod (1664), 20212_na3.mod (1665), 20212_na.mod (1666), 20212_namir.mod (1667), 20212_nap.mod (1668), 20212_nax.mod (1669), 20212_nmda.mod (1670), 20212_ourca_old.mod (1671), 20212_somacar.mod (1672), 20212_vkca.mod (1673)

[Traub et al., 2003] 20756_ar.mod (1674), 20756_cad.mod (1675), 20756_cal.mod (1676), 20756_cat.mod (1677), 20756_k2.mod (1678), 20756_ka.mod (1679), 20756_kahp.mod (1680), 20756_kc.mod (1681), 20756_kdr.mod (1682), 20756_km.mod (1683), 20756_naf.mod (1684), 20756_nap.mod (1685)

[Bartos et al., 2002] 21329_gap.mod (1686), 21329_nethhwbm.mod (1687)

[Schaefer et al., 2007] 22203_gDKma.mod (1688), 22203_kadist.mod (1689), 22203_kaprox.mod (1690), 22203_kfast.mod (1691), 22203_km.mod (1692), 22203_kslow.mod (1693), 22203_kv.mod (1694), 22203_kvfast.mod (1695)

[Schaefer et al., 2003a]

[Davison et al., 2000] 2487_cadecay.mod (1696), 2487_kA.mod (1697), 2487_kca3.mod (1698), 2487_kfasttab.mod (1699), 2487_kslowtab.mod (1700), 2487_lcafixed.mod (1701), 2487_nafast.mod (1702)

[Mainen and Sejnowski, 1996] 2488_ca.mod (1703), 2488_cad.mod (1704), 2488_kca.mod (1705), 2488_km.mod (1706), 2488_kv.mod (1707), 2488_na.mod (1708)

[Wang and Buzsaki, 1996] 26997_fvpre.mod (1709), 26997_kdr.mod (1710), 26997_naf.mod (1711), 26997_vecst.mod (1712)

[Davison et al., 2003] 2730_cadecay.mod (1713), 2730_flushf.mod (1714), 2730_kA.mod (1715), 2730_kM.mod (1716), 2730_kca.mod (1717), 2730_kfasttab.mod (1718), 2730_kslowtab.mod (1719), 2730_lcafixed.mod (1720), 2730_nafast.mod (1721), 2730_nagran.mod (1722), 2730_nmdanet.mod (1723)

[Bhalla and Bower, 1993] 2733_cadecay.mod (1724), 2733_kA.mod (1725), 2733_kca3.mod (1726), 2733_kfasttab.mod (1727), 2733_kslowtab.mod (1728), 2733_lcafixed.mod (1729), 2733_nafast.mod (1730)

[Migliore et al., 1999] 2796_kadist.mod (1731), 2796_kaprox.mod (1732), 2796_kdrca1.mod (1733), 2796_na3.mod (1734), 2796_nax.mod (1735)

[Destexhe et al., 1998b] 279_ITGHK.mod (1738), 279_VClamp.mod (1739), 279_cadecay.mod (1740), 279_hh2.mod (1741)

[Saraga et al., 2003] 28316_IA.mod (1742), 28316_IAprox.mod (1743), 28316_Ih.mod (1744), 28316_Kaxon.mod (1745), 28316_Kdend.mod (1746), 28316_Ksoma.mod (1747), 28316_Naaxon.mod (1748), 28316_Nadend.mod (1749), 28316_Nasoma.mod (1750), 28316_ampa.mod (1751), 28316_pregen.mod (1752)

[Migliore, 1996] 2937_borgkm.mod (1753), 2937_iq.mod (1754), 2937_kdr.mod (1755), 2937_namir.mod (1756)

[Migliore et al., 2001] 3167_kaprox.mod (1757), 3167_kdrca1.mod (1758), 3167_na3.mod (1759), 3167_pnoise.mod (1760)

[Migliore et al., 1995] 3263_borgka.mod (1761), 3263_borgkm.mod (1762), 3263_cadiv.mod (1763), 3263_cagk.mod (1764), 3263_cal2.mod (1765), 3263_can2.mod (1766), 3263_cat.mod (1767), 3263_kahp.mod (1768), 3263_kdr.mod (1769), 3263_nahh.mod (1770)

[Varela et al., 1997] 3264_fdsexp2s.mod (1771), 3264_fdsexpsn.mod (1772), 3264_fdsintf.mod (1773)

[Korngreen and Sakmann, 2000] 3289_kfast.mod (1774), 3289_kslow.mod (1775)

[Migliore et al., 2004] 32992_distr.mod (1776), 32992_h.mod (1777), 32992_kadist.mod (1778), 32992_kaprox.mod (1779), 32992_kdrca1.mod (1780), 32992_na3n.mod (1781), 32992_naxn.mod (1782), 32992_netstimm.mod (1783)

[Bal and Oertel, 2000] 3332_hcno.mod (1784)

[Shen et al., 1999] 3342_kd.mod (1785), 3342_kd_v2.mod (1786), 3342_na.mod (1787), 3342_na_v2.mod (1788)

[Destexhe et al., 1996a] 3343_HH2.mod (1789), 3343_IT.mod (1790), 3343_IT2.mod (1791), 3343_Ih.mod (1792), 3343_ampa.mod (1793), 3343_cadecay.mod (1794), 3343_gabaa.mod (1795), 3343_gabab.mod (1796), 3343_kleak.mod (1797)

[Beck et al., 1992] 3344_kadg.mod (1798)

[Lytton and Hines, 2005] 33975_GABAA.mod (1799), 33975_cur.mod (1800), 33975_fvpre.mod (1801), 33975_kdr.mod (1802), 33975_naf.mod (1803)

[Wang et al., 1998] 3454_ht.mod (1816), 3454_lt.mod (1817), 3454_nach.mod (1818), 3454_trigstim.mod (1819)

[Benison et al., 2001] 3457_calrgc.mod (1820), 3457_canrgc.mod (1821), 3483_nargc.mod (1822), 3488_kargc.mod (1823)

[Huang and Robinson, 1998] 3457_calrgc.mod (1820), 3457_canrgc.mod (1821)

[Skaliora et al., 1995] 3491_kdrgc.mod (1824)

[Safronov and Vogel, 1995] 3493_ka_moto.mod (1825), 3493_kdr_moto.mod (1826), 3493_na_moto.mod (1827)

[FRANKENHAEUSER and HUXLEY, 1964] 3507_fh.mod (1828)

[Moczydlowski and Latorre, 1983] 3509_cagk.mod (1829)

[Nadim et al., 1998] 3511_gap.mod (1830), 3511_gapvdep.mod (1831), 3511_int1.mod (1832), 3511_int1_lg.mod (1833), 3511_int1s.mod (1834), 3511_lg.mod (1835), 3511_lg_int1.mod (1836), 3511_lg_mcn1.mod (1837), 3511_mcn1.mod (1838), 3511_mcn1_int1.mod (1839), 3511_mcn1_lg.mod (1840), 3511_pass.mod (1841)

[Williams and Stuart, 2000] 3533_cat_thal.mod (1842)

[Pinsky and Rinzel, 1994] 35358_cal.mod (1843), 35358_kcRT03.mod (1844), 35358_kdr.mod (1845), 35358_matrix.mod (1846), 35358_nafPR.mod (1847), 35358_passiv.mod (1848), 35358_rcadecay.mod (1849), 35358_rkq.mod (1850), 35358_vecst.mod (1851)

[Wang et al., 1996] 3648_kamt.mod (1852), 3648_kdrmt.mod (1853)

[Saftenku, 2005] 3658_ampad2.mod (1854), 3658_ampad4.mod (1855), 3658_glubbfbm.mod (1856), 3658_glubes2.mod (1857), 3658_glubes23.mod (1858), 3658_glubes3.mod (1859), 3658_glubes4.mod (1860), 3658_glubes5.mod (1861), 3658_glubes6.mod (1862), 3658_gludif2.mod (1863), 3658_gludif23.mod (1864), 3658_gludif3.mod (1865), 3658_glures23.mod (1866), 3658_spgen2.mod (1867)

[Zhou and Hablitz, 1996] 3660_kaneosup.mod (1868), 3660_kdrneosup.mod (1869)

[Cadetti and Belluzzi, 2001] 3665_hpg.mod (1870)

[Destexhe et al., 1994a] 3670_HH2.mod (1871), 3670_IAHP.mod (1872), 3670_ICAN.mod (1873), 3670_IT2.mod (1874), 3670_capump.mod (1875), 3670_gabaa.mod (1876), 3670_gabab1.mod (1877)

[Fohlmeister and Miller, 1997] 3673_capump.mod (1878), 3673_spike.mod (1879), 3673_spike_icgK2.mod (3484)

[Stuart and Spruston, 1998] 3677_iqq.mod (1880), 3677_synampa.mod (1881)

[Archie and Mel, 2000] 3682_hh3.mod (1882), 3682_rglu_score.mod (1883)

[McCormick and Pape, 1990] 3684_htc.mod (1884)

[Bazhenov et al., 1998] 37819_AMPA.mod (1885), 37819_GABAA.mod (1886), 37819_GABAB2.mod (1887), 37819_HH2.mod (1888), 37819_IT.mod (1889), 37819_IT2.mod (1890), 37819_Iahp.mod (1891), 37819_Ican.mod (1892), 37819_Ih.mod (1893), 37819_NMDA.mod (1894), 37819_burst.mod (1895), 37819_ca.mod (1896), 37819_cad.mod (1897), 37819_cadecay.mod (1898), 37819_gabab.mod (1899), 37819_gababS.mod (1900), 37819_intf.mod (1901), 37819_kca.mod (1902), 37819_kdr.mod (1903), 37819_kl.mod (1904), 37819_kleak.mod (1905), 37819_km.mod (1906), 37819_kmbg.mod (1907), 37819_kv.mod (1908), 37819_matrix.mod (1909), 37819_na.mod (1910), 37819_naf.mod (1911), 37819_nap.mod (1912), 37819_nstim.mod (1913), 37819_ntia.mod (1914), 37819_tcifb.mod (1915), 37819_tia.mod (1916), 37819_vecst.mod (1917)

[Kanold and Manis, 2001] 37856_Ksyn.mod (1918), 37856_Stim.mod (1919), 37856_pyr.mod (1920), 37856_pyr_icgK2.mod (3485), 37856_pyr_icgK3.mod (3486)

[Kanold and Manis, 1999] 37856_Ksyn.mod (1918), 37856_Stim.mod (1919), 37856_pyr.mod (1920), 37856_pyr_icgK2.mod (3485), 37856_pyr_icgK3.mod (3486)

[Rothman and Manis, 2003c]

[Rothman and Manis, 2003b]

[Rothman and Manis, 2003a] 37857_hcno.mod (1921), 37857_ih.mod (1922), 37857_ka.mod (1923), 37857_kht.mod (1924), 37857_klt.mod (1925), 37857_leak.mod (1926), 37857_na.mod (1927)

[Crane et al., 2001] 3785_couple5.mod (1928)

[Chen et al., 2002] 3793_glusyn.mod (1929), 3793_inhisyn.mod (1930), 3793_kd.mod (1931), 3793_na.mod (1932)

[Hines and Shrager, 1991] 3798_fh.mod (1933)

[Courtemanche et al., 1998] 3800_cadynam.mod (1934), 3800_cajsr.mod (1935), 3800_cajsracc.mod (1936), 3800_cansracc.mod (1937), 3800_capump.mod (1938), 3800_ical.mod (1939), 3800_ik1.mod (1940), 3800_ikr.mod (1941), 3800_iks.mod (1942), 3800_ikur.mod (1943), 3800_inaf.mod (1944), 3800_ionback.mod (1945), 3800_ito.mod (1946), 3800_kcum.mod (1947), 3800_nacaex.mod (1948), 3800_nacum.mod (1949), 3800_nipace.mod (1950), 3800_nkpump.mod (1951)

[Bartos et al., 2001] 3801_basket_syn.mod (1952)

[Baccus, 1998] 3807_caacum.mod (1955), 3807_cachlech.mod (1956), 3807_capump.mod (1957), 3807_gkca.mod (1958), 3807_naacum.mod (1959), 3807_napump.mod (1960), 3807_pcell.mod (1961)

[McIntyre et al., 2002] 3810_AXNODE.mod (1985), 3810_AXNODE_icgNA2.mod (3492)

[Anderson et al., 1999] 3812_nmda.mod (1986), 3812_two_exp.mod (1987)

[Tsodyks et al., 1998] 3815_tmgsyn.mod (1988)

[Destexhe et al., 1998a] 3817_IM.mod (1989), 3817_IT.mod (1990), 3817_cadecay.mod (1991), 3817_hh2.mod (1992)

[Izhikevich, 2003] 39948_izh.mod (1993)

[Gruber et al., 2003] 39949_caL.mod (1994), 39949_damsg.mod (1995), 39949_gspike.mod (1996), 39949_kir2.mod (1997), 39949_ksi.mod (1998), 39949_leak.mod (1999)

[Migliore et al., 2005b] 43039_kamt.mod (2000), 43039_kdrmt.mod (2001), 43039_naxn.mod (2002)

[Gasparini et al., 2004] 44050_exp2i.mod (2003), 44050_h.mod (2004), 44050_kadist.mod (2005), 44050_kaprox.mod (2006), 44050_kdrca1.mod (2007), 44050_na3s.mod (2008), 44050_naxn.mod (2009), 44050_netstims.mod (2010), 44050_nmdanet.mod (2011)

[D'Angelo et al., 2001] 46839_Grc_kca.mod (2012), 46839_Grc_lkg2.mod (2013), 46839_Grc_sine.mod (2014), 46839_Grg_kv.mod (2015), 46839_grc_ca.mod (2016), 46839_grc_calc.mod (2017), 46839_grc_ka.mod (2018), 46839_grc_kir.mod (2019), 46839_grc_lkg1.mod (2020), 46839_grc_pna.mod (2021), 46839_grg_km.mod (2022), 46839_grg_na.mod (2023), 46839_grg_nar.mod (2024)

[Khaliq et al., 2003] 48332_bkpkj.mod (2025), 48332_cadiff.mod (2026), 48332_cap.mod (2027), 48332_ihpkj.mod (2028), 48332_kpkj.mod (2029), 48332_kpkj2.mod (2030), 48332_kpkjslow.mod (2031), 48332_pkjlk.mod (2032), 48332_rsg.mod (2033)

[Raman and Bean, 2001] 48332_bkpkj.mod (2025), 48332_cadiff.mod (2026), 48332_cap.mod (2027), 48332_ihpkj.mod (2028), 48332_kpkj.mod (2029), 48332_kpkj2.mod (2030), 48332_kpkjslow.mod (2031), 48332_pkjlk.mod (2032), 48332_rsg.mod (2033)

[Kampa et al., 2004] 50207_NMDA_Mg.mod (2034), 50207_release_BMK.mod (2035)

[Saghatelyan et al., 2005] 50210_kamt.mod (2036), 50210_kdrmt.mod (2037), 50210_naxn.mod (2038), 50210_nmdanet.mod (2039)

[Sikora et al., 2005] 50997_caconc.mod (2040), 50997_cagf.mod (2041), 50997_capump.mod (2042), 50997_casimple.mod (2043), 50997_modulate.mo (2044), 50997_rib_ufp.mod (2045), 50997_ribbon_tiger.mod (2046), 50997_spike.mod (2047), 50997_tsbp.mod (2048), 50997_vclmp.mod (2049), 50997_spike_icgK2.mod (3487)

[Amir and Devor, 2003a] 51022_fh.mod (2050)

[Amir and Devor, 2003b] 51022_fh.mod (2050)

[Bush et al., 1999] 52034_AMPA.mod (2078), 52034_GABAa.mod (2079), 52034_GABAb.mod (2080), 52034_NMDA.mod (2081), 52034_cadecay.mo (2082), 52034_cah.mod (2083), 52034_dpresyn.mod (2084), 52034_fpoisson_generator.mod (2085), 52034_glu.mod (2086), 52034_holt_alphasyn.mod (2087), 52034_holt_rnd.mod (2088), 52034_kca.mod (2089), 52034_kdr.mod (2090), 52034_kdrp.mod (2091), 52034_na.mod (2092), 52034_nap.mod (2093), 52034_noise.mod (2094), 52034_precall.mod (2095), 52034_pregen.mod (2096), 52034_seed.mod (2097)

[Popovic et al., 2005] 53435_EPSPClamp.mod (2098), 53435_EPSPClamp_v2.mod (2099)

[Hines and Carnevale, 2001] 53451_ca.mod (2100), 53451_cad.mod (2101), 53451_kca.mod (2102), 53451_km.mod (2103), 53451_kv.mod (2104), 53451_na.mod (2105), 53451_peak.mod (2106)

[Prescott and De Koninck, 2005] 53569_CaP.mod (2107), 53569_CaT.mod (2108), 53569_HH2new.mod (2109), 53569_Kslow.mod (2110), 53569_NaP.mod (2111), 53569_cadyn.mod (2112)

[Zhou et al., 2005] 53869_Alpha.mod (2113), 53869_gaussstim.mod (2114), 53869_ih_VCN2003.mod (2115), 53869_kHT_VCN2003.mod (2116), 53869_kLT_VCN2003.mod (2117), 53869_na_VCN2003.mod (2118)

[Quadroni and Knopfel, 1994] 53876_a.mod (2119), 53876_ahp.mod (2120), 53876_akfast.mod (2121), 53876_bkfast.mod (2122), 53876_ca_dist.mo (2123), 53876_ca_prox.mod (2124), 53876_ca_soma.mod (2125), 53876_ca_soma_const_delta_thick_shells.mod (2126), 53876_ca_soma_cylind_shells.mod (2127), 53876_caleak.mod (2128), 53876_clleak.mod (2129), 53876_h.mod (2130), 53876_hva.mod (2131), 53876_kleak.mod (2132), 53876_kslow.mod (2133), 53876_lva.mod (2134), 53876_na.mod (2135), 53876_naleak.mod (2136), 53876_nap.mod (2137), 53876_nmda.mod (2138)

[Wang et al., 1991] 53893_lva.mod (2139)

[Vitko et al., 2005] 53965_HH2.mod (2140), 53965_IT2.mod (2141), 53965_IT2_v2.mod (2142), 53965_IT2_v3.mod (2143), 53965_VClamp.mod (2144), 53965_capump.mod (2145)

[Hossain et al., 2005] 54903_km.mod (2146), 54903_kv.mod (2147), 54903_na.mod (2148), 54903_nach.mod (2149), 54903_naf.mod (2150), 54903_naxn.mod (2151)

[Migliore et al., 2005a] 55035_distr.mod (2152), 55035_h.mod (2153), 55035_kadist.mod (2154), 55035_kaprox.mod (2155), 55035_kdrca1.mod (2156), 55035_na3n.mod (2157), 55035_naxn.mod (2158), 55035_netstims.mod (2159)

[Safronov et al., 2000] 62266_B_A.mod (2160), 62266_B_DR.mod (2161), 62266_B_NA.mod (2162)

[Melnick et al., 2004a] 62284_B_A.mod (2163), 62284_B_DR.mod (2164), 62284_B_NA.mod (2165), 62284_KDR.mod (2166), 62284_KDRI.mod (2167), 62284_SS.mod (2168)

[Melnick et al., 2004b] 62285_B_A.mod (2169), 62285_B_DR.mod (2170), 62285_B_NA.mod (2171), 62285_KDR.mod (2172), 62285_KDRI.mod (2173), 62285_SS.mod (2174)

[Baranauskas and Martina, 2006] 62673_kdersej.mod (2175), 62673_mcna1e.mod (2176), 62673_nachansej.mod (2177)

[Kole et al., 2006] 64195_ca.mod (2178), 64195_caT.mod (2179), 64195_cad.mod (2180), 64195_ih_stochastic.mod (2181), 64195_ih.mod (2182), 64195_ka.mod (2183), 64195_kca.mod (2184), 64195_km.mod (2185), 64195_kv.mod (2186), 64195_na.mod (2187), 64195_na_stochastic.mod (2188), 64195_syn.mod (2189)

[Shimazaki et al., 2006] 64212_kavn.mod (2190), 64212_kdr.mod (2191), 64212_navn.mod (2192)

[Kourennyi et al., 2004] 64216_CPR.mod (2193), 64216_Kv.mod (2194), 64216_h.mod (2195), 95870_CPR.mod (2529), 95870_Ca.mod (2530), 95870_Cad.mod (2531), 95870_IinjLT.mod (2532), 95870_Kv.mod (2533), 95870_Kx.mod (2534), 95870_Leak.mod (2535), 95870_hyper.mod (2536)

[Liu and Kourennyi, 2004] 64228_Ca.mod (2196), 64228_Cad.mod (2197), 64228_Clca.mod (2198), 64228_IinjLT.mod (2199), 64228_IinjLTDim.mo (2200), 64228_IinjSin.mod (2201), 64228_Kca.mod (2202), 64228_Kv.mod (2203), 64228_Kx.mod (2204), 64228_Leak.mod (2205), 64228_h.mod (2206), 95870_CPR.mod (2529), 95870_Ca.mod (2530), 95870_Cad.mod (2531), 95870_IinjLT.mod (2532), 95870_Kv.mod (2533), 95870_Kx.mod (2534), 95870_Leak.mod (2535), 95870_hyper.mod (2536)

[Migliore et al., 2006] 64229_AMPA.mod (2207), 64229_AMPA_v2.mod (2208), 64229_CaBK.mod (2209), 64229_GABAa.mod (2210), 64229_GABAa_v2.mod (2211), 64229_GABAb.mod (2212), 64229_GABAb_v2.mod (2213), 64229_Gfluct2.mod (2214), 64229_LcaMig.mod (2215), 64229_NMDA.mod (2216), 64229_NMDA_v2.mod (2217), 64229_arhRT03.mod (2218), 64229_arhRT03_v2.mod (2219), 64229_bgka.mod (2220), 64229_cadRT03.mod (2221), 64229_cadRT03_v2.mod (2222), 64229_cadecay.mod (2223), 64229_cadecay_v2.mod (2224), 64229_cadecay_v3.mod (2225), 64229_cah.mod (2226), 64229_cah_v2.mod (2227), 64229_calRT03.mod (2228), 64229_calRT03_v2.mod (2229), 64229_catRT03.mod (2230), 64229_catRT03_v2.mod (2231), 64229_ccanl.mod (2232), 64229_flushf.mod (2233), 64229_gskch.mod (2234), 64229_hyperde3.mod (2235), 64229_ichan2.mod (2236), 64229_intf.mod (2237), 64229_intf_v2.mod (2238), 64229_invlfire.mod (2239), 64229_k2RT03.mod (2240), 64229_k2RT03_v2.mod (2241), 64229_kA.mod (2242), 64229_kM.mod (2243), 64229_kaRT03.mod (2244), 64229_kaRT03_v2.mod (2245), 64229_kahpRT03.mod (2246), 64229_kahpRT03_v2.mod (2247), 64229_kcRT03.mod (2248), 64229_kcRT03_v2.mod (2249), 64229_kca.mod (2250), 64229_kca_v2.mod (2251), 64229_kca_v3.mod (2252), 64229_kdr.mod (2253), 64229_kdrRT03.mod (2254), 64229_kdrRT03_v2.mod (2255), 64229_kdr_v2.mod (2256), 64229_kdrp.mod (2257), 64229_kdrp_v2.mod (2258), 64229_kfasttab.mod (2259), 64229_kmRT03.mod (2260), 64229_kmRT03_v2.mod (2261), 64229_kslowtab.mod (2262), 64229_lcafixed.mod (2263), 64229_misc.mod (2264), 64229_misc_v2.mod (2265), 64229_myfit.mod (2266), 64229_na.mod (2267), 64229_na_v2.mod (2268), 64229_nafRT03.mod (2269), 64229_nafRT03_v2.mod (2270), 64229_nafast.mod (2271), 64229_nagran.mod (2272), 64229_nap.mod (2273), 64229_napRT03.mod (2274), 64229_napRT03_v2.mod (2275), 64229_nap_v2.mod (2276), 64229_nca.mod (2277), 64229_nmdanet.mod (2278), 64229_nstim.mod (2279), 64229_nstim_v2.mod (2280), 64229_stats.mod (2281), 64229_tca.mod (2282), 64229_vecst.mod (2283), 64229_vecst_v2.mod (2284), 64229_hyperde3_icgIH2.mod (3474), 64229_ichan2_icgK2.mod (3489)

[Rudolph and Destexhe, 2005] 64259_Gfluct.mod (2285)

[Davison and Fregnac, 2006] 64261_ctrlnsvr2.mod (2286), 64261_flushf.mod (2287), 64261_netstimvr2.mod (2288), 64261_stdwa_softlimits.mod (2289), 64261_stdwa_songabbott.mod (2290), 64261_stdwa_symm.mod (2291)

[Golomb et al., 2006] 64296_INaP.mod (2292), 64296_Ih.mod (2293), 64296_cadecay.mod (2294), 64296_kA.mod (2295), 64296_kO.mod (2296), 64296_kca3.mod (2297), 64296_kfasttab.mod (2298), 64296_kslowtab.mod (2299), 64296_lcafixed.mod (2300), 64296_nafast.mod (2301)

[Canavier, 1999] 6763_delay.mod (2302), 6763_inmda.mod (2303), 6763_leak.mod (2304), 6763_mystim.mod (2305), 6763_nadifl.mod (2306), 6763_pump.mod (2307)

[Li and Ascoli, 2006]

[Li and Ascoli, 2008] 71312_distr.mod (2308), 71312_h.mod (2309), 71312_kadist.mod (2310), 71312_kaprox.mod (2311), 71312_kdrca1.mod (2312), 71312_na3n.mod (2313), 71312_naxn.mod (2314)

[Watanabe et al., 2002] 7386_kadist.mod (2315), 7386_kaprox.mod (2316), 7386_kdrca1.mod (2317), 7386_na3.mod (2318), 7386_nax.mod (2319)

[Lytton, 1998] 7399_AMPA.mod (2320), 7399_GABAA.mod (2321), 7399_kdr.mod (2322), 7399_matrix.mod (2323), 7399_naf.mod (2324), 7399_passiv.mod (2325), 7399_precall.mod (2326), 7399_pregen.mod (2327), 7399_pregencv.mod (2328), 7399_pulsecv.mod (2329), 7399_sinstim.mod (2330), 7399_vecst.mod (2331)

[Lytton and Lipton, 1999] 7400_AMPA.mod (2332), 7400_GABAA.mod (2333), 7400_kdr.mod (2334), 7400_matrix.mod (2335), 7400_naf.mod (2336), 7400_passiv.mod (2337), 7400_pulse.mod (2338), 7400_sinstim.mod (2339), 7400_vecst.mod (2340)

[Gillies and Willshaw, 2006] 74298_CaT.mod (2341), 74298_Cacum.mod (2342), 74298_HVA.mod (2343), 74298_Ih.mod (2344), 74298_KDR.mod (2345), 74298_Kv31.mod (2346), 74298_Na.mod (2347), 74298_NaL.mod (2348), 74298_STh.mod (2349), 74298_sKCa.mod (2350)

[Migliore and Shepherd, 2002] 7659_distr.mod (2351), 7659_h.mod (2352)

[Wilson and Cowan, 1972] 76879_wc.mod (2353)

[Chen et al., 2001b] 7988_IVnmda5.mod (2354), 7988_glurel.mod (2355), 7988_glurelex.mod (2356), 7988_nmda5.mod (2357), 7988_release.mod (2358), 7988_relramp.mod (2359)

[Akemann and Knopfel, 2006] 80769_CaBK.mod (2360), 80769_CaP.mod (2361), 80769_Caint.mod (2362), 80769_Ih.mod (2363), 80769_Kbin.mod (2364), 80769_Kv1.mod (2365), 80769_Kv4.mod (2366), 80769_Na.mod (2367), 80769_Narsg.mod (2368), 80769_leak.mod (2369)

[Destexhe et al., 2001] 8115_Gfluct.mod (2370), 8115_IKd_traub.mod (2371), 8115_IM.mod (2372), 8115_INa_traub_shifted.mod (2373)

[Mainen et al., 1995] 8210_kd3h5.mod (2374), 8210_na3h5.mod (2375)

[van Welie et al., 2006] 82364_h.mod (2376)

[Durstewitz and Gabriel, 2007] 82784_HVA.mod (2377), 82784_Kc.mod (2378), 82784_Kdr.mod (2379), 82784_KdrIN.mod (2380), 82784_Ks.mod (2381), 82784_Naf.mod (2382), 82784_NafIN.mod (2383), 82784_Nap.mod (2384), 82784_NapDA.mod (2385), 82784_ampa.mod (2386), 82784_cadyn.mod (2387), 82784_gaba.mod (2388), 82784_kdyn.mod (2389), 82784_nmda.mod (2390), 82784_nmdacons.mod (2391)

[Durstewitz et al., 2000] 82849_cadyn.mod (2392), 82849_hva.mod (2393), 82849_ic.mod (2394), 82849_iks.mod (2395), 82849_kdr.mod (2396), 82849_kdyn.mod (2397), 82849_naf.mod (2398), 82849_nap.mod (2399)

[Chen et al., 2001a] 8284_hyperde1.mod (2400), 8284_hyperde2.mod (2401), 8284_hyperde3.mod (2402), 8284_hyperso.mod (2403), 8284_ichan.mod (2404), 8284_ppsyn.mod (2405), 8284_hyperde1_icgIH2.mod (3467), 8284_hyperde2_icgIH2.mod (3468), 8284_hyperde3_icgIH2.mod (3469), 8284_hyperso_icgIH2.mod (3470)

[Brette et al., 2007] 83319_if3.mod (2406), 83319_intfirecur.mod (2407), 83319_spikeout.mod (2408)

[Vogels and Abbott, 2005] 83319_if3.mod (2406), 83319_intfirecur.mod (2407), 83319_spikeout.mod (2408)

[Korngreen et al., 2005] 83523_kfast.mod (2409), 83523_kv.mod (2410)

[Komendantov et al., 2004] 83547_ampa.mod (2411), 83547_cadif.mod (2412), 83547_calcha.mod (2413), 83547_capump.mod (2414), 83547_hh3.mod (2415), 83547_kca.mod (2416), 83547_leak.mod (2417), 83547_nadif.mod (2418), 83547_nmda.mod (2419), 83547_pump.mod (2420), 83547_stim.mod (2421), 83547_hh3_icgK2.mod (3490)

[Arsiero et al., 2007] 83590_Ifluct1.mod (2422), 83590_hh_in.mod (2423)

[Holt and Koch, 1999]

[Gold et al., 2006]

[Gold et al., 2007] 84589_cadif.mod (2424), 84589_cal.mod (2425), 84589_can.mod (2426), 84589_car.mod (2427), 84589_cat.mod (2428), 84589_hdend.mod (2429), 84589_hsoma.mod (2430), 84589_kadist.mod (2431), 84589_kahp.mod (2432), 84589_kaprox.mod (2433), 84589_kc.mod (2434), 84589_kd.mod (2435), 84589_kk.mod (2436), 84589_km.mod (2437), 84589_naf.mod (2438), 84589_nax.mod (2439)

[Canavier and Landry, 2006] 84612_ampa.mod (2440), 84612_ampasyn.mod (2441), 84612_cabalan.mod (2442), 84612_cachan.mod (2443), 84612_capump.mod (2444), 84612_hh3.mod (2445), 84612_kca.mod (2446), 84612_leak.mod (2447), 84612_nabalan.mod (2448), 84612_nmdaL.mod (2449), 84612_nmdasyn.mod (2450), 84612_pump.mod (2451), 84612_stim.mod (2452), 84612_hh3_icgK2.mod (3491)

[Oltedal et al., 2007] 84655_gsynth.mod (2453)

[Mennerick et al., 1997] 84655_gsynth.mod (2453)

[Baker, 2005] 85112_kf.mod (2454), 85112_ks.mod (2455), 85112_nap.mod (2456), 85112_nattxs.mod (2457), 85112_nav1p8.mod (2458), 85112_nav1p9.mod (2459)

[Herzog et al., 2001] 86537_kdr.mod (2460), 86537_nattxs.mod (2461), 86537_nav1p9.mod (2462)

[Morse et al., 2010] 87284_cacumm.mod (2463), 87284_cagk.mod (2464), 87284_cal2.mod (2465), 87284_can2.mod (2466), 87284_cat.mod (2467), 87284_distr.mod (2468), 87284_h.mod (2469), 87284_ipulse2.mod (2470), 87284_kadist.mod (2471), 87284_kaprox.mod (2472), 87284_kdrca1.mod (2473), 87284_na3n.mod (2474), 87284_naxn.mod (2475), 87284_zcaquant.mod (2476)

[Migliore et al., 2008b] 87535_h.mod (2488), 87535_kadist.mod (2489), 87535_kaprox.mod (2490), 87535_kdrca1.mod (2491), 87535_na3n.mod (2492), 87535_naxn.mod (2493), 87535_netstims.mod (2494)

[Minneci et al., 2007] 87546_kamt.mod (2495), 87546_ks.mod (2496), 87546_naxn.mod (2497)

[Barela et al., 2006] 87585_ichanR859C1.mod (2498), 87585_ichanWT2005.mod (2499)

[Spampanato et al., 2004] 87585_ichanR859C1.mod (2498), 87585_ichanWT2005.mod (2499)

[Liu et al., 1998] 93321_cadecay.mod (2500), 93321_cas.mod (2501), 93321_cat.mod (2502), 93321_dc.mod (2503), 93321_fast.mod (2504), 93321_g_a.mod (2505), 93321_g_cas.mod (2506), 93321_g_cat.mod (2507), 93321_g_h.mod (2508), 93321_g_kca.mod (2509), 93321_g_kd.mod (2510), 93321_g_na.mod (2511), 93321_h.mod (2512), 93321_ka.mod (2513), 93321_kca.mod (2514), 93321_kd.mod (2515), 93321_na.mod (2516), 93321_slow.mod (2517)

[Calin-Jageman et al., 2007] 93325_leak.mod (2518), 93325_shunt.mod (2519), 93325_thold.mod (2520), 93325_tholdsyn.mod (2521)

[Getting, 1983] 93326_leak.mod (2522), 93326_oldtsyn.mod (2523), 93326_shunt.mod (2524), 93326_thold.mod (2525)

[Cali et al., 2008] 94321_Isin.mod (2526), 94321_Izap.mod (2527), 94321_hh2.mod (2528)

[Barnes and Hille, 1989] 95870_CPR.mod (2529), 95870_Ca.mod (2530), 95870_Cad.mod (2531), 95870_IinjLT.mod (2532), 95870_Kv.mod (2533), 95870_Kx.mod (2534), 95870_Leak.mod (2535), 95870_hyper.mod (2536)

[Schmidt-Hieber et al., 2007] 95960_asin.mod (2537), 95960_spines.mod (2538), 95960_synampa.mod (2539)

[Hines and Carnevale, 2008] 96444_exp2sid.mod (2540), 96444_expsid.mod (2541)

[Migliore and Shepherd, 2008] 97263_kamt.mod (2542), 97263_kdrmt.mod (2543), 97263_naxn.mod (2544), 97263_nmdanetOB.mod (2545)

[Poolos et al., 2002] 9769_h.mod (2546), 9769_kadist.mod (2547), 9769_kaprox.mod (2548), 9769_kdrca1.mod (2549), 9769_na3n.mod (2550), 9769_naxn.mod (2551), 9769_netstimm.mod (2552)

[Gurkiewicz and Korngreen, 2007] 97756_KChannel.mod (2553), 97756_svclmp.mod (2554)

[Chan et al., 2007] 97860_Ca.mod (2555), 97860_CaDiffuse2.mod (2556), 97860_CaDiffuse3.mod (2557), 97860_CaHandler2.mod (2558), 97860_bk_dop.mod (2559), 97860_cal_dop.mod (2560), 97860_cap_dop.mod (2561), 97860_hcn_siegelbaum.mod (2562), 97860_kcnq_hh.mod (2563), 97860_kir2_dop.mod (2564), 97860_kv1_gp.mod (2565), 97860_kv2_hh.mod (2566), 97860_kv2_simplehh.mod (2567), 97860_kv4hh.mod (2568), 97860_leak_dop.mod (2569), 97860_nahh.mod (2570), 97860_sk_dop.mod (2571), 97860_vramp.mod (2572)

[Beeler and Reuter, 1977] 97863_cadynam.mod (2573), 97863_ik1.mod (2574), 97863_ina.mod (2575), 97863_is.mod (2576), 97863_ix1.mod (2577), 97863_kcum.mod (2578), 97863_nacum.mod (2579), 97863_nipace.mod (2580)

[Lytton, 2006] 97874_exp2i.mod (2585), 97874_h.mod (2586), 97874_kadist.mod (2587), 97874_kaprox.mod (2588), 97874_kdrca1.mod (2589), 97874_na3s.mod (2590), 97874_naxn.mod (2591), 97874_netstims.mod (2592), 97874_nmdanet.mod (2593), 97874_vecst.mod (2594), 97874_vecst_v2.mod (2595)

[Hines et al., 2008] 97917_ka.mod (2616), 97917_ka_ib.mod (2617)

[Maurice et al., 2004] 98005_bk_ch.mod (2641), 98005_ca_ch.mod (2642), 98005_cal_ch.mod (2643), 98005_cap_ch.mod (2644), 98005_hcn12_ch.mod (2645), 98005_hcn2_ch.mod (2646), 98005_kcnq_ch.mod (2647), 98005_kir2_ch.mod (2648), 98005_kv2_ch.mod (2649), 98005_kv4_ch.mod (2650), 98005_na2_ch.mod (2651), 98005_na_ch.mod (2652), 98005_sk_ch.mod (2653), 98005_synampa.mod (2654)

[Edin et al., 2007] 98017_Cai.mod (2655), 98017_ICa.mod (2656), 98017_ICan.mod (2657), 98017_IKa.mod (2658), 98017_IKs.mod (2659), 98017_INap.mod (2660), 98017_MyNetStim.mod (2661), 98017_ampa.mod (2662), 98017_gabaR.mod (2663), 98017_gapJ.mod (2664), 98017_hhI.mod (2665), 98017_hhPyr.mod (2666), 98017_nmdaR.mod (2667), 98017_nmdaR_2.mod (2668), 98017_nmdaSUM.mod (2669), 98017_nmdaSUM_2.mod (2670)

[Stockbridge and Moore, 1984] 9888_stock.mod (2671)

[Lytton et al., 1997] 9889_AMPA.mod (2672), 9889_GABAB1.mod (2673), 9889_GABALOW.mod (2674), 9889_HH_traub.mod (2675), 9889_IAHP_destexhe.mod (2676), 9889_ICAN_destexhe.mod (2677), 9889_IT2_huguenard.mod (2678), 9889_IT_wang.mod (2679), 9889_Ih_old.mod (2680), 9889_calciumpump_destexhe.mod (2681), 9889_gen.mod (2682), 9889_nmda.mod (2683), 9889_passiv.mod (2684), 9889_presyn.mod (2685), 9889_pulse.mod (2686)

[Banitt et al., 2007]

[Anderson et al., 1994b]

[Anderson et al., 1994a]

[Banitt et al., 2005] 114637_VectorSynNS.mod (2696), 114637_iA.mod (2697), 114637_iap.mod (2698), 114637_ic.mod (2699), 114637_ical.mod (2700)

[Helmstaedter et al., 2009c] 116053_ca.mod (2701), 116053_cad.mod (2702), 116053_kca.mod (2703), 116053_km.mod (2704), 116053_kv.mod (2705), 116053_na.mod (2706)

[Helmstaedter et al., 2009b] 116053_ca.mod (2701), 116053_cad.mod (2702), 116053_kca.mod (2703), 116053_km.mod (2704), 116053_kv.mod (2705), 116053_na.mod (2706)

[Helmstaedter et al., 2009a] 116053_ca.mod (2701), 116053_cad.mod (2702), 116053_kca.mod (2703), 116053_km.mod (2704), 116053_kv.mod (2705), 116053_na.mod (2706)

[Helmstaedter et al., 2008] 116053_ca.mod (2701), 116053_cad.mod (2702), 116053_kca.mod (2703), 116053_km.mod (2704), 116053_kv.mod (2705), 116053_na.mod (2706)

[Brette et al., 2008] 114643_PcIpulse.mod (2707), 114643_PeCompRB.mod (2708), 114643_PeFitRB.mod (2709), 114643_PpGnoise.mod (2710), 114643_PpGpulse.mod (2711), 114643_PpInoise.mod (2712), 114643_PpIpulse.mod (2713)

[Jarsky et al., 2005] 116084_kadist.mod (2714), 116084_kaprox.mod (2715), 116084_kdrca1.mod (2716), 116084_na3.mod (2717), 116084_nax.mod (2718), 116084_vmax.mod (2719)

[Hao et al., 2009] 127305_ampa.mod (2728), 127305_gabaa.mod (2729), 127305_gabab.mod (2730), 127305_h.mod (2731), 127305_kadist.mod (2732), 127305_kaprox.mod (2733), 127305_kdrca1.mod (2734), 127305_na3.mod (2735), 127305_nax.mod (2736), 127305_nmda.mod (2737), 127305_shunting_factor.mod (2738)

[Katz et al., 2009] 127351_conductance.mod (2739), 127351_dv_yael.mod (2740), 127351_id.mod (2741), 127351_kad.mod (2742), 127351_kap.mod (2743), 127351_kdr.mod (2744), 127351_nax.mod (2745)

[Orio et al., 2012] 141063_dr.mod (2746), 141063_ih.mod (2747), 141063_leak.mod (2748), 141063_ou.mod (2749), 141063_sdsr.mod (2750), 141063_trek1.mod (2751)

[Chadderdon et al., 2012] 144538_drspk.mod (2752), 144538_infot.mod (2753), 144538_intf6_.mod (2754), 144538_intfsw.mod (2755), 144538_misc.mod (2756), 144538_nstim.mod (2757), 144538_stats.mod (2758), 144538_updown.mod (2759), 144538_vecst.mod (2760)

[Amatrudo et al., 2012] 144553_kvz_nature.mod (2761), 144553_naz_nature.mod (2762), 144553_vsource.mod (2763)

[Clopath et al., 2010] 144566_stdp_cc.mod (2764)

[Chambers et al., 2012] 144579_alphasyndiffeq.mod (2765), 144579_alphasynkin.mod (2766), 144579_alphasynkint.mod (2767), 144579_ampa.mod (2768), 144579_ar.mod (2769), 144579_cad.mod (2770), 144579_cal.mod (2771), 144579_cat.mod (2772), 144579_cat_a.mod (2773), 144579_gabaa.mod (2774), 144579_iclamp_const.mod (2775), 144579_k2.mod (2776), 144579_ka.mod (2777), 144579_ka_ib.mod (2778), 144579_kahp.mod (2779), 144579_kahp_deeppyr.mod (2780), 144579_kahp_slower.mod (2781), 144579_kc.mod (2782), 144579_kc_fast.mod (2783), 144579_kdr.mod (2784), 144579_kdr_fs.mod (2785), 144579_km.mod (2786), 144579_naf.mod (2787), 144579_naf2.mod (2788), 144579_naf_tcr.mod (2789), 144579_nap.mod (2790), 144579_napf.mod (2791), 144579_napf_spinstell.mod (2792), 144579_napf_tcr.mod (2793), 144579_par_ggap.mod (2794), 144579_pulsesyn.mod (2795), 144579_rampsyn.mod (2796), 144579_rand.mod (2797), 144579_ri.mod (2798), 144579_traub_nmda.mod (2799)

[Mai and Liu, 2009] 144586_bnet.mod (2800), 144586_misc.mod (2801), 144586_stats.mod (2802), 144586_vecst.mod (2803)

[Culmone and Migliore, 2012] 144976_distr.mod (2804), 144976_h.mod (2805), 144976_kadist.mod (2806), 144976_kaprox.mod (2807), 144976_kdrca1.mod (2808), 144976_na3n.mod (2809), 144976_naxn.mod (2810), 144976_netstims.mod (2811)

[Fineberg et al., 2012] 145672_Kv4_csi.mod (2812), 145672_Kv4_csiosi.mod (2813), 145672_migliore_kaprox.mod (2814), 145672_migliore_kdrca1.mo (2815), 145672_migliore_na3.mod (2816), 145672_migliore_nax.mod (2817)

[Moradi et al., 2013] 145836_SynExp2NMDA.mod (2818), 145836_SynExp3NMDA.mod (2819), 145836_SynExp3NMDA2.mod (2820), 145836_SynExp4NMDA.mod (2821), 145836_SynExp4NMDA2.mod (2822), 145836_SynExp5NMDA.mod (2823), 145836_SynExp5NMDA2.mod (2824), 145836_SynExp5NMDA3.mod (2825), 145836_SynNMDA10_1.mod (2826), 145836_SynNMDA10_2.mod (2827), 145836_SynNMDA10_2_2.mod (2828), 145836_SynNMDA16.mod (2829), 145836_SynNMDA16_2.mod (2830)

[Bahl et al., 2012] 146026_IKM.mod (2831), 146026_SlowCa.mod (2832), 146026_cad.mod (2833), 146026_h.mod (2834), 146026_kca.mod (2835), 146026_kfast.mod (2836), 146026_kslow.mod (2837), 146026_nap.mod (2838), 146026_nat.mod (2839)

[O'Connor et al., 2012] 146030_AMPA.mod (2840), 146030_Ca_mit_conc_ChannelML.mod (2841), 146030_CurrentClampExt.mod (2842), 146030_KA_ChannelML.mod (2843), 146030_KCa3_ChannelML_new.mod (2844), 146030_Kdr_ChannelML.mod (2845), 146030_LCa3_mit_usb_ChannelM (2846), 146030_LeakConductance.mod (2847), 146030_NaxSH0_ChannelML.mod (2848), 146030_NaxSH10_ChannelML.mod (2849), 146030_SynForRnd (2850)

[Marasco et al., 2012] 146376_h.mod (2851), 146376_kadist.mod (2852), 146376_kaprox.mod (2853), 146376_kdrca1.mod (2854), 146376_na3n.mod (2855), 146376_naxn.mod (2856), 146376_distr.mod (2857)

[Lavzin et al., 2012] 146565_CaT.mod (2858), 146565_ampa.mod (2859), 146565_ca.mod (2860), 146565_cad_long.mod (2861), 146565_cad_old.mod (2862), 146565_gaba.mod (2863), 146565_glutamate.mod (2864), 146565_h.mod (2865), 146565_hh3.mod (2866), 146565_kca.mod (2867), 146565_netgaba.mod (2868), 146565_netglutamate.mod (2869), 146565_hh3_icgK2.mod (3482)

[Traub et al., 2005a]

[Traub et al., 2005b] 45539_alphasyndiffeq.mod (2883), 45539_alphasynkin.mod (2884), 45539_alphasynkint.mod (2885), 45539_ampa.mod (2886), 45539_ar.mod (2887), 45539_cad.mod (2888), 45539_cal.mod (2889), 45539_cat.mod (2890), 45539_cat_a.mod (2891), 45539_gabaa.mod (2892), 45539_iclamp_const.mod (2893), 45539_k2.mod (2894), 45539_ka.mod (2895), 45539_ka_ib.mod (2896), 45539_kahp.mod (2897), 45539_kahp_deeppyr.mod (2898), 45539_kahp_slower.mod (2899), 45539_kc.mod (2900), 45539_kc_fast.mod (2901), 45539_kdr.mod (2902), 45539_kdr_fs.mod (2903), 45539_km.mod (2904), 45539_naf.mod (2905), 45539_naf2.mod (2906), 45539_naf_tcr.mod (2907), 45539_nap.mod (2908), 45539_napf.mod (2909), 45539_napf_spinstell.mod (2910), 45539_napf_tcr.mod (2911), 45539_par_ggap.mod (2912), 45539_pulsesyn.mod (2913), 45539_rampsyn.mod (2914), 45539_rand.mod (2915), 45539_ri.mod (2916), 45539_traub_nmda.mod (2917)

[Golding et al., 2001] 64167_kadist.mod (2918), 64167_kaprox.mod (2919), 64167_kdrca1.mod (2920), 64167_nax.mod (2921), 64167_vmax.mod (2922), 64167_vmax2.mod (2923)

[Golding et al., 2005] 64170_dv.mod (2924), 64170_ih.mod (2925), 64170_spines.mod (2926), 64170_synampa.mod (2927)

[Schaefer et al., 2003b] 83344_cad2.mod (2928), 83344_child.mod (2929), 83344_childa.mod (2930), 83344_epsp.mod (2931), 83344_it2.mod (2932), 83344_kaprox.mod (2933), 83344_kca.mod (2934), 83344_km.mod (2935), 83344_kv.mod (2936), 83344_na.mod (2937), 83344_SlowCa.mod (2938)

[Migliore et al., 2014] 151681_distrt.mod (2939), 151681_fi.mod (2940), 151681_kamt.mod (2941), 151681_kdrmt.mod (2942), 151681_naxn.mod (2943), 151681_ampanmda.mod (2944), 151681_ThreshDetect.mod (2945), 149415_kdrmt.mod (3323)

[Kim et al., 2013] 150288_ca.mod (2946), 150288_cal2.mod (2947), 150288_h.mod (2948), 150288_im.mod (2949), 150288_kadist.mod (2950), 150288_kaprox.mod (2951), 150288_kdrca1.mod (2952), 150288_kdrca1DA.mod (2953), 150288_kdrinter.mod (2954), 150288_leak.mod (2955), 150288_leakDA.mod (2956), 150288_leakinter.mod (2957), 150288_na3.mod (2958), 150288_na3DA.mod (2959), 150288_nainter.mod (2960), 150288_cadyn.mod (2961), 150288_capool.mod (2962), 150288_sahp.mod (2963), 150288_sahpNE.mod (2964)

[Behuret et al., 2013] 150240_hh2.mod (2965), 150240_IM.mod (2966), 150240_IT.mod (2967), 150240_ITGHK.mod (2968), 150240_cadecay.mod (2969)

[Li and Cleland, 2013] 149739_cadecay.mod (2970), 149739_cadecay2.mod (2971), 149739_Caint.mod (2972), 149739_Can.mod (2973), 149739_CaPN.mod (2974), 149739_CaT.mod (2975), 149739_hpg.mod (2976), 149739_kAmt.mod (2977), 149739_KCa.mod (2978), 149739_KDRmt.mod (2979), 149739_kfasttab.mod (2980), 149739_kM.mod (2981), 149739_KS.mod (2982), 149739_kslowtab.mod (2983), 149739_LCa.mod (2984), 149739_nafast.mod (2985), 149739_NaP.mod (2986), 149739_Naxn.mod (2987), 149739_Nicotin.mod (3324)

[van der Velden et al., 2012] 147514_cad.mod (2988), 147514_cadif.mod (2989), 147514_cadif_pump.mod (2990), 147514_kca.mod (2991), 147514_km.mod (2992), 147514_kv.mod (2993), 147514_L_HVA_Ca.mod (2994), 147514_na.mod (2995), 147514_ca.mod (2996)

[Romani et al., 2013] 147757_cad.mod (2997), 147757_cagk.mod (2998), 147757_cal.mod (2999), 147757_calH.mod (3000), 147757_car.mod (3001), 147757_cat.mod (3002), 147757_h.mod (3003), 147757_kadist.mod (3004), 147757_kaprox.mod (3005), 147757_kca.mod (3006), 147757_kdr.mod (3007), 147757_km.mod (3008), 147757_na3.mod (3009), 147757_na3dend.mod (3010), 147757_na3notrunk.mod (3011), 147757_nap.mod (3012), 147757_nax.mod (3013), 147757_somacar.mod (3014)

[Narayanan and Johnston, 2010] 147538_h.mod (3015), 147538_kadist.mod (3016), 147538_kdrca1.mod (3017), 147538_na3s.mod (3018), 147538_cad.mod (3019)

[Miceli et al., 2013] 148094_cad.mod (3020), 148094_cagk.mod (3021), 148094_cal2.mod (3022), 148094_cat.mod (3023), 148094_h.mod (3024), 148094_kadist.mod (3025), 148094_KahpM95.mod (3026), 148094_kaprox.mod (3027), 148094_kdrca1.mod (3028), 148094_kv72wt73wt72R213Q. (3029), 148094_kv72wt73wt72R213W.mod (3030), 148094_na3n.mod (3031), 148094_naxn.mod (3032), 148094_cacumm.mod (3033), 148094_kv72wt73w (3322)

[Nakano et al., 2013] 151458_bkkca.mod (3034), 151458_cadyn.mod (3035), 151458_caL.mod (3036), 151458_caL13.mod (3037), 151458_caldyn.mod (3038), 151458_can.mod (3039), 151458_caq.mod (3040), 151458_car.mod (3041), 151458_cat.mod (3042), 151458_kaf.mod (3043), 151458_kas.mod (3044), 151458_kir.mod (3045), 151458_krp.mod (3046), 151458_naf.mod (3047), 151458_nap.mod (3048), 151458_skkca.mod (3049)

[Ashhad and Narayanan, 2013] 150551_cat.mod (3050), 150551_kadist.mod (3051), 150551_kaprox.mod (3052), 150551_kdrca1.mod (3053), 150551_na3.mod (3054), 150551_nax.mod (3055), 150551_cal4.mod (3056), 150551_caltype.mod (3057)

[Narayanan and Johnston, 2007] 147578_h.mod (3058), 147539_h.mod (3260)

[Chiu et al., 2013] 143604_ca.mod (3059), 143604_cad.mod (3060), 143604_ca_a1g.mod (3061), 143604_ca_a1h.mod (3062), 143604_constant.mod (3063), 143604_im.mod (3064), 143604_kca.mod (3065), 143604_km.mod (3066), 143604_kv.mod (3067), 143604_na.mod (3068)

[Miocinovic et al., 2006] 151460_Cacum.mod (3069), 151460_CaT.mod (3070), 151460_HVA.mod (3071), 151460_Ih.mod (3072), 151460_KDR.mod (3073), 151460_Kv31.mod (3074), 151460_Na.mod (3075), 151460_NaL.mod (3076), 151460_PARAK75.mod (3077), 151460_AXNODE75.mod (3078), 151460_sKCa.mod (3079), 151460_STh.mod (3080), 147218_cap.mod (3081), 147218_gksub.mod (3082), 147218_kdr.mod (3083), 147218_leak.mod (3084), 147218_pf.mod (3085), 147218_pf_phasic.mod (3086), 147218_rsg.mod (3087), 147218_cf.mod (3088), 147218_calcium.mod (3089), 147218_stellat.mod (3090), 147218_stellat_phasic.mod (3091), 151460_AXNODE75_icgNA2.mod (3458)

[Sousa et al., 2014] 151949_CaIntraCellDyn.mod (3092), 151949_HH2.mod (3093), 151949_iCaAN.mod (3094), 151949_iCaL.mod (3095), 151949_iKCa.mod (3096)

[Jedlicka et al., 2011] 148253_cldif2.mod (3097), 148253_cldif.mod (3098)

[Bianchi et al., 2014] 151126_bgka.mod (3099), 151126_cad.mod (3100), 151126_cagk.mod (3101), 151126_cal.mod (3102), 151126_calH.mod (3103), 151126_car.mod (3104), 151126_cat.mod (3105), 151126_ccanl.mod (3106), 151126_gskch.mod (3107), 151126_h.mod (3108), 151126_IA.mod (3109), 151126_ichan2.mod (3110), 151126_Ih.mod (3111), 151126_kadist.mod (3112), 151126_kaprox.mod (3113), 151126_Kaxon.mod (3114), 151126_kca.mod (3115), 151126_Kdend.mod (3116), 151126_kdr.mod (3117), 151126_kdrax.mod (3118), 151126_km.mod (3119), 151126_Ksoma.mod (3120), 151126_LcaMig.mod (3121), 151126_na3.mod (3122), 151126_na3dend.mod (3123), 151126_na3notrunk.mod (3124), 151126_Naaxon.mod (3125), 151126_Nadend.mod (3126), 151126_nap.mod (3127), 151126_Nasoma.mod (3128), 151126_nax.mod (3129), 151126_nca.mod (3130), 151126_somacar.mod (3131)

[Behabadi et al., 2012] 151404_hh3k.mod (3132), 151404_hh3na.mod (3133)

[Ferrante et al., 2013] 146509_kadist.mod (3134), 146509_kaprox.mod (3135), 146509_kdrca1.mod (3136), 146509_na3n.mod (3137), 146509_h.mod (3138)

[Neymotin et al., 2013] 151282_CA3ih.mod (3139), 151282_CA3ika.mod (3140), 151282_CA3ina.mod (3141), 151282_caolmw.mod (3142), 151282_HCN1.mod (3143), 151282_icaolmw.mod (3144), 151282_iholmw.mod (3145), 151282_ihstatic.mod (3146), 151282_kcaolmw.mod (3147), 151282_kdrbwb.mod (3148), 151282_nafbwb.mod (3149), 151282_CA3ikdr.mod (3325), 151282_CA3ika_icgK2.mod (3462)

[Narayanan and Chattarji, 2010] 147867_cadiv.mod (3150), 147867_cagk.mod (3151), 147867_cal2.mod (3152), 147867_can2.mod (3153), 147867_cat.mod (3154), 147867_h.mod (3155), 147867_kad.mod (3156), 147867_kahp.mod (3157), 147867_kap.mod (3158), 147867_kdr.mod (3159), 147867_nahh.mod (3160), 147867_borgkm.mod (3161)

[Torben-Nielsen et al., 2012] 144502_kdr.mod (3162), 144502_leak.mod (3163), 144502_na.mod (3164), 144502_stoca.mod (3165)

[Almog and Korngreen, 2014] 151825_BK.mod (3166), 151825_cad.mod (3167), 151825_ca_h.mod (3168), 151825_ca_r.mod (3169), 151825_ih.mod (3170), 151825_kfast.mod (3171), 151825_kslow.mod (3172), 151825_na.mod (3173), 151825_SK.mod (3174)

[Ovsepian et al., 2013] 150024_CaHVA.mod (3175), 150024_CalConc.mod (3176), 150024_CaLVA.mod (3177), 150024_fKdr.mod (3178), 150024_h.mod (3179), 150024_NaF.mod (3180), 150024_NaP.mod (3181), 150024_pasDCN.mod (3182), 150024_CaConc.mod (3183), 150024_SK.mod (3184), 150024_sKdr.mod (3185), 150024_TNC.mod (3186)

[Yu et al., 2013b] 144570_kamt.mod (3187), 144570_kdrmt.mod (3188), 144570_naxn.mod (3189)

[Schmidt-Hieber and Hausser, 2013] 150239_hcn.mod (3190), 150239_kap.mod (3191), 150239_kdr.mod (3192), 150239_Nap.mod (3193), 150239_nax.mod (3194)

[Wang et al., 2013] 149737_ih_stochastic.mod (3195), 149737_kfast.mod (3196), 149737_kslow.mod (3197), 149737_na.mod (3198)

[Berzhanskaya et al., 2013] 144589_h.mod (3199), 144589_kadist.mod (3200), 144589_kaprox.mod (3201), 144589_kdrca1.mod (3202), 144589_na3.mod (3203), 144589_nax.mod (3204)

[Mattioni and Le Novere, 2013] 150284_bkkca.mod (3205), 150284_cadyn.mod (3206), 150284_caL.mod (3207), 150284_caL13.mod (3208), 150284_caldyn.mod (3209), 150284_caltrack.mod (3210), 150284_can.mod (3211), 150284_caq.mod (3212), 150284_car.mod (3213), 150284_cat.mod (3214), 150284_catrack.mod (3215), 150284_kaf.mod (3216), 150284_kas.mod (3217), 150284_kir.mod (3218), 150284_krp.mod (3219), 150284_naf.mod (3220), 150284_nap.mod (3221), 150284_rubin.mod (3222), 150284_skkca.mod (3223)

[Parekh and Ascoli, 2013] 148644_h.mod (3224), 148644_kadist.mod (3225), 148644_kaprox.mod (3226), 148644_kdrca1.mod (3227), 148644_na3n.mod (3228), 148644_naxn.mod (3229), 153280_ch_CavL.mod (3394), 153280_ch_CavLZ.mod (3395), 153280_ch_CavN.mod (3396), 153280_ch_CavT.mod (3397), 153280_ch_HCNp.mod (3398), 153280_ch_KCaS.mod (3399)

[Migliore and McTavish, 2013] 149415_kamt.mod (3230), 149415_naxn.mod (3231), 150556_KAfm.mod (3261), 150556_KAsm.mod (3262), 150556_Kirm.mod (3263), 150556_Km.mod (3264), 150556_Krpm.mod (3265), 150556_Leakm.mod (3266), 150556_Nam.mod (3267), 150556_NaPm.mod (3268), 150556_NaSm.mod (3269)

[Foutz et al., 2012] 153196_chanrhod.mod (3232), 153196_kca.mod (3233), 153196_km.mod (3234), 153196_kv.mod (3235), 153196_na.mod (3236), 153196_na12.mod (3237), 153196_na16.mod (3238), 153196_ca.mod (3239), 153196_cad.mod (3240)

[Hay et al., 2013] 149100_Ca_LVAst.mod (3241), 149100_Ih.mod (3242), 149100_K_Pst.mod (3243), 149100_K_Tst.mod (3244), 149100_Nap_Et2.mod (3245), 149100_NaTg.mod (3246), 149100_CaDynamics_E2.mod (3247), 149100_Ca_HVA.mod (3248), 149100_SKv3_1.mod (3249), 149100_SK_E2.mod (3250)

[Balbi et al., 2014] 151443_Kdr.mod (3251), 151443_K_No.mod (3252), 151443_lk.mod (3253), 151443_mAHP.mod (3254), 151443_Naf_IS.mod (3255), 151443_Naf_No.mod (3256), 151443_Naf_So.mod (3257), 151443_Nap.mod (3258), 151443_Nap_No.mod (3259)

[Thomas and Petrou, 2013] 149174_bgka.mod (3270), 149174_CaBK.mod (3271), 149174_ccanl.mod (3272), 149174_gskch.mod (3273), 149174_hyperde3.mod (3274), 149174_ichan2.mod (3275), 149174_LcaMig.mod (3276), 149174_nad.mod (3277), 149174_nca.mod (3278), 149174_tca.mod (3279), 149174_hyperde3_icgIH2.mod (3473), 149174_ichan2_icgK2.mod (3495)

[Conde-Sousa and Aguiar, 2013] 147461_HH2.mod (3280), 147461_iCaAN.mod (3281), 147461_iCaL.mod (3282), 147461_CaIntraCellDyn.mod (3283)

[Mahon et al., 2000] 150621_KAfm.mod (3284), 150621_KAsm.mod (3285), 150621_Kirm.mod (3286), 150621_Km.mod (3287), 150621_Krpm.mod (3288), 150621_Leakm.mod (3289), 150621_Nam.mod (3290), 150621_NaPm.mod (3291), 150621_NaSm.mod (3292)

[Vladimirov et al., 2013] 150446_KahpTraub1994.mod (3293), 150446_KaTraub1994.mod (3294), 150446_KcTraub1994.mod (3295), 150446_KdrTraub1994ax.mod (3296), 150446_KdrTraub1994sd.mod (3297), 150446_NafTraub1994ax.mod (3298), 150446_NafTraub1994sd.mod (3299), 150446_CadTraub1994.mod (3300), 150446_CaTraub1994.mod (3301)

[Marasco et al., 2013] 149000_CalciumP.mod (3302), 149000_CaP.mod (3303), 149000_CaP2.mod (3304), 149000_CaT.mod (3305), 149000_K2.mod (3306), 149000_K22.mod (3307), 149000_K23.mod (3308), 149000_KA.mod (3309), 149000_KC.mod (3310), 149000_KC2.mod (3311), 149000_KC3.mod (3312), 149000_KD.mod (3313), 149000_Kdr.mod (3314), 149000_Kh.mod (3315), 149000_Khh.mod (3316), 149000_KM.mod (3317), 149000_Leak.mod (3318), 149000_NaF.mod (3319), 149000_NaP.mod (3320), 149000_CaE.mod (3321), 148094_kv72wt73wt.mod (3322)

[Avella Gonzalez et al., 2014] 155705_K_pyr_bk.mod (3326), 155705_Na_pyr_bk.mod (3327)

[Avella Gonzalez et al., 2012] 156120_hh1.mod (3328), 156120_h_chann.mod (3329), 156120_km.mod (3330), 156120_K_pyr_bk.mod (3331), 156120_Na_pyr_bk.mod (3332)

[Proddutur et al., 2013] 155601_ccanl.mod (3333), 155601_gskch.mod (3334), 155601_hyperde3.mod (3335), 155601_ichan2.mod (3336), 155601_LcaMig.mod (3337), 155601_markov.mod (3338), 155601_nca.mod (3339), 155601_bgka.mod (3340), 155601_CaBK.mod (3341), 155601_tca.mod (3342), 155601_ichan2_icgK2.mod (3452), 155601_hyperde3_icgIH2.mod (3464)

[Tejada et al., 2014] 155568_CaBK.mod (3343), 155568_ccanl.mod (3344), 155568_gskch.mod (3345), 155568_hyperde3.mod (3346), 155568_ichan2.mod (3347), 155568_LcaMig.mod (3348), 155568_nca.mod (3349), 155568_bgka.mod (3350), 155568_tca.mod (3351), 155568_ichan2_icgK2 (3451), 155568_hyperde3_icgIH2.mod (3466)

[Overstreet et al., 2013] 147460_AXNODE.mod (3352), 147460_AXNODE_icgK2.mod (3457), 147460_AXNODE_icgNA2.mod (3493)

[Chambers et al., 2014] 155796_can.mod (3353), 155796_cansc.mod (3354), 155796_ih.mod (3355), 155796_jcal.mod (3356), 155796_ka.mod (3357), 155796_kca_fast.mod (3358), 155796_kca_slow.mod (3359), 155796_kdr.mod (3360), 155796_nav13.mod (3361), 155796_nav17.mod (3362), 155796_nav19.mod (3363)

[Papoutsi et al., 2013] 155057_ampa.mod (3364), 155057_ampain.mod (3365), 155057_cadyn.mod (3366), 155057_cal.mod (3367), 155057_can.mod (3368), 155057_car.mod (3369), 155057_cat.mod (3370), 155057_gabaa.mod (3371), 155057_gabaain.mod (3372), 155057_gabab.mod (3373), 155057_h.mod (3374), 155057_ican.mod (3375), 155057_iks.mod (3376), 155057_kadist.mod (3377), 155057_kca.mod (3378), 155057_kct.mod (3379), 155057_kdr.mod (3380), 155057_naf.mod (3381), 155057_nap.mod (3382), 155057_NMDA.mod (3383), 155057_NMDAsyn.mod (3384)

[Li et al., 2013] 150538_cad2.mod (3385), 150538_GABABsyn.mod (3386), 150538_h.mod (3387), 150538_kca.mod (3388), 150538_km.mod (3389), 150538_kv.mod (3390), 150538_na.mod (3391), 150538_NMDAr.mod (3392), 150538_SlowCa.mod (3393)

[Lee et al., 2014] 153280_ch_CavL.mod (3394), 153280_ch_CavLZ.mod (3395), 153280_ch_CavN.mod (3396), 153280_ch_CavT.mod (3397), 153280_ch_HCNp.mod (3398), 153280_ch_KCaS.mod (3399), 153280_ch_Kdrfast.mod (3400), 153280_ch_Kdrp.mod (3401), 153280_ch_Kdrslow.mod (3402), 153280_ch_KvA.mod (3403), 153280_ch_KvAdistp.mod (3404), 153280_ch_KvAproxp.mod (3405), 153280_ch_KvCaB.mod (3406), 153280_ch_leak.mod (3407), 153280_ch_Navp.mod (3408), 153280_ch_NavPVBC.mod (3409), 153280_iconc_Ca.mod (3410), 153280_iconc_CaZ.mod (3411)

[Kaplan and Lansner, 2014] 155157_AType_potassium.mod (3412), 155157_cadecay_destexhe.mod (3413), 155157_cadecay.mod (3414), 155157_cagk2.mod (3415), 155157_HH_traub.mod (3416), 155157_IL_gutnick.mod (3417), 155157_IM_cortex.mod (3418), 155157_IT_huguenard.mod (3419), 155157_kA.mod (3420), 155157_kca.mod (3421), 155157_kfasttab.mod (3422), 155157_kM.mod (3423), 155157_kslowtab.mod (3424), 155157_lcafixed.mod (3425), 155157_nafast.mod (3426), 155157_nagran.mod (3427)

[Graham et al., 2014] 154732_cagk.mod (3428), 154732_carF.mod (3429), 154732_h.mod (3430), 154732_kadist.mod (3431), 154732_kaprox.mod (3432), 154732_kca.mod (3433), 154732_kdrca1.mod (3434), 154732_km.mod (3435), 154732_na3n.mod (3436), 154732_naxn.mod (3437), 154732_cad.mod (3438)

[Yu et al., 2013a] 155602_ccanl.mod (3439), 155602_gskch.mod (3440), 155602_hyperde3.mod (3441), 155602_ichan2.mod (3442), 155602_LcaMig.mo (3443), 155602_markov.mod (3444), 155602_nca.mod (3445), 155602_bgka.mod (3446), 155602_CaBK.mod (3447), 155602_tca.mod (3448), 155602_ichan2_icgK2.mod (3453), 155602_hyperde3_icgIH2.mod (3465)

[Markaki et al., 2005] 119266_KdBG.mod (389), 119266_cad.mod (390), 119266_cadL.mod (391), 119266_cadN.mod (392), 119266_cal.mod (393), 119266_can.mod (394), 119266_car.mod (395), 119266_cat.mod (396), 119266_d3.mod (397), 119266_h.mod (398), 119266_hha2.mod (399), 119266_hha_old.mod (400), 119266_ic.mod (401), 119266_kadist.mod (402), 119266_kaprox.mod (403), 119266_kca.mod (404), 119266_km.mod (405), 119266_nap.mod (406)

[Kampa and Stuart, 2006] 108458_CaH.mod (73), 108458_CaT.mod (74), 108458_Cad.mod (75), 108458_Ka.mod (76), 108458_Kv.mod (77), 108458_na.mod (78)

[Lee, 2007] 125857_cacurrent.mod (660), 125857_cadecay.mod (661), 125857_kaf.mod (662), 125857_kahp.mod (663), 125857_kc.mod (664), 125857_kdr.mod (665), 125857_leak.mod (666), 125857_naf.mod (667), 125857_nap.mod (668)

[Weaver and Wearne, 2006] 87473_cad.mod (2481), 87473_cahi.mod (2482), 87473_feature_weaver.mod (2483), 87473_fn.mod (2484), 87473_ka.mod (2485), 87473_kca.mod (2486), 87473_nap.mod (2487)

[Saftenku, 2002] 51196_ampa.mod (2051), 51196_glu1.mod (2052), 51196_gr_ltp1.mod (2053), 51196_grc_ca.mod (2054), 51196_grc_calc.mod (2055), 51196_grc_ka.mod (2056), 51196_grc_kca.mod (2057), 51196_grc_kir.mod (2058), 51196_grc_lkg1.mod (2059), 51196_grc_lkg2.mod (2060), 51196_grc_pna.mod (2061), 51196_grg_km.mod (2062), 51196_grg_kv.mod (2063), 51196_grg_na.mod (2064), 51196_grg_nar.mod (2065), 51196_nmda.mod (2066), 51196_spgen2.mod (2067)

[Meuth et al., 2005] 121600_HH.mod (2720), 121600_ia.mod (2721), 121600_ic.mod (2722), 121600_ih.mod (2723), 121600_il.mod (2724), 121600_inap.mod (2725), 121600_it.mod (2726), 121600_leak.mod (2727)

[Huguenard et al., 1997] 3808_HH1.mod (1962), 3808_HH2.mod (1963), 3808_ampa.mod (1964), 3808_ampa2.mod (1965), 3808_cadyn.mod (1966), 3808_gabaA.mod (1967), 3808_gabaA2.mod (1968), 3808_gabaB.mod (1969), 3808_gabaB2.mod (1970), 3808_ia.mod (1971), 3808_iahp.mod (1972), 3808_iahp2.mod (1973), 3808_ic.mod (1974), 3808_ican.mod (1975), 3808_ih.mod (1976), 3808_il.mod (1977), 3808_im.mod (1978), 3808_it.mod (1979), 3808_it2.mod (1980), 3808_leak.mod (1981), 3808_nmda.mod (1982), 3808_nmda2.mod (1983), 3808_synstim.mod (1984)

[Simon et al., 1999] 3434_Counter.mod (1805), 3434_PoissonBinauralILDVM.mod (1806), 3434_PoissonBinauralVM.mod (1807), 3434_PoissonMon (1808), 3434_PoissonTrigSyn.mod (1809), 3434_SpikeStats.mod (1810), 3434_TrigSynCD.mod (1811), 3434_khh.mod (1812), 3434_khva.mod (1813), 3434_klva.mod (1814), 3434_nahh.mod (1815)

[Korogod et al., 1999] 20014_PasD.mod (1600), 20014_PasS.mod (1601), 20014_PasSA.mod (1602), 20014_hh1.mod (1603)

[Destexhe et al., 1994b] 18197_HH.mod (1516), 18197_gaba.mod (1517), 18197_glutamate.mod (1518)

[Graham et al., 2001] 19747_BKDNaDR.mod (1581), 19747_ampa.mod (1582), 19747_coh.mod (1583)

[Bahmer and Langner, 2006b] 87454_ih.mod (2477), 87454_kht.mod (2478), 87454_leak.mod (2479), 87454_na.mod (2480)

[Bahmer and Langner, 2006a] 87454_ih.mod (2477), 87454_kht.mod (2478), 87454_leak.mod (2479), 87454_na.mod (2480)

[Bahmer and Langner, 2009] 87454_ih.mod (2477), 87454_kht.mod (2478), 87454_leak.mod (2479), 87454_na.mod (2480)

[Bahmer and Langner, 2010] 87454_ih.mod (2477), 87454_kht.mod (2478), 87454_leak.mod (2479), 87454_na.mod (2480)

[Dodge and Cooley, 1973] 3805_dc.mod (1954)

Bibliography

References

[Acker and Antic, 2009] Acker, C. and Antic, S. (2009). Quantitative assessment of the distributions of membrane conductances involved in action potential backpropagation along basal dendrites. *Journal of neurophysiology*, 101(3):1524–41.

[Aguiar et al., 2010] Aguiar, P., Sousa, M., and Lima, D. (2010). Nmda channels together with l-type calcium currents and calcium-activated nonspecific cationic currents are sufficient to generate windup in wdr neurons. *Journal of neurophysiology*, 104(2):1155–66.

[Akemann and Knopfel, 2006] Akemann, W. and Knopfel, T. (2006). Interaction of kv3 potassium channels and resurgent sodium current influences the rate of spontaneous firing of purkinje neurons. *The Journal of neuroscience : the official journal of the Society for Neuroscience*, 26(17):4602–12.

[Akemann et al., 2009] Akemann, W., Lundby, A., Mutoh, H., and Knopfel, T. (2009). Effect of voltage sensitive fluorescent proteins on neuronal excitability. *Biophysical journal*, 96(10):3959–76.

[Alle et al., 2009] Alle, H., Roth, A., and Geiger, J. (2009). Energy-efficient action potentials in hippocampal mossy fibers. *Science (New York, N.Y.)*, 325(5946):1405–8.

[Almog and Korngreen, 2014] Almog, M. and Korngreen, A. (2014). A quantitative description of dendritic conductances and its application to dendritic excitation in layer 5 pyramidal neurons. *The Journal of neuroscience : the official journal of the Society for Neuroscience*, 34(1):182–96.

[Amatrudo et al., 2012] Amatrudo, J., Weaver, C., Crimins, J., Hof, P., Rosene, D., and Luebke, J. (2012). Influence of highly distinctive structural properties on the excitability of pyramidal neurons in monkey visual and prefrontal cortices. *The Journal of neuroscience : the official journal of the Society for Neuroscience*, 32(40):13644–60.

[Amir and Devor, 2003a] Amir, R. and Devor, M. (2003a). Electrical excitability of the soma of sensory neurons is required for spike invasion of the soma, but not for through-conduction. *Biophysical journal*, 84(4):2181–91.

[Amir and Devor, 2003b] Amir, R. and Devor, M. (2003b). Extra spike formation in sensory neurons and the disruption of afferent spike patterning. *Biophysical journal*, 84(4):2700–8.

[Anderson et al., 1999] Anderson, J., Binzegger, T., Kahana, O., Martin, K., and Segev, I. (1999). Dendritic asymmetry cannot account for directional responses of neurons in visual cortex. *Nature neuroscience*, 2(9):820–4.

[Anderson et al., 1994a] Anderson, J., Douglas, R., Martin, K., and Nelson, J. (1994a). Map of the synapses formed with the dendrites of spiny stellate neurons of cat visual cortex. *The Journal of comparative neurology*, 341(1):25–38.

[Anderson et al., 1994b] Anderson, J., Douglas, R., Martin, K., and Nelson, J. (1994b). Synaptic output of physiologically identified spiny stellate neurons in cat visual cortex. *The Journal of comparative neurology*, 341(1):16–24.

[Anwar et al., 2012] Anwar, H., Hong, S., and De Schutter, E. (2012). Controlling ca2+-activated k+ channels with models of ca2+ buffering in purkinje cells. *Cerebellum (London, England)*, 11(3):681–93.

[Aradi and Holmes, 1999] Aradi, I. and Holmes, W. (1999). Role of multiple calcium and calcium-dependent conductances in regulation of hippocampal dentate granule cell excitability. *Journal of computational neuroscience*, 6(3):215–35.

[Archie and Mel, 2000] Archie, K. and Mel, B. (2000). A model for intradendritic computation of binocular disparity. *Nature neuroscience*, 3(1):54–63.

[Arsiero et al., 2007] Arsiero, M., Luscher, H., Lundstrom, B., and Giugliano, M. (2007). The impact of input fluctuations on the frequency-current relationships of layer 5 pyramidal neurons in the rat medial prefrontal cortex. *The Journal of neuroscience : the official journal of the Society for Neuroscience*, 27(12):3274–84.

[Ascoli et al., 2010] Ascoli, G., Gasparini, S., Medinilla, V., and Migliore, M. (2010). Local control of postinhibitory rebound spiking in ca1 pyramidal neuron dendrites. *The Journal of neuroscience : the official journal of the Society for Neuroscience*, 30(18):6434–42.

[Ashhad and Narayanan, 2013] Ashhad, S. and Narayanan, R. (2013). Quantitative interactions between the a-type k+ current and inositol trisphosphate receptors regulate intraneuronal ca2+ waves and synaptic plasticity. *The Journal of physiology*, 591(Pt 7):1645–69.

[Aubie et al., 2012] Aubie, B., Sayegh, R., and Faure, P. (2012). Duration tuning across vertebrates. *The Journal of neuroscience : the official journal of the Society for Neuroscience*, 32(18):6373–90.

[Avella Gonzalez et al., 2014] Avella Gonzalez, O., van Aerde, K., Mansvelder, H., van Pelt, J., and van Ooyen, A. (2014). Inter-network interactions: impact of connections between oscillatory neuronal networks on oscillation frequency and pattern. *PloS one*, 9(7):e100899.

[Avella Gonzalez et al., 2012] Avella Gonzalez, O., van Aerde, K., van Elburg, R., Poil, S., Mansvelder, H., Linkenkaer-Hansen, K., van Pelt, J., and van Ooyen, A. (2012). External drive to inhibitory cells induces alternating episodes of high- and low-amplitude oscillations. *PLoS computational biology*, 8(8):e1002666.

[Baccus, 1998] Baccus, S. (1998). Synaptic facilitation by reflected action potentials: enhancement of transmission when nerve impulses reverse direction at axon branch points. *Proceedings of the National Academy of Sciences of the United States of America*, 95(14):8345–50.

[Badoual et al., 2006] Badoual, M., Zou, Q., Davison, A., Rudolph, M., Bal, T., Fregnac, Y., and Destexhe, A. (2006). Biophysical and phenomenological models of multiple spike interactions in spike-timing dependent plasticity. *International journal of neural systems*, 16(2):79–97.

[Bahl et al., 2012] Bahl, A., Stemmler, M., Herz, A., and Roth, A. (2012). Automated optimization of a reduced layer 5 pyramidal cell model based on experimental data. *Journal of neuroscience methods*, 210(1):22–34.

[Bahmer and Langner, 2006a] Bahmer, A. and Langner, G. (2006a). Oscillating neurons in the cochlear nucleus: I. experimental basis of a simulation paradigm. *Biological cybernetics*, 95(4):371–9.

[Bahmer and Langner, 2006b] Bahmer, A. and Langner, G. (2006b). Oscillating neurons in the cochlear nucleus: Ii. simulation results. *Biological cybernetics*, 95(4):381–92.

[Bahmer and Langner, 2009] Bahmer, A. and Langner, G. (2009). A simulation of chopper neurons in the cochlear nucleus with wideband input from onset neurons. *Biological cybernetics*, 100(1):21–33.

[Bahmer and Langner, 2010] Bahmer, A. and Langner, G. (2010). Parameters for a model of an oscillating neuronal network in the cochlear nucleus defined by genetic algorithms. *Biological cybernetics*, 102(1):81–93.

[Baker et al., 2011] Baker, J., Perez-Rosello, T., Migliore, M., Barrionuevo, G., and Ascoli, G. (2011). A computer model of unitary responses from associational/commissural and perforant path synapses in hippocampal ca3 pyramidal cells. *Journal of computational neuroscience*, 31(1):137–58.

[Baker, 2005] Baker, M. (2005). Protein kinase c mediates up-regulation of tetrodotoxin-resistant, persistent na+ current in rat and mouse sensory neurones. *The Journal of physiology*, 567(Pt 3):851–67.

[Bal and Oertel, 2000] Bal, R. and Oertel, D. (2000). Hyperpolarization-activated, mixed-cation current (i(h)) in octopus cells of the mammalian cochlear nucleus. *Journal of neurophysiology*, 84(2):806–17.

[Balbi et al., 2014] Balbi, P., Martinoia, S., Colombo, R., and Massobrio, P. (2014). Modelling recurrent discharge in the spinal alpha-motoneuron: reappraisal of the f wave. *Clinical neurophysiology : official journal of the International Federation of Clinical Neurophysiology*, 125(2):427–9.

[Banitt et al., 2005] Banitt, Y., Martin, K., and Segev, I. (2005). Depressed responses of facilitatory synapses. *Journal of neurophysiology*, 94(1):865–70.

[Banitt et al., 2007] Banitt, Y., Martin, K., and Segev, I. (2007). A biologically realistic model of contrast invariant orientation tuning by thalamocortical synaptic depression. *The Journal of neuroscience : the official journal of the Society for Neuroscience*, 27(38):10230–9.

[Bar-Yehuda and Korngreen, 2008] Bar-Yehuda, D. and Korngreen, A. (2008). Space-clamp problems when voltage clamping neurons expressing voltage-gated conductances. *Journal of neurophysiology*, 99(3):1127–36.

[Baranauskas and Martina, 2006] Baranauskas, G. and Martina, M. (2006). Sodium currents activate without a hodgkin-and-huxley-type delay in central mammalian neurons. *The Journal of neuroscience : the official journal of the Society for Neuroscience*, 26(2):671–84.

[Barela et al., 2006] Barela, A., Waddy, S., Lickfett, J., Hunter, J., Anido, A., Helmers, S., Goldin, A., and Escayg, A. (2006). An epilepsy mutation in the sodium channel scn1a that decreases channel excitability. *The Journal of neuroscience : the official journal of the Society for Neuroscience*, 26(10):2714–23.

[Barnes and Hille, 1989] Barnes, S. and Hille, B. (1989). Ionic channels of the inner segment of tiger salamander cone photoreceptors. *The Journal of general physiology*, 94(4):719–43.

[Bartos et al., 2001] Bartos, M., Vida, I., Frotscher, M., Geiger, J., and Jonas, P. (2001). Rapid signaling at inhibitory synapses in a dentate gyrus interneuron network. *The Journal of neuroscience : the official journal of the Society for Neuroscience*, 21(8):2687–98.

[Bartos et al., 2002] Bartos, M., Vida, I., Frotscher, M., Meyer, A., Monyer, H., Geiger, J., and Jonas, P. (2002). Fast synaptic inhibition promotes synchronized gamma oscillations in hippocampal interneuron networks. *Proceedings of the National Academy of Sciences of the United States of America*, 99(20):13222–7.

[Bazhenov et al., 1998] Bazhenov, M., Timofeev, I., Steriade, M., and Sejnowski, T. (1998). Computational models of thalamocortical augmenting responses. *The Journal of neuroscience : the official journal of the Society for Neuroscience*, 18(16):6444–65.

[Beck et al., 1992] Beck, H., Ficker, E., and Heinemann, U. (1992). Properties of two voltage-activated potassium currents in acutely isolated juvenile rat dentate gyrus granule cells. *Journal of neurophysiology*, 68(6):2086–99.

[Bedard et al., 2004] Bedard, C., Kroger, H., and Destexhe, A. (2004). Modeling extracellular field potentials and the frequency-filtering properties of extracellular space. *Biophysical journal*, 86(3):1829–42.

[Beeler and Reuter, 1977] Beeler, G. and Reuter, H. (1977). Reconstruction of the action potential of ventricular myocardial fibres. *The Journal of physiology*, 268(1):177–210.

[Behabadi et al., 2012] Behabadi, B., Polsky, A., Jadi, M., Schiller, J., and Mel, B. (2012). Location-dependent excitatory synaptic interactions in pyramidal neuron dendrites. *PLoS computational biology*, 8(7):e1002599.

[Behuret et al., 2013] Behuret, S., Deleuze, C., Gomez, L., Fregnac, Y., and Bal, T. (2013). Cortically-controlled population stochastic facilitation as a plausible substrate for guiding sensory transfer across the thalamic gateway. *PLoS computational biology*, 9(12):e1003401.

[Bellinger et al., 2008] Bellinger, S., Miyazawa, G., and Steinmetz, P. (2008). Submyelin potassium accumulation may functionally block subsets of local axons during deep brain stimulation: a modeling study. *Journal of neural engineering*, 5(3):263–74.

[Benison et al., 2001] Benison, G., Keizer, J., Chalupa, L., and Robinson, D. (2001). Modeling temporal behavior of postnatal cat retinal ganglion cells. *Journal of theoretical biology*, 210(2):187–99.

[Berzhanskaya et al., 2013] Berzhanskaya, J., Chernyy, N., Gluckman, B., Schiff, S., and Ascoli, G. (2013). Modulation of hippocampal rhythms by subthreshold electric fields and network topology. *Journal of computational neuroscience*, 34(3):369–89.

[Bhalla and Bower, 1993] Bhalla, U. and Bower, J. (1993). Exploring parameter space in detailed single neuron models: simulations of the mitral and granule cells of the olfactory bulb. *Journal of neurophysiology*, 69(6):1948–65.

[Bianchi et al., 2014] Bianchi, D., De Michele, P., Marchetti, C., Tirozzi, B., Cuomo, S., Marie, H., and Migliore, M. (2014). Effects of increasing creb-dependent transcription on the storage and recall processes in a hippocampal ca1 microcircuit. *Hippocampus*, 24(2):165–77.

[Bianchi et al., 2012] Bianchi, D., Marasco, A., Limongiello, A., Marchetti, C., Marie, H., Tirozzi, B., and Migliore, M. (2012). On the mechanisms underlying the depolarization block in the spiking dynamics of ca1 pyramidal neurons. *Journal of computational neuroscience*, 33(2):207–25.

[Bogaard et al., 2009] Bogaard, A., Parent, J., Zochowski, M., and Booth, V. (2009). Interaction of cellular and network mechanisms in spatiotemporal pattern formation in neuronal networks. *The Journal of neuroscience : the official journal of the Society for Neuroscience*, 29(6):1677–87.

[Borst and Haag, 1996] Borst, A. and Haag, J. (1996). The intrinsic electrophysiological characteristics of fly lobula plate tangential cells: I. passive membrane properties. *Journal of computational neuroscience*, 3(4):313–36.

[Botta et al., 2010] Botta, P., de Souza, F., Sangrey, T., De Schutter, E., and Valenzuela, C. (2010). Alcohol excites cerebellar golgi cells by inhibiting the na+/k+ atpase. *Neuropsychopharmacology : official publication of the American College of Neuropsychopharmacology*, 35(9):1984–96.

[Branco et al., 2010] Branco, T., Clark, B., and Hausser, M. (2010). Dendritic discrimination of temporal input sequences in cortical neurons. *Science (New York, N.Y.)*, 329(5999):1671–5.

[Branco and Hausser, 2011] Branco, T. and Hausser, M. (2011). Synaptic integration gradients in single cortical pyramidal cell dendrites. *Neuron*, 69(5):885–92.

[Brette et al., 2008] Brette, R., Piwkowska, Z., Monier, C., Rudolph-Lilith, M., Fournier, J., Levy, M., Fregnac, Y., Bal, T., and Destexhe, A. (2008). High-resolution intracellular recordings using a real-time computational model of the electrode. *Neuron*, 59(3):379–91.

[Brette et al., 2007] Brette, R., Rudolph, M., Carnevale, T., Hines, M., Beeman, D., Bower, J., Diesmann, M., Morrison, A., Goodman, P., Harris FC, J., Zirpe, M., Natschlager, T., Pecevski, D., Ermentrout, B., Djurfeldt, M., Lansner, A., Rochel, O., Vieville, T., Muller, E., Davison, A., El Boustani, S., and Destexhe, A. (2007). Simulation of networks of spiking neurons: a review of tools and strategies. *Journal of computational neuroscience*, 23(3):349–98.

[Brown et al., 2011] Brown, S., Moraru, I., Schaff, J., and Loew, L. (2011). Virtual neuron: a strategy for merged biochemical and electrophysiological modeling. *Journal of computational neuroscience*, 31(2):385–400.

[Bush et al., 1999] Bush, P., Prince, D., and Miller, K. (1999). Increased pyramidal excitability and nmda conductance can explain posttraumatic epileptogenesis without disinhibition: a model. *Journal of neurophysiology*, 82(4):1748–58.

[Cadetti and Belluzzi, 2001] Cadetti, L. and Belluzzi, O. (2001). Hyperpolarisation-activated current in glomerular cells of the rat olfactory bulb. *Neuroreport*, 12(14):3117–20.

[Cali et al., 2008] Cali, C., Berger, T., Pignatelli, M., Carleton, A., Markram, H., and Giugliano, M. (2008). Inferring connection proximity in networks of electrically coupled cells by subthreshold frequency response analysis. *Journal of computational neuroscience*, 24(3):330–45.

[Calin-Jageman et al., 2007] Calin-Jageman, R., Tunstall, M., Mensh, B., Katz, P., and Frost, W. (2007). Parameter space analysis suggests multi-site plasticity contributes to motor pattern initiation in tritonia. *Journal of neurophysiology*, 98(4):2382–98.

[Canavier, 1999] Canavier, C. (1999). Sodium dynamics underlying burst firing and putative mechanisms for the regulation of the firing pattern in midbrain dopamine neurons: a computational approach. *Journal of computational neuroscience*, 6(1):49–69.

[Canavier and Landry, 2006] Canavier, C. and Landry, R. (2006). An increase in ampa and a decrease in sk conductance increase burst firing by different mechanisms in a model of a dopamine neuron in vivo. *Journal of neurophysiology*, 96(5):2549–63.

[Carlin et al., 2000] Carlin, K., Jones, K., Jiang, Z., Jordan, L., and Brownstone, R. (2000). Dendritic l-type calcium currents in mouse spinal motoneurons: implications for bistability. *The European journal of neuroscience*, 12(5):1635–46.

[Carvalho and Buonomano, 2009] Carvalho, T. and Buonomano, D. (2009). Differential effects of excitatory and inhibitory plasticity on synaptically driven neuronal input-output functions. *Neuron*, 61(5):774–85.

[Casale and McCormick, 2011] Casale, A. and McCormick, D. (2011). Active action potential propagation but not initiation in thalamic interneuron dendrites. *The Journal of neuroscience : the official journal of the Society for Neuroscience*, 31(50):18289–302.

[Cassara et al., 2008] Cassara, A., Hagberg, G., Bianciardi, M., Migliore, M., and Maraviglia, B. (2008). Realistic simulations of neuronal activity: a contribution to the debate on direct detection of neuronal currents by mri. *NeuroImage*, 39(1):87–106.

[Cassara and Maraviglia, 2008] Cassara, A. and Maraviglia, B. (2008). Microscopic investigation of the resonant mechanism for the implementation of nc-mri at ultra-low field mri. *NeuroImage*, 41(4):1228–41.

[Chadderdon et al., 2012] Chadderdon, G., Neymotin, S., Kerr, C., and Lytton, W. (2012). Reinforcement learning of targeted movement in a spiking neuronal model of motor cortex. *PloS one*, 7(10):e47251.

[Chambers et al., 2012] Chambers, J., Bethwaite, B., Diamond, N., Peachey, T., Abramson, D., Petrou, S., and Thomas, E. (2012). Parametric computation predicts a multiplicative interaction between synaptic strength parameters that control gamma oscillations. *Frontiers in computational neuroscience*, 6:53.

[Chambers et al., 2014] Chambers, J., Bornstein, J., Gwynne, R., Koussoulas, K., and Thomas, E. (2014). A detailed, conductance-based computer model of intrinsic sensory neurons of the gastrointestinal tract. *American journal of physiology. Gastrointestinal and liver physiology*, 307(5):G517–32.

[Chan et al., 2007] Chan, C., Guzman, J., Ilijic, E., Mercer, J., Rick, C., Tkatch, T., Meredith, G., and Surmeier, D. (2007). 'rejuvenation' protects neurons in mouse models of parkinson's disease. *Nature*, 447(7148):1081–6.

[Chapman et al., 1983] Chapman, J., Johnson, E., and Kootsey, J. (1983). Electrical and biochemical properties of an enzyme model of the sodium pump. *The Journal of membrane biology*, 74(2):139–53.

[Chen et al., 2001a] Chen, K., Aradi, I., Thon, N., Eghbal-Ahmadi, M., Baram, T., and Soltesz, I. (2001a). Persistently modified h-channels after complex febrile seizures convert the seizure-induced enhancement of inhibition to hyperexcitability. *Nature medicine*, 7(3):331–7.

[Chen et al., 2001b] Chen, N., Ren, J., Raymond, L., and Murphy, T. (2001b). Changes in agonist concentration dependence that are a function of duration of exposure suggest n-methyl-d-aspartate receptor nonsaturation during synaptic stimulation. *Molecular pharmacology*, 59(2):212–9.

[Chen et al., 2002] Chen, W., Shen, G., Shepherd, G., Hines, M., and Midtgaard, J. (2002). Multiple modes of action potential initiation and propagation in mitral cell primary dendrite. *Journal of neurophysiology*, 88(5):2755–64.

[Chiu et al., 2013] Chiu, C., Lur, G., Morse, T., Carnevale, N., Ellis-Davies, G., and Higley, M. (2013). Compartmentalization of gabaergic inhibition by dendritic spines. *Science (New York, N.Y.)*, 340(6133):759–62.

[Clopath et al., 2010] Clopath, C., Busing, L., Vasilaki, E., and Gerstner, W. (2010). Connectivity reflects coding: a model of voltage-based stdp with homeostasis. *Nature neuroscience*, 13(3):344–52.

[Conde-Sousa and Aguiar, 2013] Conde-Sousa, E. and Aguiar, P. (2013). A working memory model for serial order that stores information in the intrinsic excitability properties of neurons. *Journal of computational neuroscience*, 35(2):187–99.

[Courtemanche et al., 1998] Courtemanche, M., Ramirez, R., and Nattel, S. (1998). Ionic mechanisms underlying human atrial action potential properties: insights from a mathematical model. *The American journal of physiology*, 275(1 Pt 2):H301–21.

[Crane et al., 2001] Crane, G., Hines, M., and Neild, T. (2001). Simulating the spread of membrane potential changes in arteriolar networks. *Microcirculation (New York, N.Y. : 1994)*, 8(1):33–43.

[Cruz et al., 2007] Cruz, G., Sahley, C., and Muller, K. (2007). Neuronal competition for action potential initiation sites in a circuit controlling simple learning. *Neuroscience*, 148(1):65–81.

[Culmone and Migliore, 2012] Culmone, V. and Migliore, M. (2012). Progressive effect of beta amyloid peptides accumulation on ca1 pyramidal neurons: a model study suggesting possible treatments. *Frontiers in computational neuroscience*, 6:52.

[Cutsuridis et al., 2010] Cutsuridis, V., Cobb, S., and Graham, B. (2010). Encoding and retrieval in a model of the hippocampal ca1 microcircuit. *Hippocampus*, 20(3):423–46.

[D'Angelo et al., 2001] D'Angelo, E., Nieus, T., Maffei, A., Armano, S., Rossi, P., Taglietti, V., Fontana, A., and Naldi, G. (2001). Theta-frequency bursting and resonance in cerebellar granule cells: experimental evidence and modeling of a slow k+-dependent mechanism. *The Journal of neuroscience : the official journal of the Society for Neuroscience*, 21(3):759–70.

[David et al., 2008] David, F., Linster, C., and Cleland, T. (2008). Lateral dendritic shunt inhibition can regularize mitral cell spike patterning. *Journal of computational neuroscience*, 25(1):25–38.

[Davison et al., 2000] Davison, A., Feng, J., and Brown, D. (2000). A reduced compartmental model of the mitral cell for use in network models of the olfactory bulb. *Brain research bulletin*, 51(5):393–9.

[Davison et al., 2003] Davison, A., Feng, J., and Brown, D. (2003). Dendrodendritic inhibition and simulated odor responses in a detailed olfactory bulb network model. *Journal of neurophysiology*, 90(3):1921–35.

[Davison and Fregnac, 2006] Davison, A. and Fregnac, Y. (2006). Learning cross-modal spatial transformations through spike timing-dependent plasticity. *The Journal of neuroscience : the official journal of the Society for Neuroscience*, 26(21):5604–15.

[Deister et al., 2009] Deister, C., Chan, C., Surmeier, D., and Wilson, C. (2009). Calcium-activated sk channels influence voltage-gated ion channels to determine the precision of firing in globus pallidus neurons. *The Journal of neuroscience : the official journal of the Society for Neuroscience*, 29(26):8452–61.

[Delgado et al., 2010] Delgado, J., Gomez-Gonzalez, J., and Desai, N. (2010). Pyramidal neuron conductance state gates spike-timing-dependent plasticity. *The Journal of neuroscience : the official journal of the Society for Neuroscience*, 30(47):15713–25.

[Destexhe et al., 1996a] Destexhe, A., Bal, T., McCormick, D., and Sejnowski, T. (1996a). Ionic mechanisms underlying synchronized oscillations and propagating waves in a model of ferret thalamic slices. *Journal of neurophysiology*, 76(3):2049–70.

[Destexhe et al., 1994a] Destexhe, A., Contreras, D., Sejnowski, T., and Steriade, M. (1994a). A model of spindle rhythmicity in the isolated thalamic reticular nucleus. *Journal of neurophysiology*, 72(2):803–18.

[Destexhe et al., 1998a] Destexhe, A., Contreras, D., and Steriade, M. (1998a). Mechanisms underlying the synchronizing action of corticothalamic feedback through inhibition of thalamic relay cells. *Journal of neurophysiology*, 79(2):999–1016.

[Destexhe et al., 1996b] Destexhe, A., Contreras, D., Steriade, M., Sejnowski, T., and Huguenard, J. (1996b). In vivo, in vitro, and computational analysis of dendritic calcium currents in thalamic reticular neurons. *The Journal of neuroscience : the official journal of the Society for Neuroscience*, 16(1):169–85.

[Destexhe et al., 1994b] Destexhe, A., Mainen, Z., and Sejnowski, T. (1994b). An efficient method for computing synaptic conductances based on a kinetic model of receptor binding. *Neural Computation*, 6:14–18.

[Destexhe et al., 1994c] Destexhe, A., Mainen, Z., and Sejnowski, T. (1994c). Synthesis of models for excitable membranes, synaptic transmission and neuromodulation using a common kinetic formalism. *Journal of computational neuroscience*, 1(3):195–230.

[Destexhe et al., 1998b] Destexhe, A., Neubig, M., Ulrich, D., and Huguenard, J. (1998b). Dendritic low-threshold calcium currents in thalamic relay cells. *The Journal of neuroscience : the official journal of the Society for Neuroscience*, 18(10):3574–88.

[Destexhe et al., 2001] Destexhe, A., Rudolph, M., Fellous, J., and Sejnowski, T. (2001). Fluctuating synaptic conductances recreate in vivo-like activity in neocortical neurons. *Neuroscience*, 107(1):13–24.

[Diba et al., 2006] Diba, K., Koch, C., and Segev, I. (2006). Spike propagation in dendrites with stochastic ion channels. *Journal of computational neuroscience*, 20(1):77–84.

[DiFrancesco and Noble, 1985] DiFrancesco, D. and Noble, D. (1985). A model of cardiac electrical activity incorporating ionic pumps and concentration changes. *Philosophical transactions of the Royal Society of London. Series B, Biological sciences*, 307(1133):353–98.

[Diwakar et al., 2011] Diwakar, S., Lombardo, P., Solinas, S., Naldi, G., and D'Angelo, E. (2011). Local field potential modeling predicts dense activation in cerebellar granule cells clusters under ltp and ltd control. *PloS one*, 6(7):e21928.

[Diwakar et al., 2009] Diwakar, S., Magistretti, J., Goldfarb, M., Naldi, G., and D'Angelo, E. (2009). Axonal na+ channels ensure fast spike activation and back-propagation in cerebellar granule cells. *Journal of neurophysiology*, 101(2):519–32.

[Djurisic et al., 2008] Djurisic, M., Popovic, M., Carnevale, N., and Zecevic, D. (2008). Functional structure of the mitral cell dendritic tuft in the rat olfactory bulb. *The Journal of neuroscience : the official journal of the Society for Neuroscience*, 28(15):4057–68.

[Dodge and Cooley, 1973] Dodge, F. and Cooley, J. (1973). Action potential of the motorneuron. *IBM J Res Dev*, 17:219–29.

[Durstewitz and Gabriel, 2007] Durstewitz, D. and Gabriel, T. (2007). Dynamical basis of irregular spiking in nmda-driven prefrontal cortex neurons. *Cerebral cortex (New York, N.Y. : 1991)*, 17(4):894–908.

[Durstewitz et al., 2000] Durstewitz, D., Seamans, J., and Sejnowski, T. (2000). Dopamine-mediated stabilization of delay-period activity in a network model of prefrontal cortex. *Journal of neurophysiology*, 83(3):1733–50.

[Dyhrfjeld-Johnsen et al., 2007] Dyhrfjeld-Johnsen, J., Santhakumar, V., Morgan, R., Huerta, R., Tsimring, L., and Soltesz, I. (2007). Topological determinants of epileptogenesis in large-scale structural and functional models of the dentate gyrus derived from experimental data. *Journal of neurophysiology*, 97(2):1566–87.

[Edin et al., 2007] Edin, F., Macoveanu, J., Olesen, P., Tegner, J., and Klingberg, T. (2007). Stronger synaptic connectivity as a mechanism behind development of working memory-related brain activity during childhood. *Journal of cognitive neuroscience*, 19(5):750–60.

[Ferrante et al., 2008] Ferrante, M., Blackwell, K., Migliore, M., and Ascoli, G. (2008). Computational models of neuronal biophysics and the characterization of potential neuropharmacological targets. *Current medicinal chemistry*, 15(24):2456–71.

[Ferrante et al., 2009] Ferrante, M., Migliore, M., and Ascoli, G. (2009). Feed-forward inhibition as a buffer of the neuronal input-output relation. *Proceedings of the National Academy of Sciences of the United States of America*, 106(42):18004–9.

[Ferrante et al., 2013] Ferrante, M., Migliore, M., and Ascoli, G. (2013). Functional impact of dendritic branch-point morphology. *The Journal of neuroscience : the official journal of the Society for Neuroscience*, 33(5):2156–65.

[Fietkiewicz et al., 2011] Fietkiewicz, C., Loparo, K., and Wilson, C. (2011). Drive latencies in hypoglossal motoneurons indicate developmental change in the brainstem respiratory network. *Journal of neural engineering*, 8(6):065011.

[Fineberg et al., 2012] Fineberg, J., Ritter, D., and Covarrubias, M. (2012). Modeling-independent elucidation of inactivation pathways in recombinant and native a-type kv channels. *The Journal of general physiology*, 140(5):513–27.

[Fink et al., 2000] Fink, C., Slepchenko, B., Moraru, I., Watras, J., Schaff, J., and Loew, L. (2000). An image-based model of calcium waves in differentiated neuroblastoma cells. *Biophysical journal*, 79(1):163–83.

[Fleidervish et al., 2010] Fleidervish, I., Lasser-Ross, N., Gutnick, M., and Ross, W. (2010). Na+ imaging reveals little difference in action potential-evoked na+ influx between axon and soma. *Nature neuroscience*, 13(7):852–60.

[Fohlmeister and Miller, 1997] Fohlmeister, J. and Miller, R. (1997). Impulse encoding mechanisms of ganglion cells in the tiger salamander retina. *Journal of neurophysiology*, 78(4):1935–47.

[Foust et al., 2011] Foust, A., Yu, Y., Popovic, M., Zecevic, D., and McCormick, D. (2011). Somatic membrane potential and kv1 channels control spike repolarization in cortical axon collaterals and presynaptic boutons. *The Journal of neuroscience : the official journal of the Society for Neuroscience*, 31(43):15490–8.

[Foutz et al., 2012] Foutz, T., Arlow, R., and McIntyre, C. (2012). Theoretical principles underlying optical stimulation of a channelrhodopsin-2 positive pyramidal neuron. *Journal of neurophysiology*, 107(12):3235–45.

[FRANKENHAEUSER and HUXLEY, 1964] FRANKENHAEUSER, B. and HUXLEY, A. (1964). The action potential in the myelinated nerve fiber of xenopus laevis as computed on the basis of voltage clamp data. *The Journal of physiology*, 171:302–15.

[Fujita et al., 2012] Fujita, T., Fukai, T., and Kitano, K. (2012). Influences of membrane properties on phase response curve and synchronization stability in a model globus pallidus neuron. *Journal of computational neuroscience*, 32(3):539–53.

[Gabbiani et al., 1994] Gabbiani, F., Midtgaard, J., and Knopfel, T. (1994). Synaptic integration in a model of cerebellar granule cells. *Journal of neurophysiology*, 72(2):999–1009.

[Gasparini et al., 2004] Gasparini, S., Migliore, M., and Magee, J. (2004). On the initiation and propagation of dendritic spikes in ca1 pyramidal neurons. *The Journal of neuroscience : the official journal of the Society for Neuroscience*, 24(49):11046–56.

[Geiger et al., 1997] Geiger, J., Lubke, J., Roth, A., Frotscher, M., and Jonas, P. (1997). Submillisecond ampa receptor-mediated signaling at a principal neuron-interneuron synapse. *Neuron*, 18(6):1009–23.

[Getting, 1983] Getting, P. (1983). Mechanisms of pattern generation underlying swimming in tritonia. ii. network reconstruction. *Journal of neurophysiology*, 49(4):1017–35.

[Gillies and Willshaw, 2006] Gillies, A. and Willshaw, D. (2006). Membrane channel interactions underlying rat subthalamic projection neuron rhythmic and bursting activity. *Journal of neurophysiology*, 95(4):2352–65.

[Gold et al., 2007] Gold, C., Henze, D., and Koch, C. (2007). Using extracellular action potential recordings to constrain compartmental models. *Journal of computational neuroscience*, 23(1):39–58.

[Gold et al., 2006] Gold, C., Henze, D., Koch, C., and Buzsaki, G. (2006). On the origin of the extracellular action potential waveform: A modeling study. *Journal of neurophysiology*, 95(5):3113–28.

[Golding et al., 2001] Golding, N., Kath, W., and Spruston, N. (2001). Dichotomy of action-potential backpropagation in ca1 pyramidal neuron dendrites. *Journal of neurophysiology*, 86(6):2998–3010.

[Golding et al., 2005] Golding, N., Mickus, T., Katz, Y., Kath, W., and Spruston, N. (2005). Factors mediating powerful voltage attenuation along ca1 pyramidal neuron dendrites. *The Journal of physiology*, 568(Pt 1):69–82.

[Golomb et al., 2006] Golomb, D., Yue, C., and Yaari, Y. (2006). Contribution of persistent na+ current and m-type k+ current to somatic bursting in ca1 pyramidal cells: combined experimental and modeling study. *Journal of neurophysiology*, 96(4):1912–26.

[Gomez Gonzalez et al., 2011] Gomez Gonzalez, J., Mel, B., and Poirazi, P. (2011). Distinguishing linear vs. non-linear integration in ca1 radial oblique dendrites: It's about time. *Frontiers in computational neuroscience*, 5:44.

[Gouwens and Wilson, 2009] Gouwens, N. and Wilson, R. (2009). Signal propagation in drosophila central neurons. *The Journal of neuroscience : the official journal of the Society for Neuroscience*, 29(19):6239–49.

[Graham, 2001] Graham, B. (2001). Pattern recognition in a compartmental model of a ca1 pyramidal neuron. *Network (Bristol, England)*, 12(4):473–92.

[Graham et al., 2014] Graham, B., Saudargiene, A., and Cobb, S. (2014). Spine head calcium as a measure of summed postsynaptic activity for driving synaptic plasticity. *Neural computation*, 26(10):2194–222.

[Graham et al., 2001] Graham, B., Wong, A., and Forsythe, I. (2001). A computational model of synaptic transmission at the calyx of held. *Neurocomputing*, 38:37–42.

[Gruber et al., 2003] Gruber, A., Solla, S., Surmeier, D., and Houk, J. (2003). Modulation of striatal single units by expected reward: a spiny neuron model displaying dopamine-induced bistability. *Journal of neurophysiology*, 90(2):1095–114.

[Grunditz et al., 2008] Grunditz, A., Holbro, N., Tian, L., Zuo, Y., and Oertner, T. (2008). Spine neck plasticity controls postsynaptic calcium signals through electrical compartmentalization. *The Journal of neuroscience : the official journal of the Society for Neuroscience*, 28(50):13457–66.

[Gurkiewicz and Korngreen, 2007] Gurkiewicz, M. and Korngreen, A. (2007). A numerical approach to ion channel modelling using whole-cell voltage-clamp recordings and a genetic algorithm. *PLoS computational biology*, 3(8):e169.

[Gurkiewicz et al., 2011] Gurkiewicz, M., Korngreen, A., Waxman, S., and Lampert, A. (2011). Kinetic modeling of nav1.7 provides insight into erythromelalgia-associated f1449v mutation. *Journal of neurophysiology*, 105(4):1546–57.

[Haag et al., 1997] Haag, J., Theunissen, F., and Borst, A. (1997). The intrinsic electrophysiological characteristics of fly lobula plate tangential cells: Ii. active membrane properties. *Journal of computational neuroscience*, 4(4):349–69.

[Haag et al., 1999] Haag, J., Vermeulen, A., and Borst, A. (1999). The intrinsic electrophysiological characteristics of fly lobula plate tangential cells: Iii. visual response properties. *Journal of computational neuroscience*, 7(3):213–34.

[Hahn and McIntyre, 2010] Hahn, P. and McIntyre, C. (2010). Modeling shifts in the rate and pattern of subthalamopallidal network activity during deep brain stimulation. *Journal of computational neuroscience*, 28(3):425–41.

[Hallermann et al., 2012] Hallermann, S., de Kock, C., Stuart, G., and Kole, M. (2012). State and location dependence of action potential metabolic cost in cortical pyramidal neurons. *Nature neuroscience*, 15(7):1007–14.

[Halnes et al., 2011] Halnes, G., Augustinaite, S., Heggelund, P., Einevoll, G., and Migliore, M. (2011). A multi-compartment model for interneurons in the dorsal lateral geniculate nucleus. *PLoS computational biology*, 7(9):e1002160.

[Hao et al., 2009] Hao, J., Wang, X., Dan, Y., Poo, M., and Zhang, X. (2009). An arithmetic rule for spatial summation of excitatory and inhibitory inputs in pyramidal neurons. *Proceedings of the National Academy of Sciences of the United States of America*, 106(51):21906–11.

[Hay et al., 2011] Hay, E., Hill, S., Schurmann, F., Markram, H., and Segev, I. (2011). Models of neocortical layer 5b pyramidal cells capturing a wide range of dendritic and perisomatic active properties. *PLoS computational biology*, 7(7):e1002107.

[Hay et al., 2013] Hay, E., Schurmann, F., Markram, H., and Segev, I. (2013). Preserving axosomatic spiking features despite diverse dendritic morphology. *Journal of neurophysiology*, 109(12):2972–81.

[Helmstaedter et al., 2009a] Helmstaedter, M., Sakmann, B., and Feldmeyer, D. (2009a). L2/3 interneuron groups defined by multiparameter analysis of axonal projection, dendritic geometry, and electrical excitability. *Cerebral cortex (New York, N.Y. : 1991)*, 19(4):951–62.

[Helmstaedter et al., 2009b] Helmstaedter, M., Sakmann, B., and Feldmeyer, D. (2009b). Neuronal correlates of local, lateral, and translaminar inhibition with reference to cortical columns. *Cerebral cortex (New York, N.Y. : 1991)*, 19(4):926–37.

[Helmstaedter et al., 2009c] Helmstaedter, M., Sakmann, B., and Feldmeyer, D. (2009c). The relation between dendritic geometry, electrical excitability, and axonal projections of l2/3 interneurons in rat barrel cortex. *Cerebral cortex (New York, N.Y. : 1991)*, 19(4):938–50.

[Helmstaedter et al., 2008] Helmstaedter, M., Staiger, J., Sakmann, B., and Feldmeyer, D. (2008). Efficient recruitment of layer 2/3 interneurons by layer 4 input in single columns of rat somatosensory cortex. *The Journal of neuroscience : the official journal of the Society for Neuroscience*, 28(33):8273–84.

[Hemond et al., 2008] Hemond, P., Epstein, D., Boley, A., Migliore, M., Ascoli, G., and Jaffe, D. (2008). Distinct classes of pyramidal cells exhibit mutually exclusive firing patterns in hippocampal area ca3b. *Hippocampus*, 18(4):411–24.

[Hemond et al., 2009] Hemond, P., Migliore, M., Ascoli, G., and Jaffe, D. (2009). The membrane response of hippocampal ca3b pyramidal neurons near rest: Heterogeneity of passive properties and the contribution of hyperpolarization-activated currents. *Neuroscience*, 160(2):359–70.

[Herzog et al., 2001] Herzog, R., Cummins, T., and Waxman, S. (2001). Persistent ttx-resistant na+ current affects resting potential and response to depolarization in simulated spinal sensory neurons. *Journal of neurophysiology*, 86(3):1351–64.

[Hines and Carnevale, 2001] Hines, M. and Carnevale, N. (2001). Neuron: a tool for neuroscientists. *The Neuroscientist : a review journal bringing neurobiology, neurology and psychiatry*, 7(2):123–35.

[Hines and Carnevale, 2008] Hines, M. and Carnevale, N. (2008). Translating network models to parallel hardware in neuron. *Journal of neuroscience methods*, 169(2):425–55.

[Hines et al., 2008] Hines, M., Eichner, H., and Schurmann, F. (2008). Neuron splitting in compute-bound parallel network simulations enables runtime scaling with twice as many processors. *Journal of computational neuroscience*, 25(1):203–10.

[Hines et al., 2011] Hines, M., Kumar, S., and Schurmann, F. (2011). Comparison of neuronal spike exchange methods on a blue gene/p supercomputer. *Frontiers in computational neuroscience*, 5:49.

[Hines and Shrager, 1991] Hines, M. and Shrager, P. (1991). A computational test of the requirements for conduction in demyelinated axons. *Restorative neurology and neuroscience*, 3(2):81–93.

[Holt and Koch, 1999] Holt, G. and Koch, C. (1999). Electrical interactions via the extracellular potential near cell bodies. *Journal of computational neuroscience*, 6(2):169–84.

[Hopfield and Brody, 2000] Hopfield, J. and Brody, C. (2000). What is a moment? "cortical" sensory integration over a brief interval. *Proceedings of the National Academy of Sciences of the United States of America*, 97(25):13919–24.

[Hopfield and Brody, 2001] Hopfield, J. and Brody, C. (2001). What is a moment? transient synchrony as a collective mechanism for spatiotemporal integration. *Proceedings of the National Academy of Sciences of the United States of America*, 98(3):1282–7.

[Hossain et al., 2005] Hossain, W., Antic, S., Yang, Y., Rasband, M., and Morest, D. (2005). Where is the spike generator of the cochlear nerve? voltage-gated sodium channels in the mouse cochlea. *The Journal of neuroscience : the official journal of the Society for Neuroscience*, 25(29):6857–68.

[Hu et al., 2009] Hu, W., Tian, C., Li, T., Yang, M., Hou, H., and Shu, Y. (2009). Distinct contributions of na(v)1.6 and na(v)1.2 in action potential initiation and backpropagation. *Nature neuroscience*, 12(8):996–1002.

[Huang and Robinson, 1998] Huang, S. and Robinson, D. (1998). Activation and inactivation properties of voltage-gated calcium currents in developing cat retinal ganglion cells. *Neuroscience*, 85(1):239–47.

[Huguenard et al., 1997] Huguenard, J., McCormick, D., and Shepherd, G. (1997). Electrophysiology of the neuron, a companion to shepherd's neurobiology, an interactive tutorial. *Electrophysiology of the Neuron*.

[Izhikevich, 2003] Izhikevich, E. (2003). Simple model of spiking neurons. *IEEE transactions on neural networks / a publication of the IEEE Neural Networks Council*, 14(6):1569–72.

[Jaffe et al., 2011] Jaffe, D., Wang, B., and Brenner, R. (2011). Shaping of action potentials by type i and type ii large-conductance ca(2)+-activated k+ channels. *Neuroscience*, 192:205–18.

[Jarsky et al., 2005] Jarsky, T., Roxin, A., Kath, W., and Spruston, N. (2005). Conditional dendritic spike propagation following distal synaptic activation of hippocampal ca1 pyramidal neurons. *Nature neuroscience*, 8(12):1667–76.

[Jedlicka et al., 2011] Jedlicka, P., Deller, T., Gutkin, B., and Backus, K. (2011). Activity-dependent intracellular chloride accumulation and diffusion controls gaba(a) receptor-mediated synaptic transmission. *Hippocampus*, 21(8):885–98.

[Johnson and McIntyre, 2008] Johnson, M. and McIntyre, C. (2008). Quantifying the neural elements activated and inhibited by globus pallidus deep brain stimulation. *Journal of neurophysiology*, 100(5):2549–63.

[Jones and Gabbiani, 2012a] Jones, P. and Gabbiani, F. (2012a). Impact of neural noise on a sensory-motor pathway signaling impending collision. *Journal of neurophysiology*, 107(4):1067–79.

[Jones and Gabbiani, 2012b] Jones, P. and Gabbiani, F. (2012b). Logarithmic compression of sensory signals within the dendritic tree of a collision-sensitive neuron. *The Journal of neuroscience : the official journal of the Society for Neuroscience*, 32(14):4923–34.

[Jones et al., 2009] Jones, S., Pritchett, D., Sikora, M., Stufflebeam, S., Hamalainen, M., and Moore, C. (2009). Quantitative analysis and biophysically realistic neural modeling of the meg mu rhythm: rhythmogenesis and modulation of sensory-evoked responses. *Journal of neurophysiology*, 102(6):3554–72.

[Jones et al., 2007] Jones, S., Pritchett, D., Stufflebeam, S., Hamalainen, M., and Moore, C. (2007). Neural correlates of tactile detection: a combined magnetoencephalography and biophysically based computational modeling study. *The Journal of neuroscience : the official journal of the Society for Neuroscience*, 27(40):10751–64.

[Kampa et al., 2004] Kampa, B., Clements, J., Jonas, P., and Stuart, G. (2004). Kinetics of mg2+ unblock of nmda receptors: implications for spike-timing dependent synaptic plasticity. *The Journal of physiology*, 556(Pt 2):337–45.

[Kampa and Stuart, 2006] Kampa, B. and Stuart, G. (2006). Calcium spikes in basal dendrites of layer 5 pyramidal neurons during action potential bursts. *The Journal of neuroscience : the official journal of the Society for Neuroscience*, 26(28):7424–32.

[Kanold and Manis, 1999] Kanold, P. and Manis, P. (1999). Transient potassium currents regulate the discharge patterns of dorsal cochlear nucleus pyramidal cells. *The Journal of neuroscience : the official journal of the Society for Neuroscience*, 19(6):2195–208.

[Kanold and Manis, 2001] Kanold, P. and Manis, P. (2001). A physiologically based model of discharge pattern regulation by transient k+ currents in cochlear nucleus pyramidal cells. *Journal of neurophysiology*, 85(2):523–38.

[Kaplan and Lansner, 2014] Kaplan, B. and Lansner, A. (2014). A spiking neural network model of self-organized pattern recognition in the early mammalian olfactory system. *Frontiers in neural circuits*, 8:5.

[Katona et al., 2011] Katona, G., Kaszas, A., Turi, G., Hajos, N., Tamas, G., Vizi, E., and Rozsa, B. (2011). Roller coaster scanning reveals spontaneous triggering of dendritic spikes in ca1 interneurons. *Proceedings of the National Academy of Sciences of the United States of America*, 108(5):2148–53.

[Katz et al., 2009] Katz, Y., Menon, V., Nicholson, D., Geinisman, Y., Kath, W., and Spruston, N. (2009). Synapse distribution suggests a two-stage model of dendritic integration in ca1 pyramidal neurons. *Neuron*, 63(2):171–7.

[Kerr et al., 2012] Kerr, C., Neymotin, S., Chadderdon, G., Fietkiewicz, C., Francis, J., and Lytton, W. (2012). Electrostimulation as a prosthesis for repair of information flow in a computer model of neocortex. *IEEE transactions on neural systems and rehabilitation engineering : a publication of the IEEE Engineering in Medicine and Biology Society*, 20(2):153–60.

[Khaliq et al., 2003] Khaliq, Z., Gouwens, N., and Raman, I. (2003). The contribution of resurgent sodium current to high-frequency firing in purkinje neurons: an experimental and modeling study. *The Journal of neuroscience : the official journal of the Society for Neuroscience*, 23(12):4899–912.

[Kim et al., 2013] Kim, D., Pare, D., and Nair, S. (2013). Mechanisms contributing to the induction and storage of pavlovian fear memories in the lateral amygdala. *Learning & memory (Cold Spring Harbor, N.Y.)*, 20(8):421–30.

[Kole et al., 2006] Kole, M., Hallermann, S., and Stuart, G. (2006). Single ih channels in pyramidal neuron dendrites: properties, distribution, and impact on action potential output. *The Journal of neuroscience : the official journal of the Society for Neuroscience*, 26(6):1677–87.

[Kole et al., 2008] Kole, M., Ilschner, S., Kampa, B., Williams, S., Ruben, P., and Stuart, G. (2008). Action potential generation requires a high sodium channel density in the axon initial segment. *Nature neuroscience*, 11(2):178–86.

[Komendantov et al., 2004] Komendantov, A., Komendantova, O., Johnson, S., and Canavier, C. (2004). A modeling study suggests complementary roles for gabaa and nmda receptors and the sk channel in regulating the firing pattern in midbrain dopamine neurons. *Journal of neurophysiology*, 91(1):346–57.

[Kondgen et al., 2008] Kondgen, H., Geisler, C., Fusi, S., Wang, X., Luscher, H., and Giugliano, M. (2008). The dynamical response properties of neocortical neurons to temporally modulated noisy inputs in vitro. *Cerebral cortex (New York, N.Y. : 1991)*, 18(9):2086–97.

[Kopp-Scheinpflug et al., 2011] Kopp-Scheinpflug, C., Tozer, A., Robinson, S., Tempel, B., Hennig, M., and Forsythe, I. (2011). The sound of silence: ionic mechanisms encoding sound termination. *Neuron*, 71(5):911–25.

[Korngreen et al., 2005] Korngreen, A., Kaiser, K., and Zilberter, Y. (2005). Subthreshold inactivation of voltage-gated k+ channels modulates action potentials in neocortical bitufted interneurones from rats. *The Journal of physiology*, 562(Pt 2):421–37.

[Korngreen and Sakmann, 2000] Korngreen, A. and Sakmann, B. (2000). Voltage-gated k+ channels in layer 5 neocortical pyramidal neurones from young rats: subtypes and gradients. *The Journal of physiology*, 525 Pt 3:621–39.

[Korogod and Kulagina, 1998] Korogod, S. and Kulagina, I. (1998). Geometry-induced features of current transfer in neuronal dendrites with tonically activated conductances. *Biological cybernetics*, 79(3):231–40.

[Korogod et al., 1999] Korogod, S., Kulagina, I., and Tyc-Dumont, S. (1999). Transfer properties of neuronal dendrites with tonically activated conductances. *Neurophysiology*, 30:203–207.

[Kourennyi et al., 2004] Kourennyi, D., Liu, X., Hart, J., Mahmud, F., Baldridge, W., and Barnes, S. (2004). Reciprocal modulation of calcium dynamics at rod and cone photoreceptor synapses by nitric oxide. *Journal of neurophysiology*, 92(1):477–83.

[Kovalsky et al., 2009] Kovalsky, Y., Amir, R., and Devor, M. (2009). Simulation in sensory neurons reveals a key role for delayed na+ current in subthreshold oscillations and ectopic discharge: implications for neuropathic pain. *Journal of neurophysiology*, 102(3):1430–42.

[Kuznetsova et al., 2010] Kuznetsova, A., Huertas, M., Kuznetsov, A., Paladini, C., and Canavier, C. (2010). Regulation of firing frequency in a computational model of a midbrain dopaminergic neuron. *Journal of computational neuroscience*, 28(3):389–403.

[Larkum et al., 2009] Larkum, M., Nevian, T., Sandler, M., Polsky, A., and Schiller, J. (2009). Synaptic integration in tuft dendrites of layer 5 pyramidal neurons: a new unifying principle. *Science (New York, N.Y.)*, 325(5941):756–60.

[Lavzin et al., 2012] Lavzin, M., Rapoport, S., Polsky, A., Garion, L., and Schiller, J. (2012). Nonlinear dendritic processing determines angular tuning of barrel cortex neurons in vivo. *Nature*, 490(7420):397–401.

[Lawrence et al., 2006] Lawrence, J., Saraga, F., Churchill, J., Statland, J., Travis, K., Skinner, F., and McBain, C. (2006). Somatodendritic kv7/kcnq/m channels control interspike interval in hippocampal interneurons. *The Journal of neuroscience : the official journal of the Society for Neuroscience*, 26(47):12325–38.

[Lazarewicz et al., 2002] Lazarewicz, M., Migliore, M., and Ascoli, G. (2002). A new bursting model of ca3 pyramidal cell physiology suggests multiple locations for spike initiation. *Bio Systems*, 67(1-3):129–37.

[Lee, 2007] Lee, J. (2007). Fast rhythmic bursting cells: the horizontal fiber system in the cat's primary visual cortex. *Penn McNair Research Journal*, 1(1):1.

[Lee et al., 2003] Lee, S., Hayashida, Y., and Ishida, A. (2003). Availability of low-threshold ca2+ current in retinal ganglion cells. *Journal of neurophysiology*, 90(6):3888–901.

[Lee et al., 2014] Lee, S., Marchionni, I., Bezaire, M., Varga, C., Danielson, N., Lovett-Barron, M., Losonczy, A., and Soltesz, I. (2014). Parvalbumin-positive basket cells differentiate among hippocampal pyramidal cells. *Neuron*, 82(5):1129–44.

[Letzkus et al., 2006] Letzkus, J., Kampa, B., and Stuart, G. (2006). Learning rules for spike timing-dependent plasticity depend on dendritic synapse location. *The Journal of neuroscience : the official journal of the Society for Neuroscience*, 26(41):10420–9.

[Li and Cleland, 2013] Li, G. and Cleland, T. (2013). A two-layer biophysical model of cholinergic neuromodulation in olfactory bulb. *The Journal of neuroscience : the official journal of the Society for Neuroscience*, 33(7):3037–58.

[Li and Ascoli, 2006] Li, X. and Ascoli, G. (2006). Computational simulation of the input-output relationship in hippocampal pyramidal cells. *Journal of computational neuroscience*, 21(2):191–209.

[Li and Ascoli, 2008] Li, X. and Ascoli, G. (2008). Effects of synaptic synchrony on the neuronal input-output relationship. *Neural computation*, 20(7):1717–31.

[Li et al., 2013] Li, X., Morita, K., Robinson, H., and Small, M. (2013). Control of layer 5 pyramidal cell spiking by oscillatory inhibition in the distal apical dendrites: a computational modeling study. *Journal of neurophysiology*, 109(11):2739–56.

[Lien et al., 2002] Lien, C., Martina, M., Schultz, J., Ehmke, H., and Jonas, P. (2002). Gating, modulation and subunit composition of voltage-gated k(+) channels in dendritic inhibitory interneurones of rat hippocampus. *The Journal of physiology*, 538(Pt 2):405–19.

[Linaro et al., 2011] Linaro, D., Storace, M., and Giugliano, M. (2011). Accurate and fast simulation of channel noise in conductance-based model neurons by diffusion approximation. *PLoS computational biology*, 7(3):e1001102.

[Liu and Kourennyi, 2004] Liu, X. and Kourennyi, D. (2004). Effects of tetraethylammonium on kx channels and simulated light response in rod photoreceptors. *Annals of biomedical engineering*, 32(10):1428–42.

[Liu et al., 1998] Liu, Z., Golowasch, J., Marder, E., and Abbott, L. (1998). A model neuron with activity-dependent conductances regulated by multiple calcium sensors. *The Journal of neuroscience : the official journal of the Society for Neuroscience*, 18(7):2309–20.

[Luthman et al., 2011] Luthman, J., Hoebeek, F., Maex, R., Davey, N., Adams, R., De Zeeuw, C., and Steuber, V. (2011). Std-dependent and independent encoding of input irregularity as spike rate in a computational model of a cerebellar nucleus neuron. *Cerebellum (London, England)*, 10(4):667–82.

[Lytton, 1997] Lytton, W. (1997). Computer model of clonazepam's effect in thalamic slice. *Neuroreport*, 8(15):3339–43.

[Lytton, 1998] Lytton, W. (1998). Adapting a feedforward heteroassociative network to hodgkin-huxley dynamics. *Journal of computational neuroscience*, 5(4):353–64.

[Lytton, 2006] Lytton, W. (2006). Neural query system: Data-mining from within the neuron simulator. *Neuroinformatics*, 4(2):163–76.

[Lytton et al., 1997] Lytton, W., Contreras, D., Destexhe, A., and Steriade, M. (1997). Dynamic interactions determine partial thalamic quiescence in a computer network model of spike-and-wave seizures. *Journal of neurophysiology*, 77(4):1679–96.

[Lytton and Hines, 2005] Lytton, W. and Hines, M. (2005). Independent variable time-step integration of individual neurons for network simulations. *Neural computation*, 17(4):903–21.

[Lytton and Lipton, 1999] Lytton, W. and Lipton, P. (1999). Can the hippocampus tell time? the temporo-septal engram shift model. *Neuroreport*, 10(11):2301–6.

[Lytton et al., 2008a] Lytton, W., Neymotin, S., and Hines, M. (2008a). The virtual slice setup. *Journal of neuroscience methods*, 171(2):309–15.

[Lytton and Omurtag, 2007] Lytton, W. and Omurtag, A. (2007). Tonic-clonic transitions in computer simulation. *Journal of clinical neurophysiology : official publication of the American Electroencephalographic Society*, 24(2):175–81.

[Lytton et al., 2008b] Lytton, W., Orman, R., and Stewart, M. (2008b). Broadening of activity with flow across neural structures. *Perception*, 37(3):401–7.

[Mahon et al., 2000] Mahon, S., Deniau, J., Charpier, S., and Delord, B. (2000). Role of a striatal slowly inactivating potassium current in short-term facilitation of corticostriatal inputs: a computer simulation study. *Learning & memory (Cold Spring Harbor, N.Y.)*, 7(5):357–62.

[Mai and Liu, 2009] Mai, Z. and Liu, H. (2009). Boolean network-based analysis of the apoptosis network: irreversible apoptosis and stable surviving. *Journal of theoretical biology*, 259(4):760–9.

[Mainen et al., 1995] Mainen, Z., Joerges, J., Huguenard, J., and Sejnowski, T. (1995). A model of spike initiation in neocortical pyramidal neurons. *Neuron*, 15(6):1427–39.

[Mainen and Sejnowski, 1996] Mainen, Z. and Sejnowski, T. (1996). Influence of dendritic structure on firing pattern in model neocortical neurons. *Nature*, 382(6589):363–6.

[Marasco et al., 2012] Marasco, A., Limongiello, A., and Migliore, M. (2012). Fast and accurate low-dimensional reduction of biophysically detailed neuron models. *Scientific reports*, 2:928.

[Marasco et al., 2013] Marasco, A., Limongiello, A., and Migliore, M. (2013). Using strahler's analysis to reduce up to 200-fold the run time of realistic neuron models. *Scientific reports*, 3:2934.

[Marcelin et al., 2009] Marcelin, B., Chauviere, L., Becker, A., Migliore, M., Esclapez, M., and Bernard, C. (2009). h channel-dependent deficit of theta oscillation resonance and phase shift in temporal lobe epilepsy. *Neurobiology of disease*, 33(3):436–47.

[Markaki et al., 2005] Markaki, M., Orphanoudakis, S., and Poirazi, P. (2005). Modelling reduced excitability in aged ca1 neurons as a calcium-dependent process. *Neurocomputing*, 65-66:305–314.

[Masurkar and Chen, 2011a] Masurkar, A. and Chen, W. (2011a). Calcium currents of olfactory bulb juxtaglomerular cells: profile and multiple conductance plateau potential simulation. *Neuroscience*, 192:231–46.

[Masurkar and Chen, 2011b] Masurkar, A. and Chen, W. (2011b). Potassium currents of olfactory bulb juxtaglomerular cells: characterization, simulation, and implications for plateau potential firing. *Neuroscience*, 192:247–62.

[Mattioni and Le Novere, 2013] Mattioni, M. and Le Novere, N. (2013). Integration of biochemical and electrical signaling-multiscale model of the medium spiny neuron of the striatum. *PloS one*, 8(7):e66811.

[Maurice et al., 2004] Maurice, N., Mercer, J., Chan, C., Hernandez-Lopez, S., Held, J., Tkatch, T., and Surmeier, D. (2004). D2 dopamine receptor-mediated modulation of voltage-dependent na+ channels reduces autonomous activity in striatal cholinergic interneurons. *The Journal of neuroscience : the official journal of the Society for Neuroscience*, 24(46):10289–301.

[McCormick and Pape, 1990] McCormick, D. and Pape, H. (1990). Properties of a hyperpolarization-activated cation current and its role in rhythmic oscillation in thalamic relay neurones. *The Journal of physiology*, 431:291–318.

[McCormick et al., 2007] McCormick, D., Shu, Y., and Yu, Y. (2007). Neurophysiology: Hodgkin and huxley model–still standing? *Nature*, 445(7123):E1–2; discussion E2–3.

[McIntyre et al., 2002] McIntyre, C., Richardson, A., and Grill, W. (2002). Modeling the excitability of mammalian nerve fibers: influence of afterpotentials on the recovery cycle. *Journal of neurophysiology*, 87(2):995–1006.

[McRory et al., 2001] McRory, J., Santi, C., Hamming, K., Mezeyova, J., Sutton, K., Baillie, D., Stea, A., and Snutch, T. (2001). Molecular and functional characterization of a family of rat brain t-type calcium channels. *The Journal of biological chemistry*, 276(6):3999–4011.

[McTavish et al., 2012] McTavish, T., Migliore, M., Shepherd, G., and Hines, M. (2012). Mitral cell spike synchrony modulated by dendrodendritic synapse location. *Frontiers in computational neuroscience*, 6:3.

[Melnick et al., 2004a] Melnick, I., Santos, S., and Safronov, B. (2004a). Mechanism of spike frequency adaptation in substantia gelatinosa neurones of rat. *The Journal of physiology*, 559(Pt 2):383–95.

[Melnick et al., 2004b] Melnick, I., Santos, S., Szokol, K., Szucs, P., and Safronov, B. (2004b). Ionic basis of tonic firing in spinal substantia gelatinosa neurons of rat. *Journal of neurophysiology*, 91(2):646–55.

[Mennerick et al., 1997] Mennerick, S., Zenisek, D., and Matthews, G. (1997). Static and dynamic membrane properties of large-terminal bipolar cells from goldfish retina: experimental test of a compartment model. *Journal of neurophysiology*, 78(1):51–62.

[Mercer et al., 2007] Mercer, J., Chan, C., Tkatch, T., Held, J., and Surmeier, D. (2007). Nav1.6 sodium channels are critical to pacemaking and fast spiking in globus pallidus neurons. *The Journal of neuroscience : the official journal of the Society for Neuroscience*, 27(49):13552–66.

[Meuth et al., 2005] Meuth, P., Meuth, S., Jacobi, D., Broicher, T., Pape, H., and Budde, T. (2005). Get the rhythm: modeling neuronal activity. *Journal of undergraduate neuroscience education : JUNE : a publication of FUN, Faculty for Undergraduate Neuroscience*, 4(1):A1–A11.

[Miceli et al., 2013] Miceli, F., Soldovieri, M., Ambrosino, P., Barrese, V., Migliore, M., Cilio, M., and Taglialatela, M. (2013). Genotype-phenotype correlations in neonatal epilepsies caused by mutations in the voltage sensor of k(v)7.2 potassium channel subunits. *Proceedings of the National Academy of Sciences of the United States of America*, 110(11):4386–91.

[Miceli et al., 2009] Miceli, F., Soldovieri, M., Lugli, L., Bellini, G., Ambrosino, P., Migliore, M., del Giudice, E., Ferrari, F., Pascotto, A., and Taglialatela, M. (2009). Neutralization of a unique, negatively-charged residue in the voltage sensor of k v 7.2 subunits in a sporadic case of benign familial neonatal seizures. *Neurobiology of disease*, 34(3):501–10.

[Migliore, 1996] Migliore, M. (1996). Modeling the attenuation and failure of action potentials in the dendrites of hippocampal neurons. *Biophysical journal*, 71(5):2394–403.

[Migliore, 2003] Migliore, M. (2003). On the integration of subthreshold inputs from perforant path and schaffer collaterals in hippocampal ca1 pyramidal neurons. *Journal of computational neuroscience*, 14(2):185–92.

[Migliore et al., 2008a] Migliore, M., Cannia, C., and Canavier, C. (2008a). A modeling study suggesting a possible pharmacological target to mitigate the effects of ethanol on reward-related dopaminergic signaling. *Journal of neurophysiology*, 99(5):2703–7.

[Migliore et al., 2006] Migliore, M., Cannia, C., Lytton, W., Markram, H., and Hines, M. (2006). Parallel network simulations with neuron. *Journal of computational neuroscience*, 21(2):119–29.

[Migliore et al., 2014] Migliore, M., Cavarretta, F., Hines, M., and Shepherd, G. (2014). Distributed organization of a brain microcircuit analyzed by three-dimensional modeling: the olfactory bulb. *Frontiers in computational neuroscience*, 8:50.

[Migliore et al., 1995] Migliore, M., Cook, E., Jaffe, D., Turner, D., and Johnston, D. (1995). Computer simulations of morphologically reconstructed ca3 hippocampal neurons. *Journal of neurophysiology*, 73(3):1157–68.

[Migliore et al., 2011] Migliore, M., De Blasi, I., Tegolo, D., and Migliore, R. (2011). A modeling study suggesting how a reduction in the context-dependent input on ca1 pyramidal neurons could generate schizophrenic behavior. *Neural networks : the official journal of the International Neural Network Society*, 24(6):552–9.

[Migliore et al., 2005a] Migliore, M., Ferrante, M., and Ascoli, G. (2005a). Signal propagation in oblique dendrites of ca1 pyramidal cells. *Journal of neurophysiology*, 94(6):4145–55.

[Migliore et al., 2010] Migliore, M., Hines, M., McTavish, T., and Shepherd, G. (2010). Functional roles of distributed synaptic clusters in the mitral-granule cell network of the olfactory bulb. *Frontiers in integrative neuroscience*, 4:122.

[Migliore et al., 2005b] Migliore, M., Hines, M., and Shepherd, G. (2005b). The role of distal dendritic gap junctions in synchronization of mitral cell axonal output. *Journal of computational neuroscience*, 18(2):151–61.

[Migliore et al., 1999] Migliore, M., Hoffman, D., Magee, J., and Johnston, D. (1999). Role of an a-type k+ conductance in the back-propagation of action potentials in the dendrites of hippocampal pyramidal neurons. *Journal of computational neuroscience*, 7(1):5–15.

[Migliore et al., 2007] Migliore, M., Inzirillo, C., and Shepherd, G. (2007). Learning mechanism for column formation in the olfactory bulb. *Frontiers in integrative neuroscience*, 1:12.

[Migliore and McTavish, 2013] Migliore, M. and McTavish, T. (2013). Olfactory computation in mitral-granule cell circuits. *Encyclopedia of Computational Neuroscience.*

[Migliore et al., 2001] Migliore, M., Messineo, L., Cardaci, M., and Ayala, G. (2001). Quantitative modeling of perception and production of time intervals. *Journal of neurophysiology*, 86(6):2754–60.

[Migliore et al., 2004] Migliore, M., Messineo, L., and Ferrante, M. (2004). Dendritic ih selectively blocks temporal summation of unsynchronized distal inputs in ca1 pyramidal neurons. *Journal of computational neuroscience*, 16(1):5–13.

[Migliore and Migliore, 2012] Migliore, M. and Migliore, R. (2012). Know your current i(h): interaction with a shunting current explains the puzzling effects of its pharmacological or pathological modulations. *PloS one*, 7(5):e36867.

[Migliore et al., 2008b] Migliore, M., Novara, G., and Tegolo, D. (2008b). Single neuron binding properties and the magical number 7. *Hippocampus*, 18(11):1122–30.

[Migliore and Shepherd, 2002] Migliore, M. and Shepherd, G. (2002). Emerging rules for the distributions of active dendritic conductances. *Nature reviews. Neuroscience*, 3(5):362–70.

[Migliore and Shepherd, 2008] Migliore, M. and Shepherd, G. (2008). Dendritic action potentials connect distributed dendrodendritic microcircuits. *Journal of computational neuroscience*, 24(2):207–21.

[Minneci et al., 2007] Minneci, F., Janahmadi, M., Migliore, M., Dragicevic, N., Avossa, D., and Cherubini, E. (2007). Signaling properties of stratum oriens interneurons in the hippocampus of transgenic mice expressing egfp in a subset of somatostatin-containing cells. *Hippocampus*, 17(7):538–53.

[Miocinovic et al., 2006] Miocinovic, S., Parent, M., Butson, C., Hahn, P., Russo, G., Vitek, J., and McIntyre, C. (2006). Computational analysis of subthalamic nucleus and lenticular fasciculus activation during therapeutic deep brain stimulation. *Journal of neurophysiology*, 96(3):1569–80.

[Miyasho et al., 2001] Miyasho, T., Takagi, H., Suzuki, H., Watanabe, S., Inoue, M., Kudo, Y., and Miyakawa, H. (2001). Low-threshold potassium channels and a low-threshold calcium channel regulate ca2+ spike firing in the dendrites of cerebellar purkinje neurons: a modeling study. *Brain research*, 891(1-2):106–15.

[Moczydlowski and Latorre, 1983] Moczydlowski, E. and Latorre, R. (1983). Gating kinetics of ca2+-activated k+ channels from rat muscle incorporated into planar lipid bilayers. evidence for two voltage-dependent ca2+ binding reactions. *The Journal of general physiology*, 82(4):511–42.

[Moradi et al., 2013] Moradi, K., Moradi, K., Ganjkhani, M., Hajihasani, M., Gharibzadeh, S., and Kaka, G. (2013). A fast model of voltage-dependent nmda receptors. *Journal of computational neuroscience*, 34(3):521–31.

[Morgan et al., 2007] Morgan, R., Santhakumar, V., and Soltesz, I. (2007). Modeling the dentate gyrus. *Progress in brain research*, 163:639–58.

[Morgan and Soltesz, 2008] Morgan, R. and Soltesz, I. (2008). Nonrandom connectivity of the epileptic dentate gyrus predicts a major role for neuronal hubs in seizures. *Proceedings of the National Academy of Sciences of the United States of America*, 105(16):6179–84.

[Morse et al., 2010] Morse, T., Carnevale, N., Mutalik, P., Migliore, M., and Shepherd, G. (2010). Abnormal excitability of oblique dendrites implicated in early alzheimer's: A computational study. *Frontiers in neural circuits*, 4.

[Munro and Kopell, 2012] Munro, E. and Kopell, N. (2012). Subthreshold somatic voltage in neocortical pyramidal cells can control whether spikes propagate from the axonal plexus to axon terminals: a model study. *Journal of neurophysiology*, 107(10):2833–52.

[Nadim et al., 1998] Nadim, F., Manor, Y., Nusbaum, M., and Marder, E. (1998). Frequency regulation of a slow rhythm by a fast periodic input. *The Journal of neuroscience : the official journal of the Society for Neuroscience*, 18(13):5053–67.

[Nakano et al., 2013] Nakano, T., Yoshimoto, J., and Doya, K. (2013). A model-based prediction of the calcium responses in the striatal synaptic spines depending on the timing of cortical and dopaminergic inputs and post-synaptic spikes. *Frontiers in computational neuroscience*, 7:119.

[Narayanan and Chattarji, 2010] Narayanan, R. and Chattarji, S. (2010). Computational analysis of the impact of chronic stress on intrinsic and synaptic excitability in the hippocampus. *Journal of neurophysiology*, 103(6):3070–83.

[Narayanan and Johnston, 2007] Narayanan, R. and Johnston, D. (2007). Long-term potentiation in rat hippocampal neurons is accompanied by spatially widespread changes in intrinsic oscillatory dynamics and excitability. *Neuron*, 56(6):1061–75.

[Narayanan and Johnston, 2010] Narayanan, R. and Johnston, D. (2010). The h current is a candidate mechanism for regulating the sliding modification threshold in a bcm-like synaptic learning rule. *Journal of neurophysiology*, 104(2):1020–33.

[Negroni and Lascano, 1996] Negroni, J. and Lascano, E. (1996). A cardiac muscle model relating sarcomere dynamics to calcium kinetics. *Journal of molecular and cellular cardiology*, 28(5):915–29.

[Nevian et al., 2007] Nevian, T., Larkum, M., Polsky, A., and Schiller, J. (2007). Properties of basal dendrites of layer 5 pyramidal neurons: a direct patch-clamp recording study. *Nature neuroscience*, 10(2):206–14.

[Neymotin et al., 2013] Neymotin, S., Hilscher, M., Moulin, T., Skolnick, Y., Lazarewicz, M., and Lytton, W. (2013). Ih tunes theta/gamma oscillations and cross-frequency coupling in an in silico ca3 model. *PloS one*, 8(10):e76285.

[Neymotin et al., 2011a] Neymotin, S., Jacobs, K., Fenton, A., and Lytton, W. (2011a). Synaptic information transfer in computer models of neocortical columns. *Journal of computational neuroscience*, 30(1):69–84.

[Neymotin et al., 2011b] Neymotin, S., Lazarewicz, M., Sherif, M., Contreras, D., Finkel, L., and Lytton, W. (2011b). Ketamine disrupts theta modulation of gamma in a computer model of hippocampus. *The Journal of neuroscience : the official journal of the Society for Neuroscience*, 31(32):11733–43.

[Neymotin et al., 2011c] Neymotin, S., Lee, H., Park, E., Fenton, A., and Lytton, W. (2011c). Emergence of physiological oscillation frequencies in a computer model of neocortex. *Frontiers in computational neuroscience*, 5:19.

[Nieus et al., 2006] Nieus, T., Sola, E., Mapelli, J., Saftenku, E., Rossi, P., and D'Angelo, E. (2006). Ltp regulates burst initiation and frequency at mossy fiber-granule cell synapses of rat cerebellum: experimental observations and theoretical predictions. *Journal of neurophysiology*, 95(2):686–99.

[O'Connor et al., 2012] O'Connor, S., Angelo, K., and Jacob, T. (2012). Burst firing versus synchrony in a gap junction connected olfactory bulb mitral cell network model. *Frontiers in computational neuroscience*, 6:75.

[Oltedal et al., 2007] Oltedal, L., Morkve, S., Veruki, M., and Hartveit, E. (2007). Patch-clamp investigations and compartmental modeling of rod bipolar axon terminals in an in vitro thin-slice preparation of the mammalian retina. *Journal of neurophysiology*, 97(2):1171–87.

[Orio et al., 2012] Orio, P., Parra, A., Madrid, R., Gonzalez, O., Belmonte, C., and Viana, F. (2012). Role of ih in the firing pattern of mammalian cold thermoreceptor endings. *Journal of neurophysiology*, 108(11):3009–23.

[Orio and Soudry, 2012] Orio, P. and Soudry, D. (2012). Simple, fast and accurate implementation of the diffusion approximation algorithm for stochastic ion channels with multiple states. *PloS one*, 7(5):e36670.

[Ostroumov, 2007] Ostroumov, K. (2007). A new stochastic tridimensional model of neonatal rat spinal motoneuron for investigating compartmentalization of neuronal conductances and their influence on firing. *Journal of neuroscience methods*, 163(2):362–72.

[Overstreet et al., 2013] Overstreet, C., Klein, J., and Helms Tillery, S. (2013). Computational modeling of direct neuronal recruitment during intracortical microstimulation in somatosensory cortex. *Journal of neural engineering*, 10(6):066016.

[Ovsepian et al., 2013] Ovsepian, S., Steuber, V., Le Berre, M., O'Hara, L., O'Leary, V., and Dolly, J. (2013). A defined heteromeric kv1 channel stabilizes the intrinsic pacemaking and regulates the output of deep cerebellar nuclear neurons to thalamic targets. *The Journal of physiology*, 591(Pt 7):1771–91.

[Palmer and Stuart, 2009] Palmer, L. and Stuart, G. (2009). Membrane potential changes in dendritic spines during action potentials and synaptic input. *The Journal of neuroscience : the official journal of the Society for Neuroscience*, 29(21):6897–903.

[Papoutsi et al., 2013] Papoutsi, A., Sidiropoulou, K., Cutsuridis, V., and Poirazi, P. (2013). Induction and modulation of persistent activity in a layer v pfc microcircuit model. *Frontiers in neural circuits*, 7:161.

[Parekh and Ascoli, 2013] Parekh, R. and Ascoli, G. (2013). Neuronal morphology goes digital: a research hub for cellular and system neuroscience. *Neuron*, 77(6):1017–38.

[Pashut et al., 2011] Pashut, T., Wolfus, S., Friedman, A., Lavidor, M., Bar-Gad, I., Yeshurun, Y., and Korngreen, A. (2011). Mechanisms of magnetic stimulation of central nervous system neurons. *PLoS computational biology*, 7(3):e1002022.

[Pinsky and Rinzel, 1994] Pinsky, P. and Rinzel, J. (1994). Intrinsic and network rhythmogenesis in a reduced traub model for ca3 neurons. *Journal of computational neuroscience*, 1(1-2):39–60.

[Poirazi et al., 2003a] Poirazi, P., Brannon, T., and Mel, B. (2003a). Arithmetic of subthreshold synaptic summation in a model ca1 pyramidal cell. *Neuron*, 37(6):977–87.

[Poirazi et al., 2003b] Poirazi, P., Brannon, T., and Mel, B. (2003b). Pyramidal neuron as two-layer neural network. *Neuron*, 37(6):989–99.

[Poleg-Polsky and Diamond, 2011] Poleg-Polsky, A. and Diamond, J. (2011). Imperfect space clamp permits electrotonic interactions between inhibitory and excitatory synaptic conductances, distorting voltage clamp recordings. *PloS one*, 6(4):e19463.

[Polsky et al., 2004] Polsky, A., Mel, B., and Schiller, J. (2004). Computational subunits in thin dendrites of pyramidal cells. *Nature neuroscience*, 7(6):621–7.

[Poolos et al., 2002] Poolos, N., Migliore, M., and Johnston, D. (2002). Pharmacological upregulation of h-channels reduces the excitability of pyramidal neuron dendrites. *Nature neuroscience*, 5(8):767–74.

[Popovic et al., 2005] Popovic, M., Djurisic, M., and Zecevic, D. (2005). Determinants of low epsp attenuation in primary dendrites of mitral cells: modeling study. *Annals of the New York Academy of Sciences*, 1048:344–8.

[Pospischil et al., 2008] Pospischil, M., Toledo-Rodriguez, M., Monier, C., Piwkowska, Z., Bal, T., Fregnac, Y., Markram, H., and Destexhe, A. (2008). Minimal hodgkin-huxley type models for different classes of cortical and thalamic neurons. *Biological cybernetics*, 99(4-5):427–41.

[Powers et al., 2012] Powers, R., Elbasiouny, S., Rymer, W., and Heckman, C. (2012). Contribution of intrinsic properties and synaptic inputs to motoneuron discharge patterns: a simulation study. *Journal of neurophysiology*, 107(3):808–23.

[Prescott and De Koninck, 2005] Prescott, S. and De Koninck, Y. (2005). Integration time in a subset of spinal lamina i neurons is lengthened by sodium and calcium currents acting synergistically to prolong subthreshold depolarization. *The Journal of neuroscience : the official journal of the Society for Neuroscience*, 25(19):4743–54.

[Proddutur et al., 2013] Proddutur, A., Yu, J., Elgammal, F., and Santhakumar, V. (2013). Seizure-induced alterations in fast-spiking basket cell gaba currents modulate frequency and coherence of gamma oscillation in network simulations. *Chaos (Woodbury, N.Y.)*, 23(4):046109.

[Quadroni and Knopfel, 1994] Quadroni, R. and Knopfel, T. (1994). Compartmental models of type a and type b guinea pig medial vestibular neurons. *Journal of neurophysiology*, 72(4):1911–24.

[Rabinowitch and Segev, 2006a] Rabinowitch, I. and Segev, I. (2006a). The endurance and selectivity of spatial patterns of long-term potentiation/depression in dendrites under homeostatic synaptic plasticity. *The Journal of neuroscience : the official journal of the Society for Neuroscience*, 26(52):13474–84.

[Rabinowitch and Segev, 2006b] Rabinowitch, I. and Segev, I. (2006b). The interplay between homeostatic synaptic plasticity and functional dendritic compartments. *Journal of neurophysiology*, 96(1):276–83.

[Rabinowitch and Segev, 2008] Rabinowitch, I. and Segev, I. (2008). Two opposing plasticity mechanisms pulling a single synapse. *Trends in neurosciences*, 31(8):377–83.

[Raman and Bean, 2001] Raman, I. and Bean, B. (2001). Inactivation and recovery of sodium currents in cerebellar purkinje neurons: evidence for two mechanisms. *Biophysical journal*, 80(2):729–37.

[Romani et al., 2013] Romani, A., Marchetti, C., Bianchi, D., Leinekugel, X., Poirazi, P., Migliore, M., and Marie, H. (2013). Computational modeling of the effects of amyloid-beta on release probability at hippocampal synapses. *Frontiers in computational neuroscience*, 7:1.

[Rossert et al., 2011] Rossert, C., Moore, L., Straka, H., and Glasauer, S. (2011). Cellular and network contributions to vestibular signal processing: impact of ion conductances, synaptic inhibition, and noise. *The Journal of neuroscience : the official journal of the Society for Neuroscience*, 31(23):8359–72.

[Rothman and Manis, 2003a] Rothman, J. and Manis, P. (2003a). Differential expression of three distinct potassium currents in the ventral cochlear nucleus. *Journal of neurophysiology*, 89(6):3070–82.

[Rothman and Manis, 2003b] Rothman, J. and Manis, P. (2003b). Kinetic analyses of three distinct potassium conductances in ventral cochlear nucleus neurons. *Journal of neurophysiology*, 89(6):3083–96.

[Rothman and Manis, 2003c] Rothman, J. and Manis, P. (2003c). The roles potassium currents play in regulating the electrical activity of ventral cochlear nucleus neurons. *Journal of neurophysiology*, 89(6):3097–113.

[Royeck et al., 2008] Royeck, M., Horstmann, M., Remy, S., Reitze, M., Yaari, Y., and Beck, H. (2008). Role of axonal nav1.6 sodium channels in action potential initiation of ca1 pyramidal neurons. *Journal of neurophysiology*, 100(4):2361–80.

[Rudolph and Destexhe, 2005] Rudolph, M. and Destexhe, A. (2005). An extended analytic expression for the membrane potential distribution of conductance-based synaptic noise. *Neural computation*, 17(11):2301–15.

[Rusakov et al., 1997] Rusakov, D., Richter-Levin, G., Stewart, M., and Bliss, T. (1997). Reduction in spine density associated with long-term potentiation in the dentate gyrus suggests a spine fusion-and-branching model of potentiation. *Hippocampus*, 7(5):489–500.

[Rusakov et al., 1996] Rusakov, D., Stewart, M., and Korogod, S. (1996). Branching of active dendritic spines as a mechanism for controlling synaptic efficacy. *Neuroscience*, 75(1):315–23.

[Safiulina et al., 2010] Safiulina, V., Caiati, M., Sivakumaran, S., Bisson, G., Migliore, M., and Cherubini, E. (2010). Control of gaba release at mossy fiber-ca3 connections in the developing hippocampus. *Frontiers in synaptic neuroscience*, 2:1.

[Safronov and Vogel, 1995] Safronov, B. and Vogel, W. (1995). Single voltage-activated na+ and k+ channels in the somata of rat motoneurones. *The Journal of physiology*, 487 (Pt 1):91–106.

[Safronov et al., 2000] Safronov, B., Wolff, M., and Vogel, W. (2000). Excitability of the soma in central nervous system neurons. *Biophysical journal*, 78(6):2998–3010.

[Saftenku, 2002] Saftenku, E. (2002). A simplified model of long-term plasticity in cerebellar mossy fiber-granule cell synapses. *Neurophysiology/Neirofiziologiya*, 34:216–218.

[Saftenku, 2005] Saftenku, E. (2005). Modeling of slow glutamate diffusion and ampa receptor activation in the cerebellar glomerulus. *Journal of theoretical biology*, 234(3):363–82.

[Saghatelyan et al., 2005] Saghatelyan, A., Roux, P., Migliore, M., Rochefort, C., Desmaisons, D., Charneau, P., Shepherd, G., and Lledo, P. (2005). Activity-dependent adjustments of the inhibitory network in the olfactory bulb following early postnatal deprivation. *Neuron*, 46(1):103–16.

[Santhakumar et al., 2005] Santhakumar, V., Aradi, I., and Soltesz, I. (2005). Role of mossy fiber sprouting and mossy cell loss in hyperexcitability: a network model of the dentate gyrus incorporating cell types and axonal topography. *Journal of neurophysiology*, 93(1):437–53.

[Saraga et al., 2006] Saraga, F., Ng, L., and Skinner, F. (2006). Distal gap junctions and active dendrites can tune network dynamics. *Journal of neurophysiology*, 95(3):1669–82.

[Saraga et al., 2003] Saraga, F., Wu, C., Zhang, L., and Skinner, F. (2003). Active dendrites and spike propagation in multi-compartment models of oriens-lacunosum/moleculare hippocampal interneurons. *The Journal of physiology*, 552(Pt 3):673–89.

[Schaefer et al., 2003a] Schaefer, A., Helmstaedter, M., Sakmann, B., and Korngreen, A. (2003a). Correction of conductance measurements in non-space-clamped structures: 1. voltage-gated k+ channels. *Biophysical journal*, 84(6):3508–28.

[Schaefer et al., 2007] Schaefer, A., Helmstaedter, M., Schmitt, A., Bar-Yehuda, D., Almog, M., Ben-Porat, H., Sakmann, B., and Korngreen, A. (2007). Dendritic voltage-gated k+ conductance gradient in pyramidal neurones of neocortical layer 5b from rats. *The Journal of physiology*, 579(Pt 3):737–52.

[Schaefer et al., 2003b] Schaefer, A., Larkum, M., Sakmann, B., and Roth, A. (2003b). Coincidence detection in pyramidal neurons is tuned by their dendritic branching pattern. *Journal of neurophysiology*, 89(6):3143–54.

[Schmidt-Hieber and Bischofberger, 2010] Schmidt-Hieber, C. and Bischofberger, J. (2010). Fast sodium channel gating supports localized and efficient axonal action potential initiation. *The Journal of neuroscience : the official journal of the Society for Neuroscience*, 30(30):10233–42.

[Schmidt-Hieber and Hausser, 2013] Schmidt-Hieber, C. and Hausser, M. (2013). Cellular mechanisms of spatial navigation in the medial entorhinal cortex. *Nature neuroscience*, 16(3):325–31.

[Schmidt-Hieber et al., 2007] Schmidt-Hieber, C., Jonas, P., and Bischofberger, J. (2007). Subthreshold dendritic signal processing and coincidence detection in dentate gyrus granule cells. *The Journal of neuroscience : the official journal of the Society for Neuroscience*, 27(31):8430–41.

[Shah et al., 2011] Shah, M., Migliore, M., and Brown, D. (2011). Differential effects of kv7 (m-) channels on synaptic integration in distinct subcellular compartments of rat hippocampal pyramidal neurons. *The Journal of physiology*, 589(Pt 24):6029–38.

[Shah et al., 2008] Shah, M., Migliore, M., Valencia, I., Cooper, E., and Brown, D. (2008). Functional significance of axonal kv7 channels in hippocampal pyramidal neurons. *Proceedings of the National Academy of Sciences of the United States of America*, 105(22):7869–74.

[Sheasby and Fohlmeister, 1999] Sheasby, B. and Fohlmeister, J. (1999). Impulse encoding across the dendritic morphologies of retinal ganglion cells. *Journal of neurophysiology*, 81(4):1685–98.

[Shen et al., 1999] Shen, G., Chen, W., Midtgaard, J., Shepherd, G., and Hines, M. (1999). Computational analysis of action potential initiation in mitral cell soma and dendrites based on dual patch recordings. *Journal of neurophysiology*, 82(6):3006–20.

[Shepherd and Brayton, 1979] Shepherd, G. and Brayton, R. (1979). Computer simulation of a dendrodendritic synaptic circuit for self- and lateral-inhibition in the olfactory bulb. *Brain research*, 175(2):377–82.

[Shimazaki et al., 2006] Shimazaki, R., Boccaccio, A., Mazzatenta, A., Pinato, G., Migliore, M., and Menini, A. (2006). Electrophysiological properties and modeling of murine vomeronasal sensory neurons in acute slice preparations. *Chemical senses*, 31(5):425–35.

[Shu et al., 2007a] Shu, Y., Duque, A., Yu, Y., Haider, B., and McCormick, D. (2007a). Properties of action-potential initiation in neocortical pyramidal cells: evidence from whole cell axon recordings. *Journal of neurophysiology*, 97(1):746–60.

[Shu et al., 2006] Shu, Y., Hasenstaub, A., Duque, A., Yu, Y., and McCormick, D. (2006). Modulation of intracortical synaptic potentials by presynaptic somatic membrane potential. *Nature*, 441(7094):761–5.

[Shu et al., 2007b] Shu, Y., Yu, Y., Yang, J., and McCormick, D. (2007b). Selective control of cortical axonal spikes by a slowly inactivating k+ current. *Proceedings of the National Academy of Sciences of the United States of America*, 104(27):11453–8.

[Sidiropoulou and Poirazi, 2012] Sidiropoulou, K. and Poirazi, P. (2012). Predictive features of persistent activity emergence in regular spiking and intrinsic bursting model neurons. *PLoS computational biology*, 8(4):e1002489.

[Sikora et al., 2005] Sikora, M., Gottesman, J., and Miller, R. (2005). A computational model of the ribbon synapse. *Journal of neuroscience methods*, 145(1-2):47–61.

[Simoes de Souza and De Schutter, 2011] Simoes de Souza, F. and De Schutter, E. (2011). Robustness effect of gap junctions between golgi cells on cerebellar cortex oscillations. *Neural systems & circuits*, 1(1):7.

[Simon et al., 1999] Simon, J., Carr, C., and Shamma, S. (1999). A dendritic model of coincidence detection in the avian brainstem. *Neurocomputing*, 26-27:263–9.

[Skaliora et al., 1995] Skaliora, I., Robinson, D., Scobey, R., and Chalupa, L. (1995). Properties of k+ conductances in cat retinal ganglion cells during the period of activity-mediated refinements in retinofugal pathways. *The European journal of neuroscience*, 7(7):1558–68.

[Solinas et al., 2007a] Solinas, S., Forti, L., Cesana, E., Mapelli, J., De Schutter, E., and D'Angelo, E. (2007a). Computational reconstruction of pacemaking and intrinsic electroresponsiveness in cerebellar golgi cells. *Frontiers in cellular neuroscience*, 1:2.

[Solinas et al., 2007b] Solinas, S., Forti, L., Cesana, E., Mapelli, J., De Schutter, E., and D'Angelo, E. (2007b). Fast-reset of pacemaking and theta-frequency resonance patterns in cerebellar golgi cells: simulations of their impact in vivo. *Frontiers in cellular neuroscience*, 1:4.

[Somjen et al., 2008] Somjen, G., Kager, H., and Wadman, W. (2008). Computer simulations of neuron-glia interactions mediated by ion flux. *Journal of computational neuroscience*, 25(2):349–65.

[Sousa et al., 2014] Sousa, M., Szucs, P., Lima, D., and Aguiar, P. (2014). The pronociceptive dorsal reticular nucleus contains mostly tonic neurons and shows a high prevalence of spontaneous activity in block preparation. *Journal of neurophysiology*, 111(7):1507–18.

[Spampanato et al., 2004] Spampanato, J., Aradi, I., Soltesz, I., and Goldin, A. (2004). Increased neuronal firing in computer simulations of sodium channel mutations that cause generalized epilepsy with febrile seizures plus. *Journal of neurophysiology*, 91(5):2040–50.

[Spiga et al., 2010] Spiga, S., Lintas, A., Migliore, M., and Diana, M. (2010). Altered architecture and functional consequences of the mesolimbic dopamine system in cannabis dependence. *Addiction biology*, 15(3):266–76.

[Stacey et al., 2011] Stacey, W., Krieger, A., and Litt, B. (2011). Network recruitment to coherent oscillations in a hippocampal computer model. *Journal of neurophysiology*, 105(4):1464–81.

[Stacey et al., 2009] Stacey, W., Lazarewicz, M., and Litt, B. (2009). Synaptic noise and physiological coupling generate high-frequency oscillations in a hippocampal computational model. *Journal of neurophysiology*, 102(4):2342–57.

[Steephen and Manchanda, 2009] Steephen, J. and Manchanda, R. (2009). Differences in biophysical properties of nucleus accumbens medium spiny neurons emerging from inactivation of inward rectifying potassium currents. *Journal of computational neuroscience*, 27(3):453–70.

[Stehle et al., 1990] Stehle, G., Buss, J., Eibach, J., Plugge, T., Lasserre, J., Kehry, I., and Heene, D. (1990). Cardiogenic shock associated with verapamil in a patient with liver cirrhosis. *Lancet (London, England)*, 336(8722):1079.

[Sterratt et al., 2012] Sterratt, D., Groen, M., Meredith, R., and van Ooyen, A. (2012). Spine calcium transients induced by synaptically-evoked action potentials can predict synapse location and establish synaptic democracy. *PLoS computational biology*, 8(6):e1002545.

[Stockbridge and Moore, 1984] Stockbridge, N. and Moore, J. (1984). Dynamics of intracellular calcium and its possible relationship to phasic transmitter release and facilitation at the frog neuromuscular junction. *The Journal of neuroscience : the official journal of the Society for Neuroscience*, 4(3):803–11.

[Stuart and Spruston, 1998] Stuart, G. and Spruston, N. (1998). Determinants of voltage attenuation in neocortical pyramidal neuron dendrites. *The Journal of neuroscience : the official journal of the Society for Neuroscience*, 18(10):3501–10.

[Tejada et al., 2014] Tejada, J., Garcia-Cairasco, N., and Roque, A. (2014). Combined role of seizure-induced dendritic morphology alterations and spine loss in newborn granule cells with mossy fiber sprouting on the hyperexcitability of a computer model of the dentate gyrus. *PLoS computational biology*, 10(5):e1003601.

[Thomas and Petrou, 2013] Thomas, E. and Petrou, S. (2013). Network-specific mechanisms may explain the paradoxical effects of carbamazepine and phenytoin. *Epilepsia*, 54(7):1195–202.

[Torben-Nielsen et al., 2012] Torben-Nielsen, B., Segev, I., and Yarom, Y. (2012). The generation of phase differences and frequency changes in a network model of inferior olive subthreshold oscillations. *PLoS computational biology*, 8(7):e1002580.

[Traub et al., 2003] Traub, R., Buhl, E., Gloveli, T., and Whittington, M. (2003). Fast rhythmic bursting can be induced in layer 2/3 cortical neurons by enhancing persistent na+ conductance or by blocking bk channels. *Journal of neurophysiology*, 89(2):909–21.

[Traub et al., 2005a] Traub, R., Contreras, D., Cunningham, M., Murray, H., LeBeau, F., Roopun, A., Bibbig, A., Wilent, W., Higley, M., and Whittington, M. (2005a). Single-column thalamocortical network model exhibiting gamma oscillations, sleep spindles, and epileptogenic bursts. *Journal of neurophysiology*, 93(4):2194–232.

[Traub et al., 2005b] Traub, R., Contreras, D., and Whittington, M. (2005b). Combined experimental/simulation studies of cellular and network mechanisms of epileptogenesis in vitro and in vivo. *Journal of clinical neurophysiology : official publication of the American Electroencephalographic Society*, 22(5):330–42.

[Tseng et al., 2008] Tseng, S., Tsai, L., and Yeh, S. (2008). Induction of high-frequency oscillations in a junction-coupled network. *The Journal of neuroscience : the official journal of the Society for Neuroscience*, 28(28):7165–73.

[Tsodyks et al., 1998] Tsodyks, M., Pawelzik, K., and Markram, H. (1998). Neural networks with dynamic synapses. *Neural computation*, 10(4):821–35.

[Tsutsui and Oka, 2002] Tsutsui, H. and Oka, Y. (2002). Slow removal of na(+) channel inactivation underlies the temporal filtering property in the teleost thalamic neurons. *The Journal of physiology*, 539(Pt 3):743–53.

[Uebachs et al., 2010] Uebachs, M., Opitz, T., Royeck, M., Dickhof, G., Horstmann, M., Isom, L., and Beck, H. (2010). Efficacy loss of the anticonvulsant carbamazepine in mice lacking sodium channel beta subunits via paradoxical effects on persistent sodium currents. *The Journal of neuroscience : the official journal of the Society for Neuroscience*, 30(25):8489–501.

[van der Velden et al., 2012] van der Velden, L., van Hooft, J., and Chameau, P. (2012). Altered dendritic complexity affects firing properties of cortical layer 2/3 pyramidal neurons in mice lacking the 5-ht3a receptor. *Journal of neurophysiology*, 108(5):1521–8.

[van Elburg and van Ooyen, 2009] van Elburg, R. and van Ooyen, A. (2009). Generalization of the event-based carnevale-hines integration scheme for integrate-and-fire models. *Neural computation*, 21(7):1913–30.

[van Elburg and van Ooyen, 2010] van Elburg, R. and van Ooyen, A. (2010). Impact of dendritic size and dendritic topology on burst firing in pyramidal cells. *PLoS computational biology*, 6(5):e1000781.

[van Welie et al., 2006] van Welie, I., Remme, M., van Hooft, J., and Wadman, W. (2006). Different levels of ih determine distinct temporal integration in bursting and regular-spiking neurons in rat subiculum. *The Journal of physiology*, 576(Pt 1):203–14.

[Varela et al., 1997] Varela, J., Sen, K., Gibson, J., Fost, J., Abbott, L., and Nelson, S. (1997). A quantitative description of short-term plasticity at excitatory synapses in layer 2/3 of rat primary visual cortex. *The Journal of neuroscience : the official journal of the Society for Neuroscience*, 17(20):7926–40.

[Vazquez et al., 2009] Vazquez, Y., Mendez, B., Trueta, C., and De-Miguel, F. (2009). Summation of excitatory postsynaptic potentials in electrically-coupled neurones. *Neuroscience*, 163(1):202–12.

[Vierling-Claassen et al., 2010] Vierling-Claassen, D., Cardin, J., Moore, C., and Jones, S. (2010). Computational modeling of distinct neocortical oscillations driven by cell-type selective optogenetic drive: separable resonant circuits controlled by low-threshold spiking and fast-spiking interneurons. *Frontiers in human neuroscience*, 4:198.

[Vitko et al., 2005] Vitko, I., Chen, Y., Arias, J., Shen, Y., Wu, X., and Perez-Reyes, E. (2005). Functional characterization and neuronal modeling of the effects of childhood absence epilepsy variants of cacna1h, a t-type calcium channel. *The Journal of neuroscience : the official journal of the Society for Neuroscience*, 25(19):4844–55.

[Vladimirov et al., 2012] Vladimirov, N., Tu, Y., and Traub, R. (2012). Shortest loops are pacemakers in random networks of electrically coupled axons. *Frontiers in computational neuroscience*, 6:17.

[Vladimirov et al., 2013] Vladimirov, N., Tu, Y., and Traub, R. (2013). Synaptic gating at axonal branches, and sharp-wave ripples with replay: a simulation study. *The European journal of neuroscience*, 38(10):3435–47.

[Vogels and Abbott, 2005] Vogels, T. and Abbott, L. (2005). Signal propagation and logic gating in networks of integrate-and-fire neurons. *The Journal of neuroscience : the official journal of the Society for Neuroscience*, 25(46):10786–95.

[Wang et al., 2002] Wang, J., Chen, S., Nolan, M., and Siegelbaum, S. (2002). Activity-dependent regulation of hcn pacemaker channels by cyclic amp: signaling through dynamic allosteric coupling. *Neuron*, 36(3):451–61.

[Wang et al., 2013] Wang, K., Riera, J., Enjieu-Kadji, H., and Kawashima, R. (2013). The role of extracellular conductivity profiles in compartmental models for neurons: particulars for layer 5 pyramidal cells. *Neural computation*, 25(7):1807–52.

[Wang et al., 1998] Wang, L., Gan, L., Forsythe, I., and Kaczmarek, L. (1998). Contribution of the kv3.1 potassium channel to high-frequency firing in mouse auditory neurones. *The Journal of physiology*, 509 (Pt 1):183–94.

[Wang and Buzsaki, 1996] Wang, X. and Buzsaki, G. (1996). Gamma oscillation by synaptic inhibition in a hippocampal interneuronal network model. *The Journal of neuroscience : the official journal of the Society for Neuroscience*, 16(20):6402–13.

[Wang et al., 1996] Wang, X., McKenzie, J., and Kemm, R. (1996). Whole-cell k+ currents in identified olfactory bulb output neurones of rats. *The Journal of physiology*, 490 (Pt 1):63–77.

[Wang et al., 1991] Wang, X., Rinzel, J., and Rogawski, M. (1991). A model of the t-type calcium current and the low-threshold spike in thalamic neurons. *Journal of neurophysiology*, 66(3):839–50.

[Watanabe et al., 2002] Watanabe, S., Hoffman, D., Migliore, M., and Johnston, D. (2002). Dendritic k+ channels contribute to spike-timing dependent long-term potentiation in hippocampal pyramidal neurons. *Proceedings of the National Academy of Sciences of the United States of America*, 99(12):8366–71.

[Weaver and Wearne, 2006] Weaver, C. and Wearne, S. (2006). The role of action potential shape and parameter constraints in optimization of compartment models. *Neurocomputing*, 69:1053–1057.

[Welday et al., 2011] Welday, A., Shlifer, I., Bloom, M., Zhang, K., and Blair, H. (2011). Cosine directional tuning of theta cell burst frequencies: evidence for spatial coding by oscillatory interference. *The Journal of neuroscience : the official journal of the Society for Neuroscience*, 31(45):16157–76.

[Williams and Stuart, 2000] Williams, S. and Stuart, G. (2000). Action potential backpropagation and somato-dendritic distribution of ion channels in thalamocortical neurons. *The Journal of neuroscience : the official journal of the Society for Neuroscience*, 20(4):1307–17.

[Wilson and Cowan, 1972] Wilson, H. and Cowan, J. (1972). Excitatory and inhibitory interactions in localized populations of model neurons. *Biophysical journal*, 12(1):1–24.

[Wimmer et al., 2010] Wimmer, V., Reid, C., Mitchell, S., Richards, K., Scaf, B., Leaw, B., Hill, E., Royeck, M., Horstmann, M., Cromer, B., Davies, P., Xu, R., Lerche, H., Berkovic, S., Beck, H., and Petrou, S. (2010). Axon initial segment dysfunction in a mouse model of genetic epilepsy with febrile seizures plus. *The Journal of clinical investigation*, 120(8):2661–71.

[Winograd et al., 2008] Winograd, M., Destexhe, A., and Sanchez-Vives, M. (2008). Hyperpolarization-activated graded persistent activity in the prefrontal cortex. *Proceedings of the National Academy of Sciences of the United States of America*, 105(20):7298–303.

[Wolf et al., 2005] Wolf, J., Moyer, J., Lazarewicz, M., Contreras, D., Benoit-Marand, M., O'Donnell, P., and Finkel, L. (2005). Nmda/ampa ratio impacts state transitions and entrainment to oscillations in a computational model of the nucleus accumbens medium spiny projection neuron. *The Journal of neuroscience : the official journal of the Society for Neuroscience*, 25(40):9080–95.

[Yu et al., 2013a] Yu, J., Proddutur, A., Elgammal, F., Ito, T., and Santhakumar, V. (2013a). Status epilepticus enhances tonic gaba currents and depolarizes gaba reversal potential in dentate fast-spiking basket cells. *Journal of neurophysiology*, 109(7):1746–63.

[Yu et al., 2013b] Yu, Y., McTavish, T., Hines, M., Shepherd, G., Valenti, C., and Migliore, M. (2013b). Sparse distributed representation of odors in a large-scale olfactory bulb circuit. *PLoS computational biology*, 9(3):e1003014.

[Yu et al., 2008] Yu, Y., Shu, Y., and McCormick, D. (2008). Cortical action potential backpropagation explains spike threshold variability and rapid-onset kinetics. *The Journal of neuroscience : the official journal of the Society for Neuroscience*, 28(29):7260–72.

[Zhou and Hablitz, 1996] Zhou, F. and Hablitz, J. (1996). Layer i neurons of the rat neocortex. ii. voltage-dependent outward currents. *Journal of neurophysiology*, 76(2):668–82.

[Zhou et al., 2005] Zhou, Y., Carney, L., and Colburn, H. (2005). A model for interaural time difference sensitivity in the medial superior olive: interaction of excitatory and inhibitory synaptic inputs, channel dynamics, and cellular morphology. *The Journal of neuroscience : the official journal of the Society for Neuroscience*, 25(12):3046–58.

[Zhou and Colburn, 2010] Zhou, Y. and Colburn, H. (2010). A modeling study of the effects of membrane afterhyperpolarization on spike interval statistics and on ild encoding in the lateral superior olive. *Journal of neurophysiology*, 103(5):2355–71.

[Zhu et al., 1999a] Zhu, J., Lytton, W., Xue, J., and Uhlrich, D. (1999a). An intrinsic oscillation in interneurons of the rat lateral geniculate nucleus. *Journal of neurophysiology*, 81(2):702–11.

[Zhu et al., 1999b] Zhu, J., Uhlrich, D., and Lytton, W. (1999b). Burst firing in identified rat geniculate interneurons. *Neuroscience*, 91(4):1445–60.
